# Sub-consensus haploid variant calling in long-read sequencing technology

**DOI:** 10.1371/journal.pone.0356259

**Published:** 2026-10-05

**Authors:** Xavier Zair, Andreas Wilm, Miles C. Benton, Cheng Yong Tham, Lin Yang, Paola Florez de Sessions, October Michael Sessions, Eng Hui Chew, Swapnil Mishra

**Affiliations:** 1 National University of Singapore, Saw Swee hock School of Public Health, Singapore, Singapore; 2 ImmunoScape, Pte Ltd, Singapore, Singapore; 3 Oxford Nanopore Technologies, Oxford, UK; 4 Department of Pharmacy, National University of Singapore, Singapore, Singapore; Al Muthanna University, IRAQ

## Abstract

**Background:**

Next-generation sequencing (NGS) is vital for tracking viral evolution during outbreaks. Oxford Nanopore Technologies (ONT) offers long-read, cost-effective sequencing, but accurate sub-consensus variant detection remains challenging. LoFreq, a widely used caller for sub-consensus variant calling in haploid genomes, was designed for short reads. This study assesses and benchmarks LoFreq variant identification accuracy on long-read data and proposes a calibration method to improve accuracy.

**Methods:**

We created truth sets using three SARS-CoV-2 spike gene plasmids (7179 bases, 100 SNVs) and whole Escherichia coli genomes. Libraries were sequenced using R9.4.1 and R10.4.1 flow cells. LoFreq’s recall was benchmarked across chemistries and library sizes. We also introduced a method to adjust Phred quality scores for enhanced accuracy.

**Results:**

LoFreq showed high sensitivity, detecting variants at frequencies as low as 0.1%, with best performance on R10.4.1. However, false discovery rates (FDR) were chemistry- and depth-dependent. Our Phred score calibration reduced false positives while maintaining recall, though it was less effective below 10% frequency and not suitable for structural variant detection.

**Conclusion:**

LoFreq can detect sub-consensus variants in ONT data, but high FDR limits direct use. Our calibration method improves accuracy, offering a simple Phred score adjustment approach that reduces false discovery rates in ONT data.

## Introduction

Next generation sequencing (NGS) has become instrumental in epidemiology in recent years, with applications ranging from cancer screening to evolutionary studies [1,2]. During the COVID-19 pandemic, whole-genome sequencing (WGS) emerged as a critical tool for epidemiological surveillance [2–4]. At the time of writing, approximately 17 million genome sequences have been uploaded to The Global Initiative on Sharing All Influenza Data (GISAID) alone. This unprecedented level of data generation and sharing is expected to increase further with the growing accessibility of NGS, particularly in future outbreak scenarios.

Generated genomes have been invaluable in tracking mutations and viral evolution, influencing policy and interventions such as vaccine development [3,5–8]. Data analysis pipelines and workflows for NGS have advanced significantly, producing accurate viral consensus genomes [9–11]. However, beyond consensus level analysis, there has been renewed interest in investigating sub-consensus variants- those present at frequencies below the consensus threshold of > 50%. This area of research has been emerging as a means to elucidate viral micro-evolution, drug resistance emergence, and immune evasion mechanisms relevant to vaccine efficacy [7,12–14].

Oxford Nanopore Technologies (ONT) sequencing technology has captured a significant share of the NGS market in recent years. Factors such as long-read capabilities, cost-effectiveness, and ease of use have contributed to this growth [15,16]. It has been estimated that about a fifth of SARS-CoV2 NGS reads uploaded to online data repositories were generated on ONT platforms [2]. Although consensus level genome generation on ONT platforms has reached significant maturity, accurately characterising variants of lower-frequencies is challenging, particularly for longer read technologies due to increased noise levels [17,18].Polishing methods have proven viable for increasing overall accuracy substantially. However, they often demand laborious analysis and may compromise depth, overlooking sub-consensus variants [19], and in some cases, require additional short read data [20]. Emerging error correction tools, such as HERRO, are not able to be applied to shorter reads (<4,096 bp), limiting their application to data sets with longer reads at this point in time [21]. Some pipelines resort to blacklisting complex genomic regions to reduce noise, but this approach can compromise resolution, and existing variant analysis tools often exhibit poor single nucleotide variant (SNV) discovery at low frequencies [7,22]. Widely used pipelines like ARTIC-ONT and COG-UK focus on accurate consensus genome generation, limiting variant population analysis to experimental modes [23,24]. Instead of correcting errors at the read level, some variant calling toolkits filter variants post-calling, targeting error patterns inherent to long-read sequencing [17,25,26]. However, these solutions are often genus specific and generalizing to other domains can be challenging.

Presently, tools for precise identification of sub-consensus variants in haploid genomes from fourth generation NGS data are lacking. Diploid germline variant calling is well-served by dedicated tools such as Clair3 [27] and DeepVariant [28], while Longshot [29] targets long-read diploid SNP calling specifically — none of which are suited for population-level sub-consensus variant analysis in haploid genomes. Even viral-specific callers such as iVar [30], designed for amplicon-based intrahost diversity measurement, rely on cross-replicate concordance to filter false positives, an assumption that inherently misclassifies unique variants as noise. LoFreq’s most notable feature is rare variant calling in haploid genomes, whereas other callers often misclassify or discard variants occurring at frequencies 20% and below. Unfortunately, LoFreq was developed for short read data of higher quality and has not undergone formal testing on long read platforms [31]. Polishing to increase read quality is not feasible since it may remove the sub-consensus variants of interests. Traditionally, variant caller validation studies insert variants in silico, artificially modifying bases in NGS datasets for benchmarking purposes [17,32]. While this method offers flexibility, it may fail to accurately represent real biological events and cannot account for the sequencing biases intrinsic to each technology [33]. The alternative approach to benchmark variant calling performance by comparing shared variants may be inaccurate due to inherent errors in Illumina reads [34]. Relying on cross-concordance, this method frequently misses errors in Illumina reads, as the ground truth is uncertain.

This study aims to benchmark the performance of the variant caller, LoFreq, on long-read data utilizing a curated truth set with known variant compositions. Specifically, we intend to benchmark the recall of variants in shorter plasmid reads, which emulate viral genomes, and in full-length *Escherichia coli* genomes to enable indel recall analysis. Additionally, we propose a calibration method aimed at reducing base quality (or Phred) scores to enhance accuracy in long-read datasets. Our findings indicate that while LoFreq remains useful for sub-consensus variant calling in long-read chemistry, the high rate of false discoveries is a significant drawback. However, with quality score adjustment, sub-consensus variants can still be detected confidently, while significantly reducing false discoveries across our datasets. As nanopore sequencing becomes more cost-effective and portable, the proportion of pathogen data generated on these platforms is anticipated to rise in future outbreaks. This study aims to address the lack of sub-consensus variant calling tools for long-read datasets by proposing workarounds to effectively apply LoFreq to nanopore reads.

## Methods

### Truth set construction

Construction of truth set libraries followed two distinct approaches. The first approach utilised plasmids on a pUNO backbone that contained the Delta variant SARS-CoV-2 spike gene (InVivogen, p1-spike-v8, 7179 base-pairs). Two distinct chimeric plasmids were generated by replacing a 241-base-pair segment (between nucleotides 1,750−1,990) with the original Wuhan-wet market (wild-type, Clade 19) and Omicron variant spike genes. Chimeric inserts from wild-type and Omicron variants were PCR-amplified from their donor plasmids. Inserts from recipient plasmids were excised via restriction digestion with *KpnI* and *NotI* (New England Biolabs), and donor amplicons were incorporated using Gibson assembly (New England Biolabs, NEBuilder^®^ HiFi DNA Assembly Master Mix). Comparative alignment of the plasmids indicates an 85-base-pair difference. Of the 85 variants, 70 display a single nucleotide difference across all three spike plasmids. Conversely, the remaining 15 positions offer more than two possibilities, for instance, A, T, and G from Delta, Omicron, and wild-type respectively. When these three plasmids are combined, the cumulative count of detectable variants adds up to 100 (70 + 15 × 2). This design evaluates both the variant caller’s recognition of distinct variants and its differentiation ability among three nucleotide options at specific positions. Sanger sequencing verified the accurate integration of chimeric sequences. Plasmids were expanded natively on K-12 DH5α (New England Biolabs) strains and purified by Miniprep (QIAprep Spin Miniprep Kit, Qiagen). Plasmid sequences were verified with Sanger sequencing, reads available study repository under sanger_plasmids.

The alternate strategy targeted the sequencing of entire genomes from two *Escherichia coli* K12 strains. Full bacterial genome analysis facilitated for identification of insertional and deletional variants not found in the plasmid models. Furthermore, the longer genomes allowed for homopolymer and k-mer signature analysis which are less abundant in smaller libraries. Two strains were selected for this purpose, HB101 (Life Technologies, Max Efficiency HB101 Competent Cell) and DH5α (NEB® 5-alpha Competent *E. coli* High Efficiency), which showed a high pairwise similarity of 95.2%. Between the two selected E.coli K-12 strains, a total of 1496 substitutional variants, 13770 insertions, and 47990 deletions were identified through pairwise alignment, with an expected frequency of 10 percent. Each strain was grown overnight in Lennox LB broth base (ThermoFisher Scientific) at 37°C and genomes were subsequently extracted and purified using the PureLink™ Genomic DNA Mini Kit (Invitrogen).

### Sequencing and flow cell chemistry

To evaluate the performance of the updated pore chemistry, libraries were sequenced on R9.4.1 and R10.4.1 flow cells. Spike plasmid ratios were precisely stoichiometrically mixed, targeting allele frequencies (AF) of 1%, 5%, 10%, 15% and 19%. Bacterial genomes were mixed exclusively at a frequency of 10%. R9.4.1 libraries were prepared using ONT’s ligation sequencing kit SQT-LSK109 and barcoded with native barcode expansions 1–12 (EXP-NBD104). Libraries for R10.4.1 flow cells were generated using the Native Barcoding Kit 24 (SQK-NBD114.24). Each of the three plasmids received individual barcoding before undergoing sequencing on a singular flow cell. Each bacterial strain was sequenced on a dedicated flow cell, while a separate flow cell was used for the mixed sample preparation. The flow cells were run for 72 hours to achieve maximal depth on ONT’s GridION Mk1 system. Software versions: MinKNOW; 22.03.4, Bream; 7.0.9, Configuration; 5.0.8, Guppy; 6.0.7, MinKNOW Core 5.0.0.

### Bioinformatic analysis

Raw sequence files (fast5 and pod5) were base-called using Dorado (https://github.com/wdecoster/nanofilt; v0.5.3) using the super accurate model (SUP v4.2.0). Mean read lengths: plasmids (R9.4.1: 7.11kb, R10.4.1: 7.27kb); genomes (R9.4.1: 7.79kb, R10.4.1: 7.21kb). Basecalling was performed natively on the GridION Mk1 system. ‘Passed’ reads were filtered using the NanoFilt (https://github.com/wdecoster/nanofilt; v2.8.0) package, with a minimum read quality cut-off of 10. Adaptors and barcodes were trimmed using porechop (https://github.com/rrwick/Porechop; v0.2.3_seqan2.1.1), -auto setting, allowing porechop to automatically detect and trim detected barcodes. A consensus for the bacterial genomes was generated using de novo assembly with the Flye (https://github.com/mikolmogorov/Flye; v2.9) assembler with the ‘--nano-raw denovo’ settings. This served as a baseline for variant identification between K-12 strains. Consensus genomes were aligned pairwise using MAFFT (v7.310) with the --auto setting. The resulting de novo reference alignments (genomes) and in-silico references (plasmids) were annotated using the Geneious Prime suite (Java Version 11.0.20.1 + 1) to generate a list of expected variants used for benchmarking. Reference-based alignment was carried out using Minimap2 (https://github.com/lh3/minimap2; v2.17-r941), with the ‘-ax map-ont’ parameters. Samtools (v1.6) was used for the conversion of SAM to BAM, as well as for indexing and sorting of alignment files.

To ensure consistency, reads were normalised to standard depths, as depth was suspected to be a critical variable in variant calling. Datasets were normalised to a consistent depth, set by the dataset with the minimum depth. To mitigate sampling error, all filtered and trimmed reads underwent twelve rounds of subsampling across all sequencing chemistries, facilitated by the Rasusa (https://github.com/mbhall88/rasusa; v0.6.1) script, with the reference length as the input. No fixed random seed was applied; subsampling was therefore stochastic across iterations, with run-to-run variability captured by the 95% confidence intervals. Twelve replicates were selected in accordance with established benchmarking practice [35–37]; empirical bootstrap analysis of our plasmid recall distributions confirmed that mean CI width at n = 12 (7.13) was 38% narrower than at n = 6 (11.57), while further increases yielded diminishing returns. Bacterial genome datasets necessitated a more targeted, reference-based method of depth normalisation due to their significantly longer genomes. This was achieved using the Jvarkit/ Biostars154220 toolkit (github.com/lindenb/jvarkit; v4b65b20b2) with the following parameters, substituting SAM alignments as input:

java -jar./jvarkit.jar biostar154220 -d <target_depth(int)> -o <output.sam> <input.sam>

This operation removes all reads exceeding the target depth, retaining only those reads with the highest mapping quality. For indel analysis on bacterial genomes, a uniform indel quality of 50 was added to BAM files using LoFreq indelqual with parameters -u 50. Variant calling with LoFreq (v2.1.3.1) [31], was executed with -B mode, thereby disabling BAQ computation, which is optimized for shorter Illumina reads [38]. Alignment quality, coverage and depth information were verified with the Qualimap (v.2.2.2-dev), using the bamqc parameter. Longshot [29] (v0.4.1) and Clair3 [27] (v0.1-r10) were also used to call variants, which will be briefly elaborated in the results.

### Quality score analysis and adjustment

Reads from clonal plasmids were trimmed and filtered according to the previously mentioned settings. Random extraction of 1 million bases from the processed FASTQ files was performed over 12 iterations using Rasusa’s base extraction parameter (-b). To assess Phred score accuracy, both the distribution and empirical accuracy of the basecaller was analyzed. Firstly, Phred scores addigned by basecaller (encoded Phred scores) were extracted from subsampled FASTQ files, using the BBmaps suite of toolkits (v38.18). This was executed under the reformat.sh function’s, qchist parameter with ‘maxcalledquality=90’.. CSV files containing counts of total bases at each Phred value were generated. Phred distribution graphs were created from the resultant CSV files (Fig 5a). Secondly, to determine the empirical error rates when reads are aligned to their reference sequences, SAM alignment filles were generated from subsampled FASTQ files. Alignment was performed using minimap2 at the above-mentioned settings. The resultant SAM files were analysed with BBmaps reformat.sh, this time with the qahist parameter. The functions extract the encoded Phred scores and generated corresponding empirical accuracy values, which were used to generate quality-versus-quality QvsQ plots (Fig 3b, 3c).

For Phred score calibration, 2 approaches were tested: fitted calibration and uniform scalar reduction. For fitted calibration, the relationship between encoded and empirical Phred scores from the above mentioned QvsQ plots was modeled using quadratic (y = ax² + bx + c), higher-order polynomial (up to n = 4), and Gaussian (y = Ae^(-(x-μ)²/2σ²)) functions. Curve fits were evaluated using the coefficient of determination (R²) and root mean squared error (RMSE), with all three curve types achieving R² > 0.95 on clonal plasmid QvsQ data. The resulting equations were used to generate lookup tables that mapped each encoded Phred score to its empirically determined corrected values. These lookup tables were applied uniformly to all bases in the FASTQ files regardless of sequence context or genomic position.For scalar reduction, all Phred scores were uniformly decreased by a specified value (e.g., a reduction of 1 lowered all scores by 1, a reduction of 2 lowered all scores by 2), with reductions ranging from 1 to 8 for genomic datasets and 1–10 for plasmid datasets. Both calibration methods were implemented using the custom script, QUAD (v0.2) [39], which modifies base quality scores in FASTQ files based on input CSV files containing “before” and “after” Phred values. Calibration CSV files for each curve fit can be found at https://github.com/x-zair/ONT_truthset. A control set was also established, maintaining unchanged Phred values to rule out file manipulation or corruption as causes of reduced false positives, attributing the effect to actual change. The adjusted BAM files were indexed using samtools, followed by variant calling with LoFreq under the previously described parameters.

### Statistical analysis

Parametric statistical methods were used throughout following confirmation of data normality via Shapiro-Wilk testing (*W* > 0.90, *p* > 0.05). Differences in false discovery rates and recall between flow cell chemistries (R9.4.1 vs R10.4.1) and across sequencing depths were assessed using unpaired two-sample t-tests. Paired t-tests were applied for within-chemistry Phred reduction comparisons in bacterial genomic datasets. Linear regression analysis was used to assess continuous relationships between Phred score reduction levels and false positive counts, with goodness-of-fit evaluated using R² values. Effect sizes for Phred reduction efficacy were quantified using Cohen’s d for paired samples. For comparisons across multiple sequencing depths, one-way analysis of variance (ANOVA) was performed, followed by post-hoc Tukey’s HSD tests for pairwise comparisons where appropriate.

Descriptive statistics are reported as means with 95% confidence intervals (CI), calculated using the t-distribution: n = 12 subsampling replicates (df = 11, t-critical = 2.201) for mixed and genomic datasets, and n = 3 independent clonal libraries (df = 2, t-critical = 4.303) for error characterisation in variant-free datasets (Delta, wild-type, and Omicron). Subsampling replicates represent computational draws rather than independent library preparations; reported CIs therefore reflect depth-normalised sampling variability. Statistical significance was set at α = 0.05. Statistical analyses including t-tests, linear regression, and ANOVA were conducted using GraphPad Prism (v8.0.1), which was also used for data visualization. Cohen’s d effect sizes were calculated using the ‘effsize’ package in R (v4.2.0). Statistical test results can be found in the study repository.

## Results

LoFreq detected 759 total variant calls across all barcodes, achieving a recall of 84.33% (141 variants missed out of 900 expected). Longshot identified 211 calls with a recall of 23.44% (689 variants missed), while Clair3 made 305 calls with a recall of 33.89% (595 variants missed). LoFreq demonstrated superior sensitivity across the tested allele frequency range, particularly at sub-consensus frequencies (<20%), recovering variants that both Longshot and Clair3 failed to detect. It should be noted that Longshot and Clair3 were not designed for sub-consensus variant identification, especially in haploid in genomes. Hence poor recall was not necessarily indicative of variant caller fitness. Consequently, the remainder of the results focuses exclusively on the results of LoFreq. Detailed variant identification counts can be found in S1 File.

### Sensitivity and false discovery

The use of LoFreq on Nanopore data resulted in false calls across all datasets. To quantify the extent of false discovery between nanopore chemistries, reads were sub-sampled to fixed depths. Within clonal plasmid libraries, which are characterised by a single population of plasmids devoid of variation, LoFreq reported a significant number of variants. Across 12 replicates at a uniform depth of 2,000, we noted averages of 8.25 (95% CI: 7.31–9.19) and 2.00 (95% CI: 1.34–2.66) false calls on R9.4.1 and R10.4.1 flow cells, respectively (unpaired t-test, t = 12.4, df = 22, p < 0.001, Cohen’s d = 4.89) (Fig 1). For mixed plasmid datasets at the same depth, the average false positive calls were 8.67 (95% CI: 7.73–9.61) for R9.4.1 and 4.88 (95% CI: 4.19–5.57) for R10.4.1 (unpaired t-test, t = 6.9, df = 22, p < 0.001, Cohen’s d = 2.93). This contributed an FDR of 12.9% and 6.3% on R9.4.1 and R10.4.1 flow cells respectively (Table 1). Within the genomic datasets containing variants with AF of 10%, the average FDR varied drastically by depth (Fig 2a), with false positive frequency correlating strongly with depth across all chemistries (Pearson’s r = 0.9879). This effect was also observed in plasmid datasets, although to a much smaller extent, presumably due to smaller genome size. On R9.4.1 flow cells, we observed a gradual increase in FDR as depth increased. For R10.4.1, however, we observed a constant decrease in the FDR with every unit of increase in depth, eventually plateauing at a false discovery rate of about 25%. Statistical comparisons between chemistries were performed using unpaired two-sample t-tests, with differences considered significant at p < 0.05, indicating that R10.4.1 improved substantially in sensitivity.

**Table 1:** Recall and false discovery rates for variants at 5%, 10%, 15%, and 19% frequencies across Phred score scalar adjustments. Plasmid truth sets normalised to 2,000 × depth (n = 12 subsampling replicates per condition; df = 11, t-critical = 2.201). The Control row is a technical duplicate of the Uncalibrated condition, confirmed by identical VCF hash signatures. Phred score reduction removes all false positives at −8 on both chemistries. Variants at 15% AF and below are adversely affected by Phred reduction on R9.4.1 flow cells, while only 5% AF variants are affected on R10.4.1 flow cells. CI = 95% confidence interval; FDR = false discovery rate.

**R9.4.1**	Uncalibrated	**39.83**	6.38	**89.45**	1.58	**95.11**	1.47	**99.33**	0.35	**12.39**	0.88
	Control	**39.83**	6.38	**89.45**	1.58	**95.11**	1.47	**99.33**	0.35	**12.39**	0.88
	Minus 1	**36.17**	5.64	**87.55**	2.76	**93.96**	0.99	**99.28**	0.40	**9.31**	0.65
	Minus 2	**34.17**	6.09	**86.71**	3.44	**93.68**	0.85	**99.33**	0.35	**7.34**	0.54
	Minus 3	**31.67**	7.16	**86.39**	3.20	**93.39**	0.63	**99.33**	0.35	**6.63**	0.44
	Minus 4	**27.00**	6.80	**83.97**	3.60	**93.39**	0.63	**99.28**	0.40	**5.86**	0.38
	Minus 5	**22.33**	6.03	**83.02**	3.35	**93.39**	0.63	**99.06**	0.54	**5.40**	0.31
	Minus 6	**17.83**	5.82	**81.75**	3.52	**93.39**	0.63	**99.00**	0.57	**5.41**	0.29
	Minus 7	**13.00**	3.57	**79.33**	3.57	**92.81**	0.63	**98.89**	0.62	**3.61**	0.57
	Minus 8	**10.17**	3.23	**77.53**	3.74	**92.24**	0.99	**98.67**	0.70	**0.50**	0.24
	Minus 9	**7.00**	1.84	**74.16**	3.47	**89.08**	1.83	**98.67**	0.70	**0.47**	0.23
	Minus 10	**5.17**	1.48	**70.36**	3.65	**85.35**	3.25	**98.39**	0.87	**0.43**	0.23
**R10.4.1**	Uncalibrated	**4.89**	1.13	**97.96**	0.00	**100.00**	0.00	**100.00**	0.00	**7.60**	0.58
	Control	**4.89**	1.13	**97.96**	0.00	**100.00**	0.00	**100.00**	0.00	**7.60**	0.58
	Minus 1	**4.03**	0.85	**97.96**	0.00	**100.00**	0.00	**100.00**	0.00	**5.68**	0.56
	Minus 2	**4.03**	0.85	**97.96**	0.00	**100.00**	0.00	**100.00**	0.00	**4.09**	0.47
	Minus 3	**4.03**	0.85	**97.96**	0.00	**100.00**	0.00	**100.00**	0.00	**3.17**	0.42
	Minus 4	**3.74**	0.63	**97.96**	0.00	**100.00**	0.00	**100.00**	0.00	**2.40**	0.32
	Minus 5	**3.45**	0.00	**97.96**	0.00	**100.00**	0.00	**100.00**	0.00	**1.84**	0.29
	Minus 6	**3.45**	0.00	**97.96**	0.00	**100.00**	0.00	**100.00**	0.00	**1.67**	0.25
	Minus 7	**3.45**	0.00	**97.96**	0.00	**100.00**	0.00	**100.00**	0.00	**1.45**	0.12
	Minus 8	**3.45**	0.00	**97.96**	0.00	**100.00**	0.00	**100.00**	0.00	**1.39**	0.00
	Minus 9	**3.45**	0.00	**97.96**	0.00	**100.00**	0.00	**100.00**	0.00	**1.39**	0.00
	Minus 10	**3.45**	0.00	**97.96**	0.00	**100.00**	0.00	**100.00**	0.00	**1.39**	0.00

**Fig 1:**
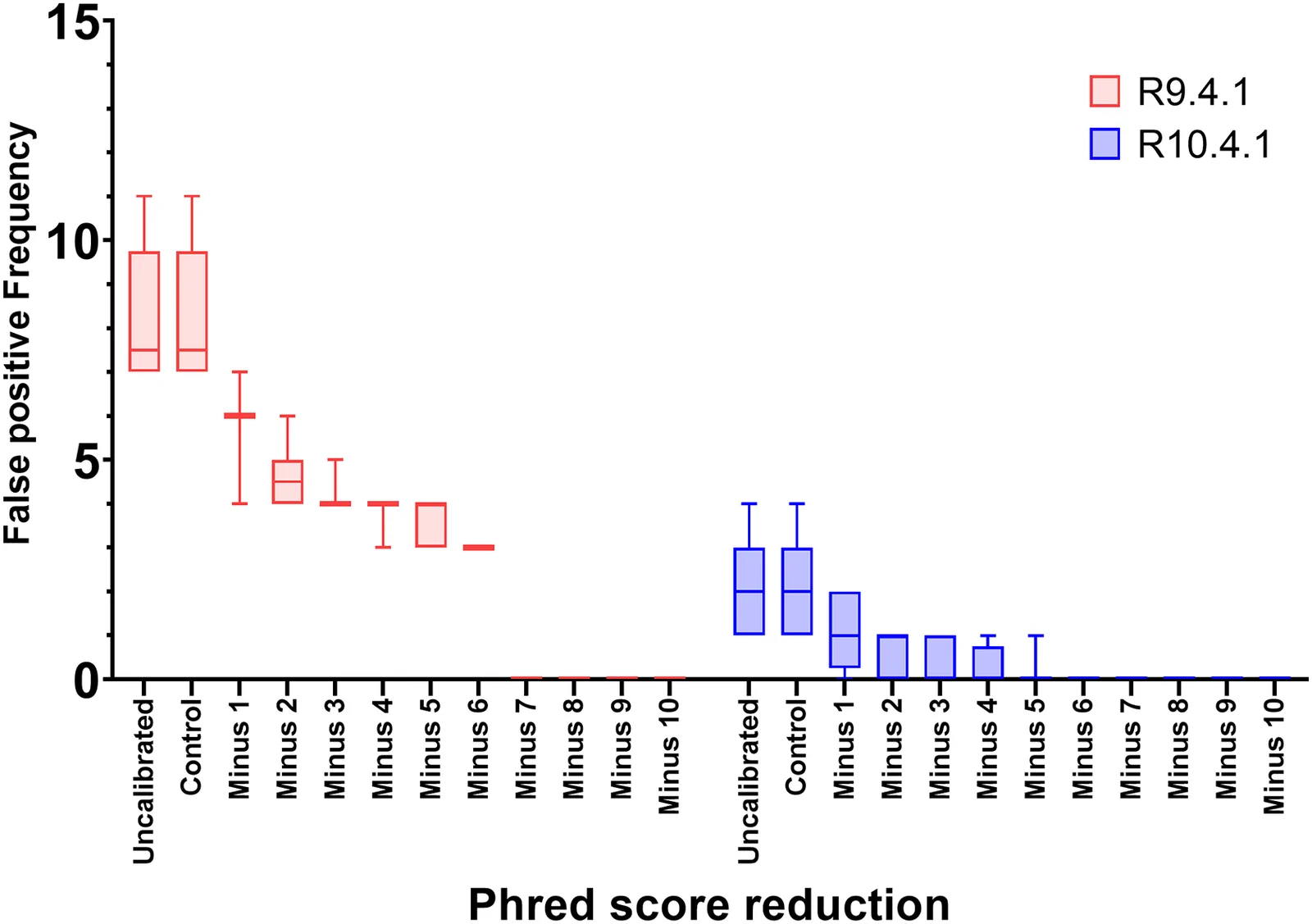
False positive counts in clonal plasmid datasets across Phred score adjustments. False positive distribution for R9.4.1 (left) and R10.4.1 (right) normalised to 2000X depth (n = 12). In clonal plasmid datasets devoid of true variants, progressive Phred score reduction systematically eliminates false positives in both chemistries, with R9.4.1 showing greater improvement from uncalibrated to Minus 5-6, while R10.4.1 demonstrates lower baseline false positive rates.

**Fig 2:**
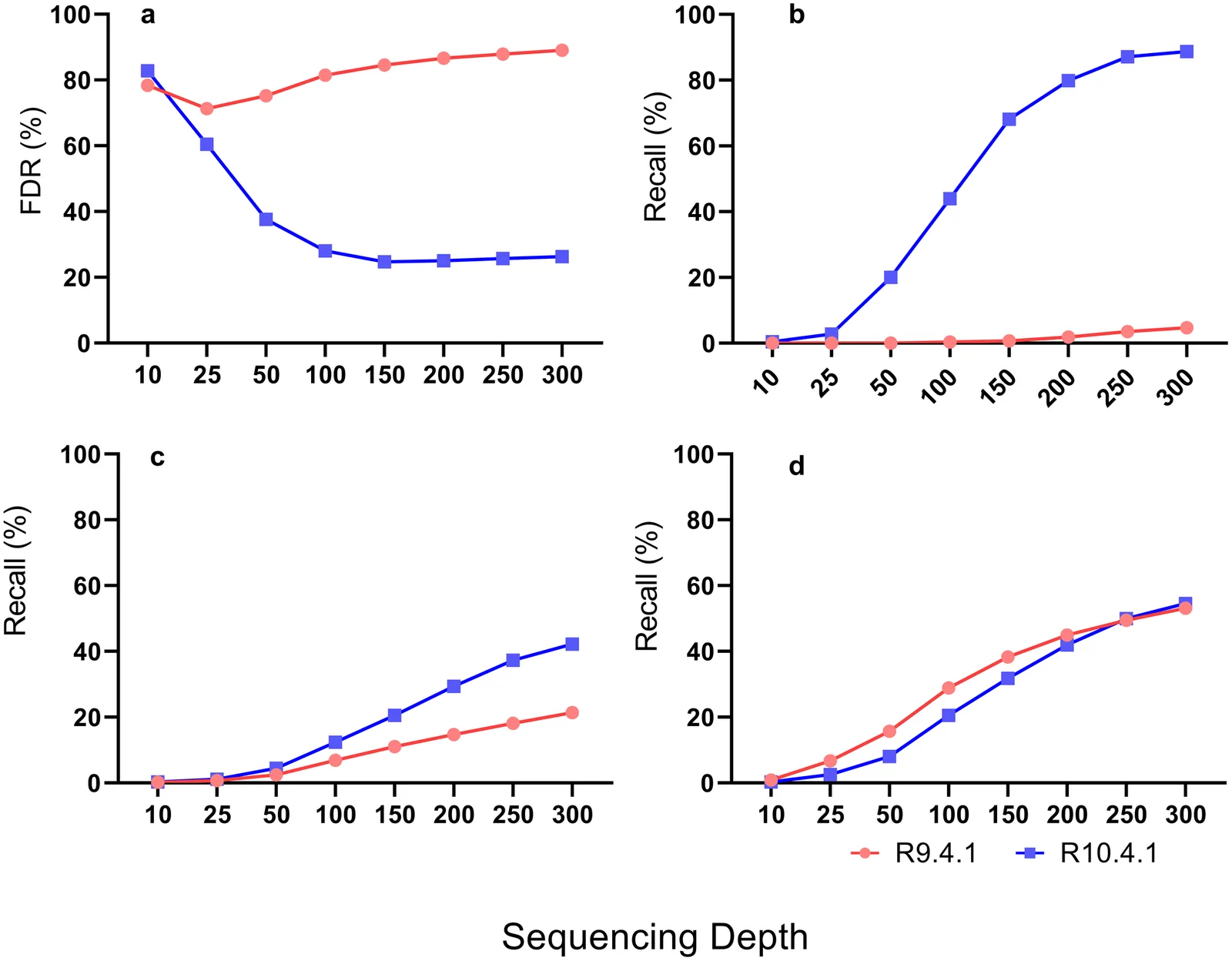
False discovery and recall rate of variants by flow cell chemistry and depth on mixed genomic datasets. Identification of 10% variants by LoFreq with **(a)** False discovery rate as well as recall of **(b)** substitutional **(c)** deletional **(d)** insertional variants at varying depths of 10X to 300X. Recall and FDR was observed to be influenced strongly by depth in both sequencing chemistries.

### Recall per chemistry

Within plasmid datasets, LoFreq detected all variants at frequencies of 10%, 15%, and 19% when using R10.4.1 chemistry, although its recall for variants at allele frequency of 5% was notably low. For R9.4.1 flow cells, the recall rates for 10% and 15% frequency variants were reduced to 90.7% and 95.1%, respectively, while the recall for 5% variants improved to 41.1% (Table 1). In detecting variants with AF of 10% in bacterial genomes, R9.4.1 flow cells showed a low recall rate of less than 5%, whereas R10.4.1 chemistry achieved a high recall rate of 88.7% at identical depths of 300X. Indel discovery was comparable between flow cells, whereas R10.4.1 demonstrated superior recall for insertional variants specifically at higher sequencing depths (Fig 2).

Analysis of homopolymer-associated indels and k-mer error profiles (3-mer through 7-mer) revealed no sequence-context-specific patterns suitable for targeted correction. Homopolymer errors comprised ~35% of total indels with no type bias, and k-mer profile showed diffuse, low-frequency error distributions without clustering. This indicated that sequence-context-dependent approaches to error removal will likely be insufficient. Full methods and results of exploratory homopolymer and K-mer analysis are provided in S2 File. Given the absence of exploitable sequence-specific error patterns, we investigated Phred score encoding within the fastQ files.

### Phred quality score encoding and reduction

Analysis of per-base Phred quality score distribution revealed differences between chemistries. Both chemistries had a high modal score, with R9.4.1 at 31 and R10.4.1 at 38. Notably, R10.4.1 reads exhibited a significant number of bases with a score of 50, which would be the true modal score. However, we focus on the scores within the range of 31–38, as these offered a more robust estimate of central tendency (Fig 3a). Our QvsQ (Fig 3b, 3c) plots showed a slight increase in quality scores starting at Phred scores of 12 for R9.4.1 chemistry, whereas R10.4.1 chemistry began deviating around Phred scores of 20. While R10.4.1 chemistry maintained relatively accurate Phred scores between 40–50, R9.4.1 showed substantial deviation in this range (Fig 3b). Overall, both chemistries exhibited Phred score deviation from expected, with R10.4.1 showing greater accuracy compared to R9.4.1, indicating a need for adjustment.

**Fig 3:**
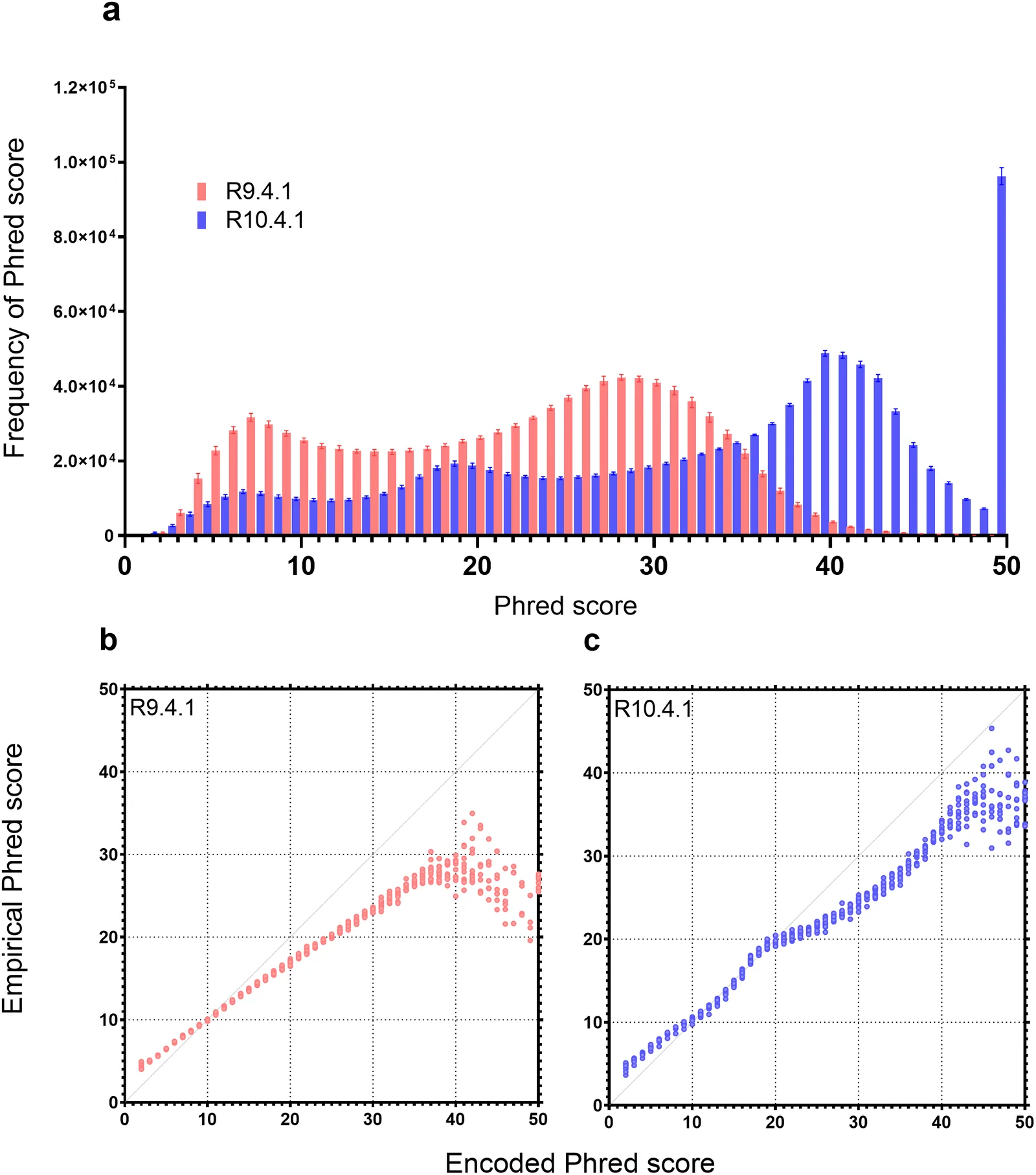
Phred score distribution and representation. (a) Distribution of Phred scores from 1 million randomly sampled bases across subsamples of clonal plasmid datasets (n = 12). R10.4.1 shows a narrower, higher-quality distribution compared to R9.4.1. (b, c) QvQ plots comparing empirical versus encoded Phred scores for R9.4.1 and R10.4.1, respectively. R9.4.1 shows systematic quality score overestimation, while R10.4.1 demonstrates improved representation with minor overestimation.

We explored the impact of fitted calibration as well as scalar reductions in Phred scores on the occurrence of false positives. For this, we focused on the R9.4.1 mixed plasmid dataset to assess potential approaches. Scalar reduction demonstrated the most effective suppression of false positives compared to all fitted calibration methods (Table 2). Consequently, we aimed to determine the magnitude of Phred score reduction necessary to eliminate false positives in the clonal plasmid datasets. Given that these datasets were clonal, any identified variants could be immediately classified as false positives. A quality offset of one led to an immediate reduction in false calls, while a Phred reduction of seven completely prevented false calls on R9.4.1 pores, while R10.4.1 required a smaller Phred reduction of six to eliminate all false positives (Fig 1). In the unsampled clonal R10.4.1 datasets with average depths of 50,000 (not shown), a Phred reduction of 6 failed to eliminate all variants, necessitating a Phred reduction of seven. As the retention of true positives was of higher importance, we applied further scalar reduction to datasets with mixed populations, expanded in the following sections.

**Table 2:** Evaluation of Phred score calibration methods on variant calling accuracy on mixed plasmid datasets (R9.4.1), comparison of scalar reduction versus curve-fitting approaches (quadratic, polynomial, Gaussian) derived from QvQ plots of clonal plasmids. Only scalar methods resulted in precisions of 100% while scalar minus 5 provided optimal balance between precision and recall (F1: 76.2).

Calibration method	False positive counts (#)	Recall (%)			Precision (%)	F1 Score
		10% variants	5% variants	1% variants		
**None (Control)**	41	100	100	3	62	**64.7**
**Quadratic**	19	100	93.9	0	77.1	**70.3**
**Polynomial (3rd degree)**	26	100	97	0	71.4	**68.4**
**Gaussian**	27	100	97	3	71	**68.8**
**Scalar (minus 1)**	14	100	100	0	82.5	**73.7**
**Scalar (minus 3)**	4	100	100	0	94.3	**78.1**
**Scalar (minus 5)**	0	100	84.8	0	100	**76.2**
**Scalar (minus 7)**	0	100	39.4	0	100	**63.5**

### Effect of true calls in mixed plasmid datasets

The impact of scalar Phred reduction on false call elimination and true call retention exhibited a non-linear pattern and varied across different chemistries and variant populations. Despite significant Phred reductions of up to ten, variants with an AF of 19% in R9.4.1 chemistry remained unaffected, while discovery rates for variants at 15% frequency and lower were impacted (Table 1). The cumulative recall loss for 15% frequency variants was about 9.77% with a Phred reduction of ten, demonstrating a non-linear trend, as reductions between −1 and −7 only caused a 0.57% loss. Unfortunately, we observed a larger recall loss as variant frequency decreased, with variants of AF of 10% and 5% losing about 19.1% and 36.7% recall respectively. In contrast, variant discovery for frequencies of 10% and above remained unaffected on R10.4.1 flow cells. A reduction of about 1.44% in 5% variant recall was observed; however, because the baseline variant discovery was low, this led to only a 0.2-fold reduction in total discovery (Table 1). Positions with multiple possible variants (described in our methods) were most susceptible to recall loss.

### Effect of true calls in mixed bacterial genomic datasets

Reducing phred scores up to eight had a minor impact on the recall of substitutional variants, particularly in R10.4.1 chemistry, since the baseline recall for R9.4.1 was less than 5% (Fig 4).

**Fig 4:**
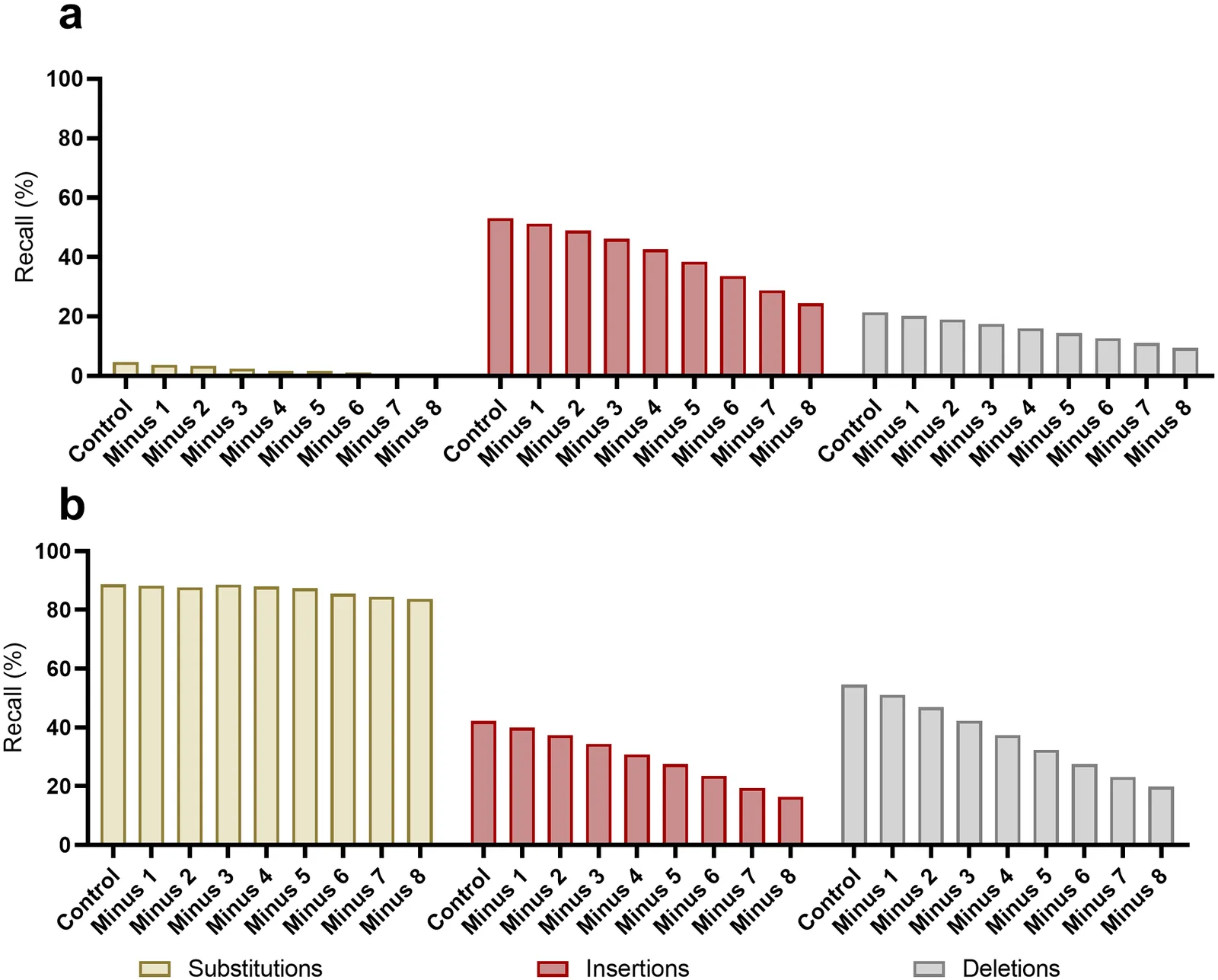
Impact of Phred score reduction on variant recall by mutation type on bacterial genomic datasets. Recall of substitutional (left), insertional (middle), and deletional (right) variants across scalar Phred score reductions (0–10) for (a) R9.4.1 and (b) R10.4.1 flow cells. Both chemistries show rapid decline in indel recall with Phred score reduction, while substitution recall remains more stable.

Conversely, Phred score reductions markedly decreased indel detection in both chemistries. As observed in the plasmid datasets, the reduction in false positives was non-linear, particularly in the R10.4.1 dataset (Fig 5). Regardless, reduction of up to eight in Phred scores led to a 70–75% decrease in false positives across both chemistries. However, some false positives persisted in both chemistries even at the max Phred score reduction of eight. Specifically, Phred reduction of eight significantly decreased false positives in R10.4.1 chemistry (paired t-test, p < 0.001; Cohen’s d = 3.24) and R9.4.1 chemistry (p < 0.001; Cohen’s d = 2.98), indicating very large effect sizes and substantial practical improvement in variant calling specificity across both sequencing chemistries.

**Fig 5:**
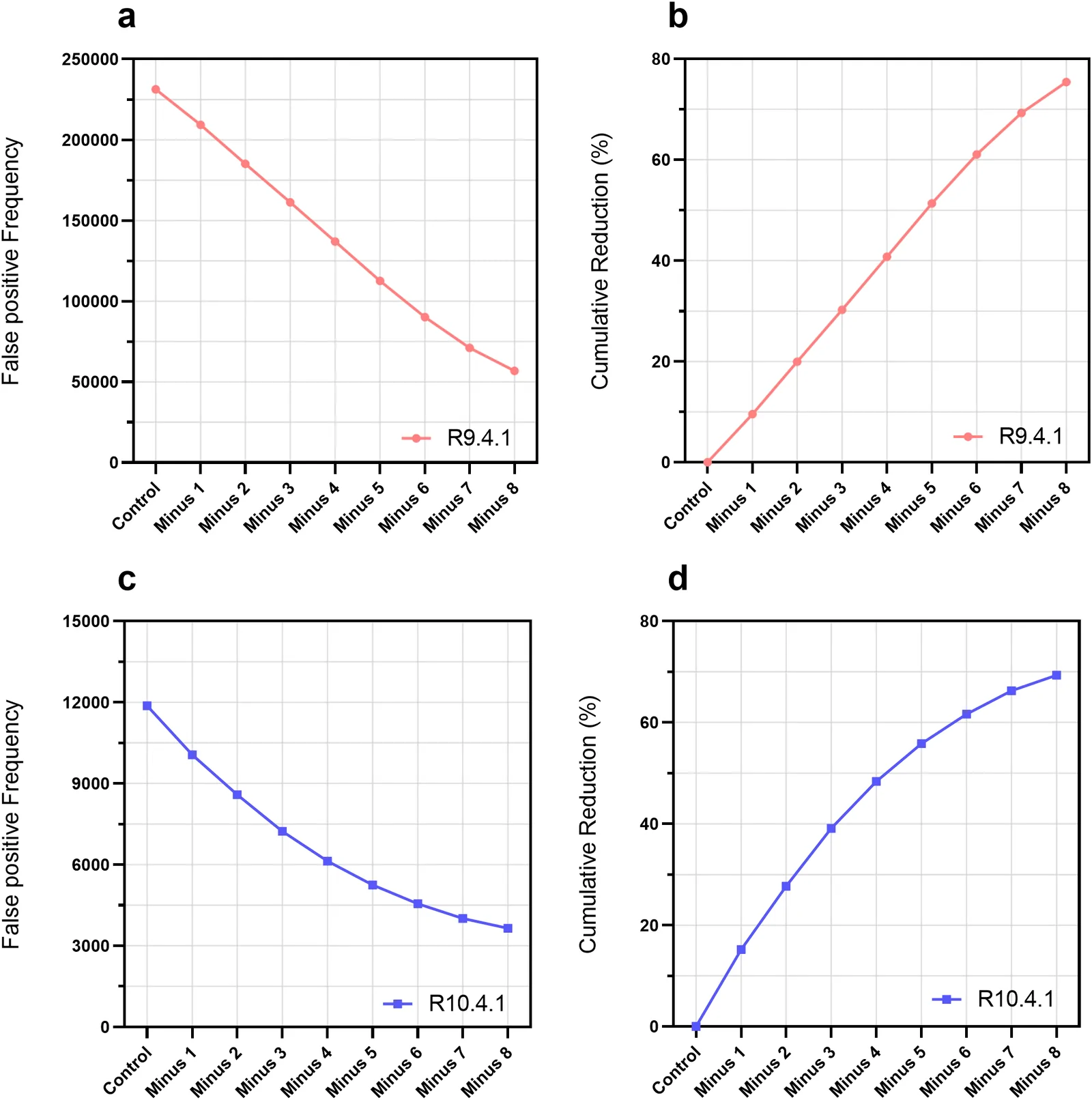
False positive reduction per chemistry with Phred score reductions from minus 1 to 8. **(a)** Frequency of false positives in bacterial genomes sequenced on R9.4.1 flow cells. **(b)** Cumulative (%) reduction in false positives of bacterial genomes sequenced on R9.4.1 flow cells. **(c)** Frequency of false positives in bacterial genomes sequenced on R10.4.1 flow cells. **(d)** Cumulative (%) reduction in false positives of bacterial genomes sequenced on R10.4.1 flow cells.

## Discussion

The analysis of sub-consensus variants in haploid pathogens such as bacteria, fungi, and viruses has a profound impact on our understanding of these organisms [40–42]. In fungal and bacterial pathogens, single genetic events immediately express resistance phenotypes, allowing rare resistant subpopulations to survive drug treatment and complicating infection management [43–45]. These genomic alterations occur rapidly and involve competing subpopulations with distinct genotypes undergoing dynamic population-level fluctuations [46]. Similarly, tracking sub-consensus intra-host viral variants provides crucial insights into population dynamics, transmission patterns, and detection of minority drug-resistant populations within viral quasispecies [33,47]. This diversity informs both vaccine design by identifying conserved epitopes across variant populations [40] and informs policy development for outbreak control [42].In both cases, sub-consensus variants represent emerging resistance mutations that signal treatment failure and evolutionary dynamics before becoming dominant in the population.

Although LoFreq is well-established for Illumina data, accurate sub-consensus variant calling on nanopore reads remains limited. Furthermore, LoFreq’s efficacy on long-read data has not been formally benchmarked, yet it continues to be utilized in practice [32,33,48]. ONT sequencing is particularly appealing for pathogen whole-genome sequencing due to its portability and low cost. This accessibility and cost-effectiveness have facilitated field-based genome generation during novel pathogen outbreaks [49,50]. Furthermore, nanopore technology retains an advantage in haplotype variant studies because of its longer read lengths [8,51].

Longshot and Clair3 were included as contextual reference points; their recall rates in S1 File reflect a fundamental design mismatch rather than overall caller quality. Longshot relies on haplotype-aware phasing to validate variants. When deactivated in haploid contexts, this renders the caller overly conservative, explaining the dramatic reduction in sensitivity we observed. On the other hand, Clair3’s diploid allele frequency assumptions similarly filter variants below ~25% AF as unlikely to be genuine, a threshold incompatible with sub-consensus haploid detection. Dedicated benchmarking of ONT sub-consensus callers remains limited by the scarcity of validated haploid truth sets at defined allele frequencies – a gap this study’s publicly released datasets are intended to help fill.

In this study, the generation of curated truth sets allowed us to assess sensitivity and precision of the short-read variant caller, LoFreq, when applied to long-read technology. This approach offers a distinct advantage over concordance-based methods, such as that of Liu et al., 2022 [33], which relies on cross platform concordance of Illumina and ONT to establish a ground truth. Our methodology provides absolute knowledge of variant presence/absence, precise control over allele frequencies, and elimination of platform-specific biases affecting both technologies. However, it should be noted that our clonally expanded plasmid system does not fully recapitulate clinical viral cDNA libraries derived from RNA templates. While our analysis demonstrated that homopolymer effects and k-mer patterns did not contribute substantially to error rates (see Results), other factors such as strand bias, systematic basecaller errors, and RT-PCR-introduced artifacts may play roles in clinical samples that were not captured in our DNA-based system. These additional complexities should be considered when translating this approach to RNA virus analysis [52]

Our findings demonstrate that applying LoFreq to long-read technology is feasible with strong recall rates for sub-consensus variants. However, high false discovery rates render it unsuitable for immediate use, necessitating further calibration. This result was also noted by prior studies that brought about Variabel, which we discussed earlier. However, Variabel relies on cross sample variant agreement to successfully filter false positive, which requires multiple samples of the same organism. More importantly, this concordance method will naturally lead to misclassification of unique variants as errors. Our study addresses the root cause of elevated FDR by demonstrating that Phred score calibration can substantially reduce false positives. By default, LoFreq employs base alignment quality (BAQ) to enhance the specificity of variant calling. This process is critical, particularly in datasets with higher error rates. Unfortunately, BAQ calculations are inherently slow and optimized for Illumina reads, thus rendering them unsuitable for longer reads. In practice, a single 4 Mb reference alignment at 50 × depth failed to complete BAQ computation even after one week of continuous runtime on 70 threads, precluding its use in our genomic datasets. We therefore, investigated Phred quality scores adjustments as an alternative to improve specificity.

Adjustments to quality scores in sequencing reads have been a topic of discussion for several decades, with proposals targeting Phred scores within Sanger sequencing reads [53,54]. However, our attempts to calibrate quality scores using conventional polynomial fitting methods yielded inconsistent results, ranging from a minor reduction in false discovery to a detrimental increase in some cases. We believe that the observed increase in false discoveries is due to underfitting, which can artificially inflate Phred scores in certain ranges. Notably, raising quality scores of bases within the critical Phred range of 10–25 led to significant increases in false discoveries as noted in the quadratic fit. In contrast, curves with more accurate alignments that only reduced Phred scores resulted in a net decrease in false positive rates (Table 2). Unfortunately, all curve-based calibrations failed to achieve substantial reductions in false positives, likely because the Phred reductions were too modest.. This was consistent when using the auto-calibration method provided by BBMap: toolkits such as BBMap (BBMap - Bushnell B. - sourceforge.net/projects/bbmap/) offer automatic correction of reported Phred values based on empirical alignment-derived calculations; however, we noted only minor improvements in false positive reduction. These findings are in line with Rivara-Espasandín et al., who concluded that quantizing reads that range from 0–93 into four values of 5, 12, 18, and 24 had very little overall effect on variant identification in human and microbial genomes [55].

In contrast, uniform scalar reduction of Phred scores proved more effective at reducing FDR than any of the tested fits. Progressive reduction of Phred scores led to additional decreases in FDR, resulting in the complete elimination of false positives in some of our datasets. Scalar reduction is therefore our recommended approach for Phred calibration. Most importantly, our findings demonstrate that while Phred score reduction effectively reduces false positives, true positives are largely retained. The recall of variants at allelic frequency of 10% and above in both R10.4.1 and R9.4.1 flow cells chemistries were maintained even at aggressive Phred reduction of up to 8. Unfortunately, variants at 5% or less are more adversely affected by Phred score reduction; thus, making this method unsuitable in identifying variants of that population. Additionally, indel identification is diminished with quality score reduction, making this approach less suitable for applications that rely on accurate indel detection.

For genomes with minimal structural variation, such as viral genomes or mutator bacteria, Phred score reduction can be effective. These trade-offs necessitate application-specific calibration strategies. The optimal Phred reduction magnitude depends on analytical objectives. For applications requiring indel detection, Phred calibration is not recommended. When targeting intermediate-frequency variants (10−20% AF), such as viral quasispecies or subclonal populations, aggressive reductions up to −8 effectively eliminate false positives. For balanced detection of variants at 5% AF, a Phred reduction of −5 achieved the optimal F1 score.

In conclusion, our method offers an alternative approach for utilizing the variant caller LoFreq with nanopore data. Particularly given that most tools in the nanopore space prioritize base calling accuracy or alignment refinement rather than sub-consensus variant detection. We suggest that further exploration of quality score adjustments – considering intermediate approaches between uniform reduction and fitted calibration – may enhance recall of ultra-low frequency variants (<5% AF). We emphasize the critical role that Phred scores play in variant calling pipelines that rely on quality scores for their calculations. Finally, our curated truth set reads have been made publicly available to facilitate benchmarking of other variant callers against known allele frequencies in long-read data. However, until a dedicated sub-consensus variant caller for nanopore sequencing becomes available, we provide here within a simple workaround to identifying sub-consensus variants in nanopore long reads.

## Supporting information

S1 File Performance comparison of variant calling tools on SARS-CoV-2 Spike plasmid truth set. This supplementary sheet presents comprehensive validation data for three variant calling tools (Clair3, LoFreq, and Longshot) tested on a synthetic SARS-CoV-2 Spike plasmid truth set containing 100 known variants at positions 2556–2784. Dataset consists of 12 barcoded samples (BC01-BC12) with defined mixtures of Omicron, Delta, and Wild-type containing spike plasmids at varying allele frequencies (1%, 5%, 10%, 15%, 19%, 80%, and 100%). LoFreq achieved the highest sensitivity (759/900 variants called) but with lower precision (1 false call per 3.99 true calls), while Clair3 showed the highest precision (1 false call per 152.5 true calls) but lower sensitivity (305/900 calls). Longshot demonstrated intermediate performance with 211 total calls. Sheet includes detailed barcode statistics, individual variant-level data with expected versus observed allele frequencies, percentage similarity calculations, and performance ratings for each tool across all test conditions.
(PDF)

S2 File Sequence-Context Error Analysis. This supplementary sheet presents sequence specific-context analysis on clonal bacterial genomes; focusing on two key error sources: homopolymer-associated indels and k-mer composition patterns. Homopolymer Analysis: Using Qualimap (v2.2.2-dev) on clonal E. coli K-12 genomes sequenced with R10.4.1 flow cells, indel errors were classified as occurring within homopolymeric regions (≥3 consecutive identical nucleotides: polyA, polyC, polyG, or polyT) versus non-homopolymeric regions. K-mer Analysis: K-mer signatures (3-mer through 7-mer) were computed using sourmash (v4.6.1) with singleton flag to characterize sequence complexity patterns. For each k-mer length, frequency distributions and correlation matrices were generated to assess sequencing consistency across samples.
(PDF)

## Abbreviations

AF: Allele frequency
CI: Confidence interval
BAQ: Base alignment quality
FDR: False discovery rate
NGS: Next Generation Sequencing
ONT: Oxford Nanopore Technologies
PCR: Polymerase Chain Reaction
QvsQ: Empirical Phred vs. encoded Phred scores
SNV: Single nucleotide variant
SUP: Super accurate
VCF: Variant call file
WGS: Whole Genome Sequencing
