# Supplementary material for: Sub-consensus haploid variant calling in long-read sequencing technology
Source: PLoS One. 2026 Oct 5;21(10):e0356259. doi: 10.1371/journal.pone.0356259 (PMC13638048; doi:10.1371/journal.pone.0356259)
Supplement: Supplementary file 1 [file pone.0356259.s001.pdf]

SARS-CoV2 Spike plasmid truth set: VCF report I

Plasmid size: 7179 bp

Expected variants: 100, between positions 2556 - 2784
(Full information under 'Expected Alternate Frequency' list, page 4)

Mix ratio information:

	Omicron	Delta	Wild-type
Barcode 1	1	0	0
Barcode 2	0	1	0
Barcode 3	0	0	1
Barcode 4	0.8	0.1	0.1
Barcode 5	0.8	0.15	0.05
Barcode 6	0.8	0.19	0.01
Barcode 7	0.1	0.8	0.1
Barcode 8	0.15	0.8	0.05
Barcode 9	0.19	0.8	0.01
Barcode 10	0.1	0.1	0.8
Barcode 11	0.05	0.15	0.8
Barcode 12	0.01	0.19	0.8

Alignment: minimap2 v2.24
Reference: Delta for all barcodes
Parameters: `-ax map-ont`

Variant caller parameters:

Clair3: `--guppy5supmodel --haploid_sensitive --include_all_ctgs --no_phasing_for_fa --call_snp_only`

LoFreq: `call-parallel --pp-threads 8 -B`

Longshot: `-n` (default parameters)

Overall performance across 9 mixes (barcodes04-12):

Variant calls with:	Clair		LoFreq		Longshot	
AF within 5% of expectation (95%- 105%)	167	305	241	759	110	211
AF within 25% of expectation (75-125%)	118		358		24	
AF > 25% off expection	20		160		77	
Missed variants	595		141		689	
Total	900		900		900	

*AF = Allele Frequency

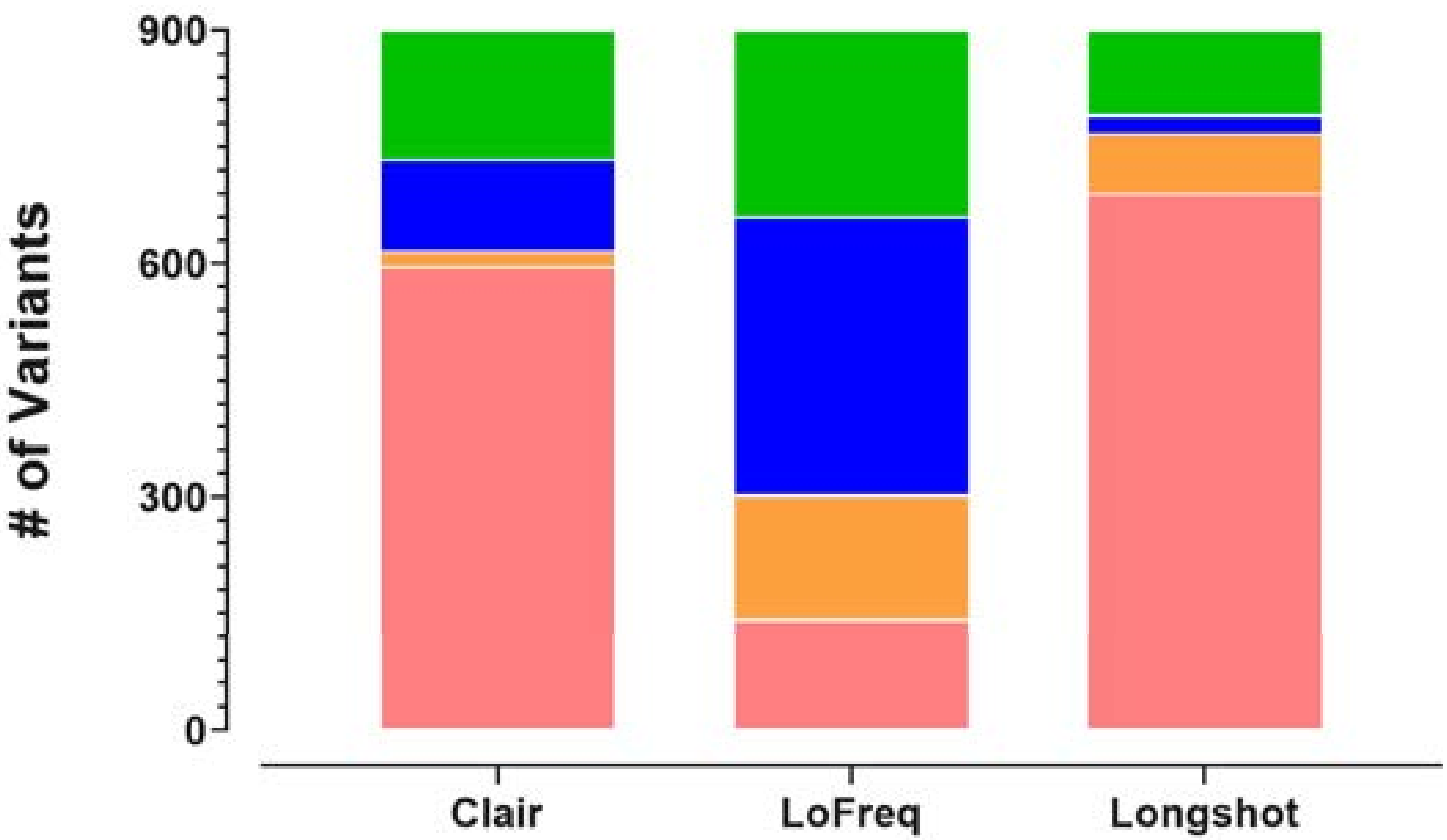

Individual Barcode statistics:

Clair	Raw Calls	Af within 5% of expectation (95%-105%)	Af within 25% of expectation (75-125%)	Called but > 25% off prediction	Variants missed	False calls
BC04	41	22	18	1	59	1
BC05	40	20	19	1	60	0
BC06	46	29	16	1	54	1
BC07	2	0	1	1	98	0
BC08	2	0	0	2	98	0
BC09	10	0	1	9	90	0
BC10	59	34	23	2	41	0
BC11	52	32	19	1	48	0
BC12	53	30	21	2	47	0
Total calls (out of 900)	305	167	118	20	595	2
	1 false call for every 152.50 true calls					

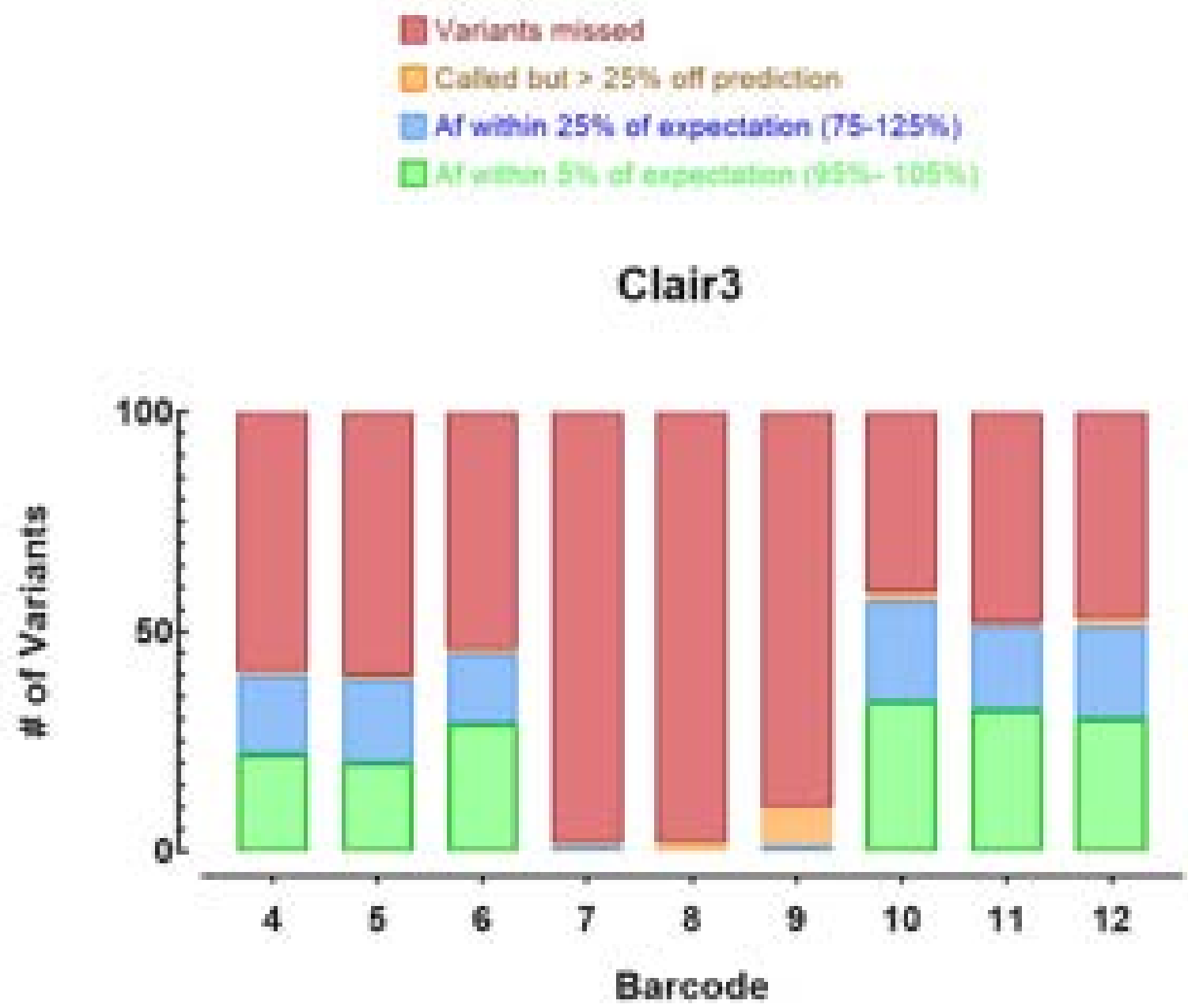

LoFreq	Raw Calls	Af within 5% of expectation (95%-105%)	Af within 25% of expectation (75-125%)	Called but > 25% off prediction	Variants missed	False calls
BC04	100	34	62	4	0	21
BC05	97	39	51	7	3	22
BC06	50	27	23	0	50	25
BC07	100	6	56	38	0	12
BC08	98	3	31	64	2	16
BC09	51	2	13	36	49	14
BC10	100	35	59	6	0	28
BC11	100	53	44	3	0	27
BC12	63	42	19	2	37	25
Total calls (out of 900)	759	241	358	160	141	190
	1 false call for every 3.99 true calls					

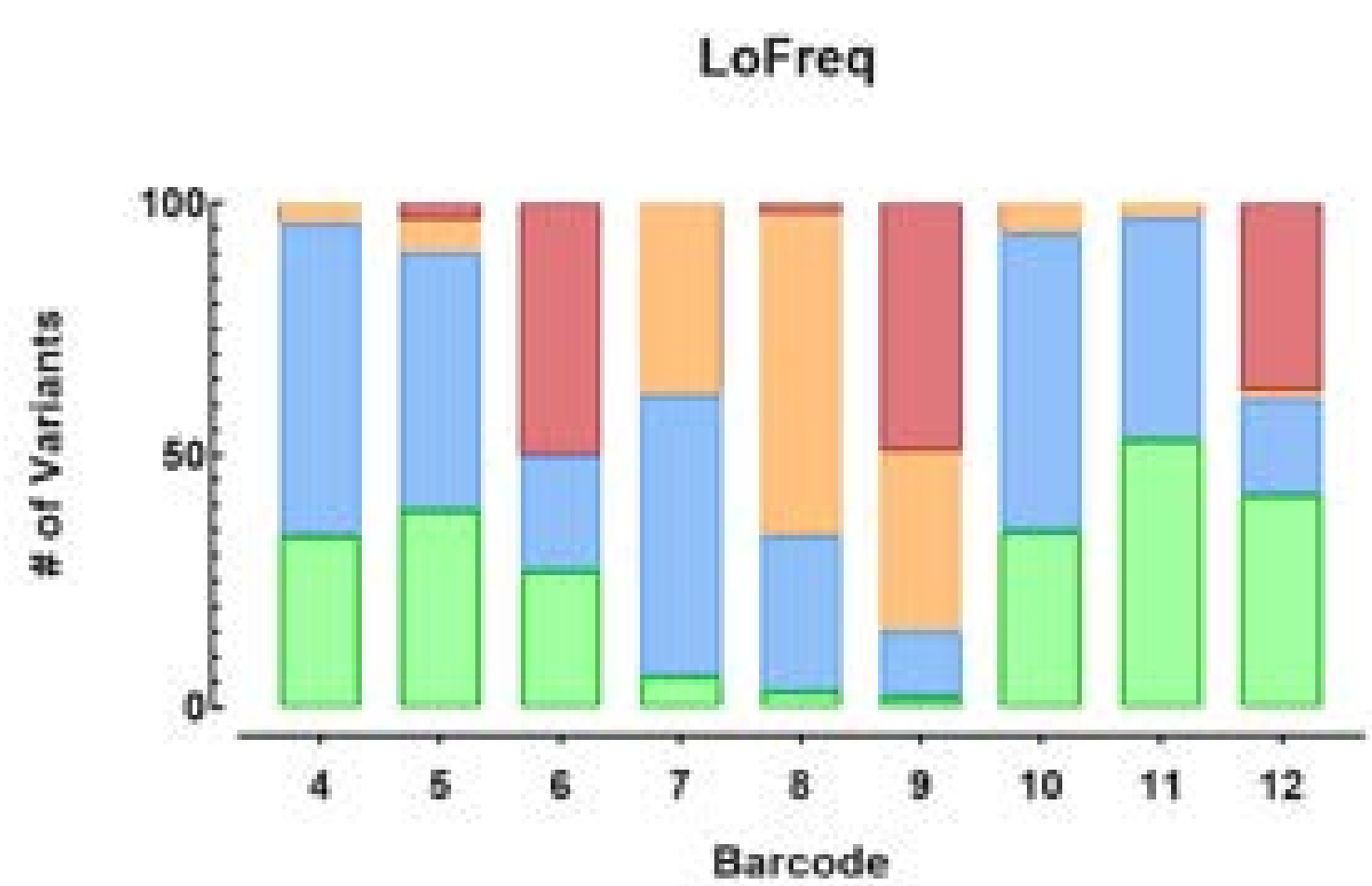

Longshot	Raw Calls	Af within 5% of expectation (95%-105%)	Af within 25% of expectation (75-125%)	Called but > 25% off prediction	Variants missed	False calls
BC04	23	20	3	0	77	1
BC05	21	19	2	0	79	1
BC06	19	17	2	0	81	0
BC07	19	0	5	14	81	0
BC08	18	0	0	18	82	0
BC09	27	0	1	26	73	0
BC10	32	26	6	0	68	0
BC11	22	0	3	19	78	0
BC12	30	28	2	0	70	1
Total calls (out of 900)	211	110	24	77	689	3
	1 false call for every 70.33 true calls					

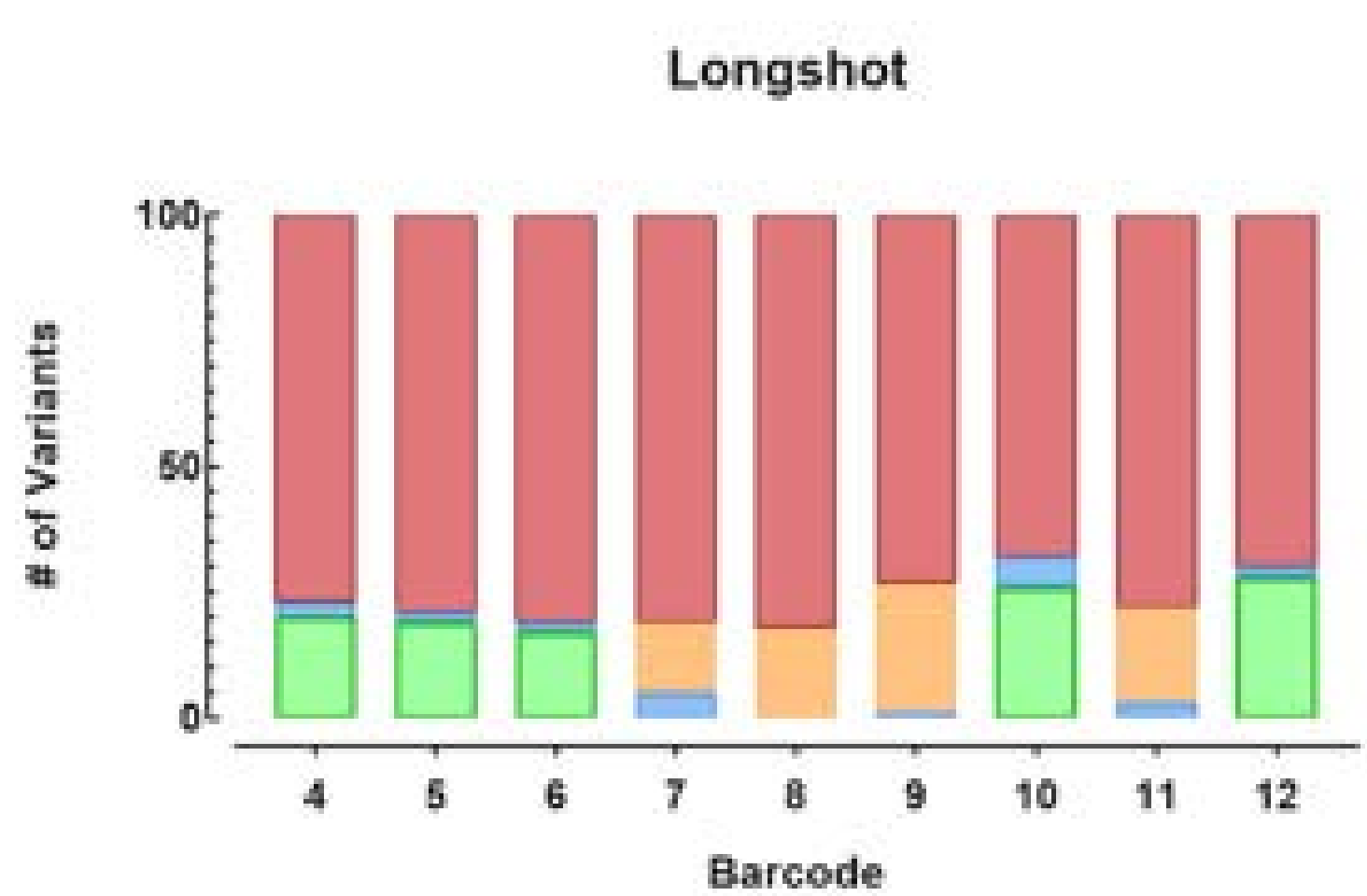

			Expected Alternate Frequency							
Pos	Ref	Alt	BC04	BC05	BC06	BC07	BC08	BC09	BC10	BC11	BC12
2556	C	T	0.903046	0.854603	0.815865	0.202797	0.204567	0.205977	0.900016	0.849724	0.809346
2559	A	C	0.806556	0.806369	0.80622	0.103624	0.15509	0.1961	0.103971	0.052089	0.010435
2562	C	T	0.806556	0.806369	0.80622	0.103624	0.15509	0.1961	0.103971	0.052089	0.010435
2562	C	A	0.09649	0.048234	0.009645	0.099174	0.049477	0.009878	0.796045	0.797635	0.798912
2565	T	C	0.806556	0.806369	0.80622	0.103624	0.15509	0.1961	0.103971	0.052089	0.010435
2566	A	T	0.09649	0.048234	0.009645	0.099174	0.049477	0.009878	0.796045	0.797635	0.798912
2567	G	C	0.09649	0.048234	0.009645	0.099174	0.049477	0.009878	0.796045	0.797635	0.798912
2568	T	C	0.806556	0.806369	0.80622	0.103624	0.15509	0.1961	0.103971	0.052089	0.010435
2571	C	T	0.903046	0.854603	0.815865	0.202797	0.204567	0.205977	0.900016	0.849724	0.809346
2574	C	T	0.09649	0.048234	0.009645	0.099174	0.049477	0.009878	0.796045	0.797635	0.798912
2577	A	C	0.806556	0.806369	0.80622	0.103624	0.15509	0.1961	0.103971	0.052089	0.010435
2577	A	T	0.09649	0.048234	0.009645	0.099174	0.049477	0.009878	0.796045	0.797635	0.798912
2580	G	C	0.903046	0.854603	0.815865	0.202797	0.204567	0.205977	0.900016	0.849724	0.809346
2583	C	T	0.09649	0.048234	0.009645	0.099174	0.049477	0.009878	0.796045	0.797635	0.798912
2586	G	C	0.806556	0.806369	0.80622	0.103624	0.15509	0.1961	0.103971	0.052089	0.010435
2586	G	T	0.09649	0.048234	0.009645	0.099174	0.049477	0.009878	0.796045	0.797635	0.798912
2589	T	C	0.806556	0.806369	0.80622	0.103624	0.15509	0.1961	0.103971	0.052089	0.010435
2589	T	A	0.09649	0.048234	0.009645	0.099174	0.049477	0.009878	0.796045	0.797635	0.798912
2592	G	A	0.903046	0.854603	0.815865	0.202797	0.204567	0.205977	0.900016	0.849724	0.809346
2595	C	T	0.806556	0.806369	0.80622	0.103624	0.15509	0.1961	0.103971	0.052089	0.010435
2595	C	A	0.09649	0.048234	0.009645	0.099174	0.049477	0.009878	0.796045	0.797635	0.798912
2598	A	C	0.806556	0.806369	0.80622	0.103624	0.15509	0.1961	0.103971	0.052089	0.010435
2601	C	A	0.903046	0.854603	0.815865	0.202797	0.204567	0.205977	0.900016	0.849724	0.809346
2604	T	C	0.806556	0.806369	0.80622	0.103624	0.15509	0.1961	0.103971	0.052089	0.010435
2607	C	T	0.09649	0.048234	0.009645	0.099174	0.049477	0.009878	0.796045	0.797635	0.798912
2608	A	T	0.90351	0.951766	0.990355	0.900826	0.950523	0.990122	0.203955	0.202365	0.201088
2609	G	C	0.90351	0.951766	0.990355	0.900826	0.950523	0.990122	0.203955	0.202365	0.201088
2610	C	T	0.09649	0.048234	0.009645	0.099174	0.049477	0.009878	0.796045	0.797635	0.798912
2613	T	C	0.09649	0.048234	0.009645	0.099174	0.049477	0.009878	0.796045	0.797635	0.798912
2619	T	G	0.806556	0.806369	0.80622	0.103624	0.15509	0.1961	0.103971	0.052089	0.010435
2622	C	T	0.09649	0.048234	0.009645	0.099174	0.049477	0.009878	0.796045	0.797635	0.798912
2625	C	G	0.806556	0.806369	0.80622	0.103624	0.15509	0.1961	0.103971	0.052089	0.010435
2625	C	T	0.09649	0.048234	0.009645	0.099174	0.049477	0.009878	0.796045	0.797635	0.798912
2628	G	T	0.09649	0.048234	0.009645	0.099174	0.049477	0.009878	0.796045	0.797635	0.798912
2631	T	C	0.806556	0.806369	0.80622	0.103624	0.15509	0.1961	0.103971	0.052089	0.010435
2636	G	A	0.09649	0.048234	0.009645	0.099174	0.049477	0.009878	0.796045	0.797635	0.798912
2637	C	T	0.09649	0.048234	0.009645	0.099174	0.049477	0.009878	0.796045	0.797635	0.798912
2640	G	T	0.09649	0.048234	0.009645	0.099174	0.049477	0.009878	0.796045	0.797635	0.798912
2643	T	C	0.903046	0.854603	0.815865	0.202797	0.204567	0.205977	0.900016	0.849724	0.809346
2646	C	T	0.806556	0.806369	0.80622	0.103624	0.15509	0.1961	0.103971	0.052089	0.010435
2649	C	A	0.09649	0.048234	0.009645	0.099174	0.049477	0.009878	0.796045	0.797635	0.798912
2652	G	A	0.09649	0.048234	0.009645	0.099174	0.049477	0.009878	0.796045	0.797635	0.798912
2655	A	G	0.806556	0.806369	0.80622	0.103624	0.15509	0.1961	0.103971	0.052089	0.010435
2655	A	C	0.09649	0.048234	0.009645	0.099174	0.049477	0.009878	0.796045	0.797635	0.798912
2661	C	A	0.806556	0.806369	0.80622	0.103624	0.15509	0.1961	0.103971	0.052089	0.010435
2661	C	T	0.09649	0.048234	0.009645	0.099174	0.049477	0.009878	0.796045	0.797635	0.798912
2664	C	T	0.09649	0.048234	0.009645	0.099174	0.049477	0.009878	0.796045	0.797635	0.798912
2667	C	T	0.09649	0.048234	0.009645	0.099174	0.049477	0.009878	0.796045	0.797635	0.798912
2670	C	T	0.09649	0.048234	0.009645	0.099174	0.049477	0.009878	0.796045	0.797635	0.798912

2673	T	C	0.806556	0.806369	0.80622	0.103624	0.15509	0.1961	0.103971	0.052089	0.010435
2673	T	A	0.09649	0.048234	0.009645	0.099174	0.049477	0.009878	0.796045	0.797635	0.798912
2676	C	T	0.09649	0.048234	0.009645	0.099174	0.049477	0.009878	0.796045	0.797635	0.798912
2679	A	G	0.806556	0.806369	0.80622	0.103624	0.15509	0.1961	0.103971	0.052089	0.010435
2682	T	G	0.806556	0.806369	0.80622	0.103624	0.15509	0.1961	0.103971	0.052089	0.010435
2685	A	C	0.806556	0.806369	0.80622	0.103624	0.15509	0.1961	0.103971	0.052089	0.010435
2685	A	T	0.09649	0.048234	0.009645	0.099174	0.049477	0.009878	0.796045	0.797635	0.798912
2688	C	T	0.09649	0.048234	0.009645	0.099174	0.049477	0.009878	0.796045	0.797635	0.798912
2691	A	T	0.09649	0.048234	0.009645	0.099174	0.049477	0.009878	0.796045	0.797635	0.798912
2695	C	A	0.806556	0.806369	0.80622	0.103624	0.15509	0.1961	0.103971	0.052089	0.010435
2697	A	G	0.806556	0.806369	0.80622	0.103624	0.15509	0.1961	0.103971	0.052089	0.010435
2697	A	T	0.09649	0.048234	0.009645	0.099174	0.049477	0.009878	0.796045	0.797635	0.798912
2700	A	G	0.806556	0.806369	0.80622	0.103624	0.15509	0.1961	0.103971	0.052089	0.010435
2700	A	T	0.09649	0.048234	0.009645	0.099174	0.049477	0.009878	0.796045	0.797635	0.798912
2703	T	C	0.806556	0.806369	0.80622	0.103624	0.15509	0.1961	0.103971	0.052089	0.010435
2704	T	A	0.806556	0.806369	0.80622	0.103624	0.15509	0.1961	0.103971	0.052089	0.010435
2705	C	G	0.806556	0.806369	0.80622	0.103624	0.15509	0.1961	0.103971	0.052089	0.010435
2706	C	T	0.09649	0.048234	0.009645	0.099174	0.049477	0.009878	0.796045	0.797635	0.798912
2709	C	A	0.903046	0.854603	0.815865	0.202797	0.204567	0.205977	0.900016	0.849724	0.809346
2712	C	T	0.09649	0.048234	0.009645	0.099174	0.049477	0.009878	0.796045	0.797635	0.798912
2713	T	A	0.806556	0.806369	0.80622	0.103624	0.15509	0.1961	0.103971	0.052089	0.010435
2714	C	G	0.806556	0.806369	0.80622	0.103624	0.15509	0.1961	0.103971	0.052089	0.010435
2715	C	T	0.09649	0.048234	0.009645	0.099174	0.049477	0.009878	0.796045	0.797635	0.798912
2718	C	T	0.09649	0.048234	0.009645	0.099174	0.049477	0.009878	0.796045	0.797635	0.798912
2721	C	T	0.09649	0.048234	0.009645	0.099174	0.049477	0.009878	0.796045	0.797635	0.798912
2721	C	A	0.806556	0.806369	0.80622	0.103624	0.15509	0.1961	0.103971	0.052089	0.010435
2724	T	C	0.806556	0.806369	0.80622	0.103624	0.15509	0.1961	0.103971	0.052089	0.010435
2727	G	A	0.09649	0.048234	0.009645	0.099174	0.049477	0.009878	0.796045	0.797635	0.798912
2730	A	C	0.806556	0.806369	0.80622	0.103624	0.15509	0.1961	0.103971	0.052089	0.010435
2731	C	A	0.806556	0.806369	0.80622	0.103624	0.15509	0.1961	0.103971	0.052089	0.010435
2733	T	A	0.806556	0.806369	0.80622	0.103624	0.15509	0.1961	0.103971	0.052089	0.010435
2736	T	A	0.09649	0.048234	0.009645	0.099174	0.049477	0.009878	0.796045	0.797635	0.798912
2736	T	C	0.806556	0.806369	0.80622	0.103624	0.15509	0.1961	0.103971	0.052089	0.010435
2739	A	C	0.903046	0.854603	0.815865	0.202797	0.204567	0.205977	0.900016	0.849724	0.809346
2743	C	T	0.09649	0.048234	0.009645	0.099174	0.049477	0.009878	0.796045	0.797635	0.798912
2745	G	A	0.09649	0.048234	0.009645	0.099174	0.049477	0.009878	0.796045	0.797635	0.798912
2748	C	A	0.09649	0.048234	0.009645	0.099174	0.049477	0.009878	0.796045	0.797635	0.798912
2751	T	G	0.09649	0.048234	0.009645	0.099174	0.049477	0.009878	0.796045	0.797635	0.798912
2754	A	T	0.903046	0.854603	0.815865	0.202797	0.204567	0.205977	0.900016	0.849724	0.809346
2758	C	T	0.806556	0.806369	0.80622	0.103624	0.15509	0.1961	0.103971	0.052089	0.010435
2760	C	T	0.09649	0.048234	0.009645	0.099174	0.049477	0.009878	0.796045	0.797635	0.798912
2763	T	G	0.806556	0.806369	0.80622	0.103624	0.15509	0.1961	0.103971	0.052089	0.010435
2763	T	C	0.09649	0.048234	0.009645	0.099174	0.049477	0.009878	0.796045	0.797635	0.798912
2766	T	C	0.903046	0.854603	0.815865	0.202797	0.204567	0.205977	0.900016	0.849724	0.809346
2769	T	C	0.09649	0.048234	0.009645	0.099174	0.049477	0.009878	0.796045	0.797635	0.798912
2770	A	T	0.903046	0.854603	0.815865	0.202797	0.204567	0.205977	0.900016	0.849724	0.809346
2771	G	C	0.903046	0.854603	0.815865	0.202797	0.204567	0.205977	0.900016	0.849724	0.809346
2772	C	A	0.09649	0.048234	0.009645	0.099174	0.049477	0.009878	0.796045	0.797635	0.798912
2775	C	T	0.09649	0.048234	0.009645	0.099174	0.049477	0.009878	0.796045	0.797635	0.798912
2781	T	C	0.806556	0.806369	0.80622	0.103624	0.15509	0.1961	0.103971	0.052089	0.010435
2784	T	C	0.903046	0.854603	0.815865	0.202797	0.204567	0.205977	0.900016	0.849724	0.809346

Full Variant call data

Barcodes 04 - 12

Barcode 04															
			Clair		LoFreq		Longshot		Expected AF	% Similarity to expected AF			Rating		
Pos	Ref	Alt	Q	Freq	Q	Freq	Q	Freq		Clair	Lof	Long	Clair	Lof	Long
2556	C	T	18.88	0.8219	49314	0.7632	500	0.874577	0.903046	91.01417	84.51395	96.84739	⯪	⯪	★
2559	A	C	16.08	0.6548	49314	0.597776	-	-	0.806556	81.18465	74.1146	0	⯪	☆	☆
2562	C	T	11.06	0.6707	49314	0.597144	-	-	0.806556	83.156	74.03624	0	⯪	☆	☆
2562	C	A	-	-	49314	0.091045	-	-	0.09649	0	94.35705	0	☆	⯪	☆
2565	T	C	16.09	0.7767	49314	0.756124	-	-	0.806556	96.29829	93.7472	0	★	⯪	☆
2566	A	T	-	-	6201	0.088158	-	-	0.09649	0	91.36503	0	☆	⯪	☆
2567	G	C	-	-	6593	0.096886	-	-	0.09649	0	100.4105	0	☆	★	☆
2568	T	C	13.08	0.7975	49314	0.751052	-	-	0.806556	98.87716	93.11835	0	★	⯪	☆
2571	C	T	11.45	0.8649	49314	0.835298	-	-	0.903046	95.77583	92.49781	0	★	⯪	☆
2574	C	T	-	-	7960	0.091868	-	-	0.09649	0	95.20999	0	☆	★	☆
2577	A	C	15.49	0.8067	49314	0.773906	-	-	0.806556	100.0178	95.95188	0	★	★	☆
2577	A	T	-	-	49314	0.092809	-	-	0.09649	0	96.18522	0	☆	★	☆
2580	G	C	14.92	0.8867	49314	0.851351	500	0.876164	0.903046	98.18988	94.27546	97.02322	★	⯪	★
2583	C	T	-	-	3209	0.093661	-	-	0.09649	0	97.06822	0	☆	★	☆
2586	G	C	16.3	0.802	49314	0.770376	500	0.80421	0.806556	99.43508	95.51422	99.70905	★	★	★
2586	G	T	-	-	49314	0.094401	-	-	0.09649	0	97.83514	0	☆	★	☆
2589	T	C	18.92	0.6729	49314	0.699167	500	0.791884	0.806556	83.42876	86.68545	98.1809	⯪	⯪	★
2589	T	A	-	-	49314	0.091644	-	-	0.09649	0	94.97784	0	☆	⯪	☆
2592	G	A	14.42	0.8186	49314	0.803842	-	-	0.903046	90.64874	89.01449	0	⯪	⯪	☆
2595	C	T	7.93	0.699	49314	0.651765	-	-	0.806556	86.66474	80.80836	0	⯪	⯪	☆
2595	C	A	-	-	49314	0.08408	-	-	0.09649	0	87.13868	0	☆	⯪	☆
2598	A	C	15.38	0.769	49314	0.748894	500	0.787215	0.806556	95.34362	92.8508	97.60198	★	⯪	★
2601	C	A	7.75	0.8513	49314	0.845773	500	0.869188	0.903046	94.26981	93.65777	96.25062	⯪	⯪	★
2604	T	C	14.31	0.78	49314	0.776047	-	-	0.806556	96.70744	96.21733	0	★	★	☆
2607	C	T	-	-	5902	0.078202	-	-	0.09649	0	81.04685	0	☆	⯪	☆
2608	A	T	-	-	5479	0.081874	-	-	0.90351	0	9.061769	0	☆	☆	☆
2609	G	C	-	-	4739	0.076958	-	-	0.90351	0	8.517669	0	☆	☆	☆
2610	C	T	-	-	7597	0.087492	-	-	0.09649	0	90.6748	0	☆	⯪	☆
2613	T	C	-	-	12614	0.092226	-	-	0.09649	0	95.58102	0	☆	★	☆
2619	T	G	14.78	0.7839	49314	0.757516	500	0.793347	0.806556	97.19098	93.91978	98.36225	★	⯪	★
2622	C	T	-	-	7452	0.100682	-	-	0.09649	0	104.3446	0	☆	★	☆
2625	C	G	18.67	0.7859	49314	0.750625	-	-	0.806556	97.43894	93.06541	0	★	⯪	☆
2625	C	T	-	-	49314	0.091641	-	-	0.09649	0	94.97474	0	☆	⯪	☆
2628	G	T	-	-	13085	0.093106	-	-	0.09649	0	96.49303	0	☆	★	☆
2631	T	C	15.86	0.7089	49314	0.712415	500	0.783241	0.806556	87.89218	88.32799	97.10924	⯪	⯪	★
2636	G	A	-	-	4680	0.098762	-	-	0.09649	0	102.3548	0	☆	★	☆
2637	C	T	-	-	4216	0.097088	-	-	0.09649	0	100.6199	0	☆	★	☆
2640	G	T	-	-	7738	0.092875	-	-	0.09649	0	96.25363	0	☆	★	☆
2643	T	C	16.56	0.8797	49314	0.858566	-	-	0.903046	97.41472	95.07442	0	★	★	☆
2646	C	T	18.35	0.7746	49314	0.76533	500	0.788768	0.806556	96.03793	94.88859	97.79454	★	⯪	★
2649	C	A	-	-	3903	0.082548	-	-	0.09649	0	85.55095	0	☆	⯪	☆
2652	G	A	-	-	5488	0.104607	-	-	0.09649	0	108.4124	0	☆	⯪	☆
2655	A	G	13.37	0.78	49314	0.744478	-	-	0.806556	96.70744	92.30328	0	★	⯪	☆
2655	A	C	-	-	49314	0.087798	-	-	0.09649	0	90.99193	0	☆	⯪	☆
2661	C	A	5.98	0.7588	49314	0.746983	500	0.77791	0.806556	94.07898	92.61386	96.44829	⯪	⯪	★
2661	C	T	-	-	49314	0.092779	-	-	0.09649	0	96.15413	0	☆	★	☆
2664	C	T	-	-	10733	0.102531	-	-	0.09649	0	106.2609	0	☆	⯪	☆
2667	C	T	-	-	13415	0.093064	-	-	0.09649	0	96.4495	0	☆	★	☆
2670	C	T	-	-	4814	0.09392	-	-	0.09649	0	97.33664	0	☆	★	☆
2673	T	C	18.8	0.7685	49314	0.764137	-	-	0.806556	95.28162	94.74068	0	★	⯪	☆
2673	T	A	-	-	49314	0.09255	-	-	0.09649	0	95.9168	0	☆	★	☆
2676	C	T	-	-	10506	0.093434	-	-	0.09649	0	96.83296	0	☆	★	☆
2679	A	G	15.84	0.7703	49314	0.759978	-	-	0.806556	95.50479	94.22503	0	★	⯪	☆
2682	T	G	15.43	0.723	49314	0.733879	500	0.785356	0.806556	89.64036	90.98918	97.37147	⯪	⯪	★
2685	A	C	13.85	0.5883	49314	0.617919	500	0.773322	0.806556	72.93973	76.612	95.87949	☆	⯪	★
2685	A	T	-	-	49314	0.086747	-	-	0.09649	0	89.9027	0	☆	⯪	☆
2688	C	T	-	-	6316	0.096075	-	-	0.09649	0	99.57004	0	☆	★	☆
2691	A	T	-	-	4171	0.088129	-	-	0.09649	0	91.33497	0	☆	⯪	☆
2695	C	A	-	-	49314	0.740408	500	0.781666	0.806556	0	91.79867	96.91401	☆	⯪	★
2697	A	G	-	-	49314	0.691842	-	-	0.806556	0	85.77727	0	☆	⯪	☆
2697	A	T	-	-	49314	0.108375	-	-	0.09649	0	112.3175	0	☆	⯪	☆
2700	A	G	-	-	49314	0.727365	-	-	0.806556	0	90.18155	0	☆	⯪	☆
2700	A	T	-	-	49314	0.095508	-	-	0.09649	0	98.98241	0	☆	★	☆
2703	T	C	3.81	0.6873	49314	0.654005	-	-	0.806556	85.21413	81.08609	0	⯪	⯪	☆
2704	T	A	-	-	49314	0.656628	500	0.708377	0.806556	0	81.4113	87.82728	☆	⯪	⯪
2705	C	G	-	-	49314	0.691748	500	0.751976	0.806556	0	85.76561	93.23292	☆	⯪	⯪

2706	C	T	-	-	6081	0.098159	-	-	0.09649	0	101.7298	0	☆	★	☆
2709	C	A	-	-	49314	0.783622	500	0.844953	0.903046	0	86.7754	93.56693	☆	⯪	⯪
2712	C	T	-	-	5847	0.097449	-	-	0.09649	0	100.994	0	☆	★	☆
2713	T	A	-	-	49314	0.744909	500	0.795649	0.806556	0	92.35672	98.64766	☆	⯪	★
2714	C	G	-	-	49314	0.742538	500	0.776559	0.806556	0	92.06275	96.28076	☆	⯪	★
2715	C	T	-	-	6065	0.094035	-	-	0.09649	0	97.45582	0	☆	★	☆
2718	C	T	-	-	5446	0.094391	-	-	0.09649	0	97.82478	0	☆	★	☆
2721	C	T	-	-	49314	0.084444	-	-	0.09649	0	87.51592	0	☆	⯪	☆
2721	C	A	19.31	0.6667	49314	0.69253	-	-	0.806556	82.66006	85.86257	0	⯪	⯪	☆
2724	T	C	21.16	0.6602	49314	0.707368	-	-	0.806556	81.85417	87.70224	0	⯪	⯪	☆
2727	G	A	-	-	5662	0.093654	-	-	0.09649	0	97.06096	0	☆	★	☆
2730	A	C	14.42	0.7154	49314	0.732074	500	0.7945	0.806556	88.69808	90.76539	98.50516	⯪	⯪	★
2731	C	A	14.73	0.7245	49314	0.7306	-	-	0.806556	89.82633	90.58263	0	⯪	⯪	☆
2733	T	A	14.23	0.7716	49314	0.75787	-	-	0.806556	95.66597	93.96368	0	★	⯪	☆
2736	T	A	-	-	49314	0.090915	-	-	0.09649	0	94.22232	0	☆	⯪	☆
2736	T	C	18.46	0.7629	49314	0.732903	-	-	0.806556	94.58731	90.86817	0	⯪	⯪	☆
2739	A	C	13.35	0.8803	49314	0.840518	-	-	0.903046	97.48117	93.07585	0	★	⯪	☆
2743	C	T	-	-	9341	0.091703	-	-	0.09649	0	95.03899	0	☆	★	☆
2745	G	A	-	-	7473	0.088831	-	-	0.09649	0	92.06251	0	☆	⯪	☆
2748	C	A	-	-	6363	0.08299	-	-	0.09649	0	86.00903	0	☆	⯪	☆
2751	T	G	-	-	2847	0.07499	-	-	0.09649	0	77.718	0	☆	⯪	☆
2754	A	T	5.88	0.8693	49314	0.843617	500	0.870911	0.903046	96.26307	93.41903	96.4415	★	⯪	★
2758	C	T	3.14	0.7748	49314	0.776025	-	-	0.806556	96.06272	96.2146	0	★	★	☆
2760	C	T	-	-	7665	0.095345	-	-	0.09649	0	98.81348	0	☆	★	☆
2763	T	G	7.5	0.7704	49314	0.750165	500	0.794676	0.806556	95.51719	93.00838	98.527	★	⯪	★
2763	T	C	-	-	49314	0.092225	-	-	0.09649	0	95.57998	0	☆	★	☆
2766	T	C	4.26	0.8493	49314	0.790528	-	-	0.903046	94.04834	87.54015	0	⯪	⯪	☆
2769	T	C	-	-	3543	0.087882	-	-	0.09649	0	91.07899	0	☆	⯪	☆
2770	A	T	9.53	0.7896	49314	0.734934	-	-	0.903046	87.43738	81.38387	0	⯪	⯪	☆
2771	G	C	-	-	49314	0.777028	-	-	0.903046	0	86.04521	0	☆	⯪	☆
2772	C	A	-	-	4266	0.074941	-	-	0.09649	0	77.66722	0	☆	⯪	☆
2775	C	T	-	-	7612	0.100878	-	-	0.09649	0	104.5478	0	☆	★	☆
2781	T	C	9.17	0.786	49314	0.768004	500	0.800296	0.806556	97.45134	95.22013	99.22377	★	★	★
2784	T	C	17.15	0.9029	49314	0.890923	500	0.916118	0.903046	99.98381	98.65752	101.4475	★	★	★
Noise/ false calls															
111	A	G			16361	0.255112	500	0.321379							
166	A	G			587	0.090737									
183	A	G			20693	0.277762									
312	G	A			909	0.044267									
313	A	G			1203	0.049167									
425	A	G			71	0.043448									
1212	T	C			72	0.036982									
2558	C	T			2849	0.088207									
2701	T	C			76	0.033476									
2722	T	A			4459	0.070136									
2771	G	T			49314	0.056717									
3567	C	T			187	0.026317									
3597	A	G			204	0.037462									
3665	A	G			89	0.060931									
4066	A	G			515	0.109913									
5213	T	C	10.43	0.2326	21715	0.261644									
5531	C	T			3061	0.103099									
6295	A	G			79	0.053736									
6342	C	G			255	0.043616									
6675	A	G			1905	0.062234									
6994	A	G			69	0.036108									

Barcode 05															
			Clair		LoFreq		Longshot		Expected AF	% Similarity to expected AF			Rating		
Pos	Ref	Alt	Q	Freq	Q	Freq	Q	Freq		Clair	Lof	Long	Clair	Lof	Long
2556	C	T	10.4	0.7988	49314	0.722247	500	0.83966	0.854603	93.47029	84.51257	98.2515	⯨	⯨	★
2559	A	C	7.51	0.6803	49314	0.602112	-	-	0.806369	84.36581	74.66951	0	⯨	☆	☆
2562	C	T	11	0.7123	49314	0.600033	-	-	0.806369	88.33421	74.41168	0	⯨	☆	☆
2562	C	A	-	-	-	-	-	-	0.048234	0	0	0	☆	☆	☆
2565	T	C	9.8	0.7922	49314	0.768645	-	-	0.806369	98.24282	95.32171	0	★	★	☆
2566	A	T	-	-	850	0.043531	-	-	0.048234	0	90.25009	0	☆	⯨	☆
2567	G	C	-	-	1404	0.051965	-	-	0.048234	0	107.7358	0	☆	⯨	☆
2568	T	C	-	-	49314	0.762645	500	0.818251	0.806369	0	94.57763	101.4735	☆	⯨	★
2571	C	T	8.22	0.8414	49314	0.807084	500	0.842867	0.854603	98.45506	94.43963	98.62667	★	⯨	★
2574	C	T	-	-	2221	0.048281	-	-	0.048234	0	100.098	0	☆	★	☆
2577	A	C	8.38	0.824	49314	0.789326	-	-	0.806369	102.1864	97.88641	0	★	★	☆
2577	A	T	-	-	49314	0.04816	-	-	0.048234	0	99.8471	0	☆	★	☆
2580	G	C	11.99	0.8538	49314	0.825776	500	0.850566	0.854603	99.90603	96.62685	99.52763	★	★	★
2583	C	T	-	-	259	0.05254	-	-	0.048234	0	108.9279	0	☆	⯨	☆
2586	G	C	-	-	49314	0.787887	500	0.820986	0.806369	0	97.70796	101.8127	☆	★	★
2586	G	T	-	-	49314	0.049763	-	-	0.048234	0	103.1705	0	☆	★	☆
2589	T	C	7.29	0.696	49314	0.715522	500	0.806645	0.806369	86.31281	88.73378	100.0342	⯨	⯨	★
2589	T	A	-	-	49314	0.046572	-	-	0.048234	0	96.5548	0	☆	★	☆
2592	G	A	6.19	0.7863	49314	0.776624	-	-	0.854603	92.00763	90.8754	0	⯨	⯨	☆
2595	C	T	8.73	0.7122	49314	0.669058	-	-	0.806369	88.32181	82.97166	0	⯨	⯨	☆
2595	C	A	-	-	49314	0.763133	-	-	0.048234	0	1582.156	0	☆	☆	☆
2598	A	C	9.58	0.7844	-	-	500	0.796896	0.806369	97.27553	0	98.82518	★	☆	★
2601	C	A	9.29	0.8071	49314	0.81747	500	0.83859	0.854603	94.4415	95.65493	98.12624	⯨	★	★
2604	T	C	16.19	0.7978	49314	0.789603	-	-	0.806369	98.93729	97.92076	0	★	★	☆
2607	C	T	-	-	1467	0.040218	-	-	0.048234	0	83.38145	0	☆	⯨	☆
2608	A	T	-	-	1001	0.041683	-	-	0.951766	0	4.379542	0	☆	☆	☆
2609	G	C	-	-	693	0.039098	-	-	0.951766	0	4.107941	0	☆	☆	☆
2610	C	T	-	-	1965	0.045487	-	-	0.048234	0	94.30534	0	☆	⯨	☆
2613	T	C	-	-	5433	0.048861	-	-	0.048234	0	101.3004	0	☆	★	☆
2619	T	G	7.37	0.8007	49314	0.769923	500	0.80534	0.806369	99.29693	95.48019	99.8723	★	★	★
2622	C	T	-	-	2326	0.057309	-	-	0.048234	0	118.8151	0	☆	⯨	☆
2625	C	G	17.7	0.8047	49314	0.764297	-	-	0.806369	99.79298	94.7825	0	★	⯨	☆
2625	C	T	-	-	49314	0.046569	-	-	0.048234	0	96.54858	0	☆	★	☆
2628	G	T	-	-	4337	0.046528	-	-	0.048234	0	96.46358	0	☆	★	☆
2631	T	C	9.35	0.7086	49314	0.726932	500	0.795534	0.806369	87.87537	90.14877	98.65632	⯨	⯨	★
2636	G	A	-	-	471	0.051865	-	-	0.048234	0	107.5284	0	☆	⯨	☆
2637	C	T	-	-	226	0.049615	-	-	0.048234	0	102.8637	0	☆	★	☆
2640	G	T	-	-	1803	0.046542	-	-	0.048234	0	96.4926	0	☆	★	☆
2643	T	C	7.51	0.845	49314	0.829151	-	-	0.854603	98.87631	97.02177	0	★	★	☆
2646	C	T	12.45	0.7743	49314	0.779231	-	-	0.806369	96.023	96.6345	0	★	★	☆
2649	C	A	-	-	275	0.042173	-	-	0.048234	0	87.43463	0	☆	⯨	☆
2652	G	A	-	-	1261	0.061799	-	-	0.048234	0	128.124	0	☆	☆	☆
2655	A	G	18.57	0.7896	49314	0.759312	-	-	0.806369	97.92039	94.1643	0	★	⯨	☆
2655	A	C	-	-	-	-	-	-	0.048234	0	0	0	☆	☆	☆
2661	C	A	14.52	0.7652	49314	0.760662	500	0.789427	0.806369	94.89448	94.33171	97.89896	⯨	⯨	★
2661	C	T	-	-	49314	0.047061	-	-	0.048234	0	97.56861	0	☆	★	☆
2664	C	T	-	-	4463	0.056912	-	-	0.048234	0	117.9921	0	☆	⯨	☆
2667	C	T	-	-	5234	0.046771	-	-	0.048234	0	96.96738	0	☆	★	☆
2670	C	T	-	-	527	0.048356	-	-	0.048234	0	100.2535	0	☆	★	☆
2673	T	C	17.01	0.773	49314	0.779616	-	-	0.806369	95.86178	96.68225	0	★	★	☆
2673	T	A	-	-	49314	0.048156	-	-	0.048234	0	99.83881	0	☆	★	☆
2676	C	T	-	-	3652	0.047642	-	-	0.048234	0	98.77317	0	☆	★	☆
2679	A	G	12.99	0.7811	49314	0.777079	-	-	0.806369	96.86628	96.36763	0	★	★	☆
2682	T	G	11.79	0.7444	49314	0.749524	-	-	0.806369	92.31502	92.95046	0	⯨	⯨	☆
2685	A	C	11.66	0.5787	49314	0.633367	500	0.786463	0.806369	71.76612	78.54552	97.53142	⯨	⯨	★
2685	A	T	-	-	49314	0.046024	-	-	0.048234	0	95.41867	0	☆	★	☆
2688	C	T	-	-	1701	0.05316	-	-	0.048234	0	110.2133	0	☆	⯨	☆
2691	A	T	-	-	342	0.044716	-	-	0.048234	0	92.70687	0	☆	⯨	☆
2695	C	A	6.87	0.7587	49314	0.752573	500	0.794257	0.806369	94.0884	93.32857	98.49794	⯨	⯨	★
2697	A	G	8.81	0.6766	49314	0.701456	-	-	0.806369	83.90696	86.98942	0	⯨	⯨	☆
2697	A	T	-	-	49314	0.062236	-	-	0.048234	0	129.03	0	☆	☆	☆
2700	A	G	-	-	49314	0.740873	-	-	0.806369	0	91.87763	0	☆	⯨	☆
2700	A	T	-	-	49314	0.048325	-	-	0.048234	0	100.1892	0	☆	★	☆
2703	T	C	-	-	49314	0.667877	-	-	0.806369	0	82.8252	0	☆	⯨	☆
2704	T	A	-	-	49314	0.670299	500	0.719648	0.806369	0	83.12556	89.2455	☆	⯨	⯨
2705	C	G	-	-	49314	0.705294	500	0.764418	0.806369	0	87.46538	94.79746	☆	⯨	⯨

2706	C	T	-	-	1101	0.053431	-	-	0.048234	0	110.7751	0	☆	⯨	☆
2709	C	A	6.69	0.762	49314	0.750881	-	-	0.854603	89.1642	87.86313	0	⯨	⯨	☆
2712	C	T	-	-	1345	0.056402	-	-	0.048234	0	116.9347	0	☆	⯨	☆
2713	T	A	-	-	49314	0.757986	-	-	0.806369	0	93.99986	0	☆	⯨	☆
2714	C	G	-	-	49314	0.756651	500	0.788355	0.806369	0	93.8343	97.76597	☆	⯨	★
2715	C	T	-	-	1307	0.050744	-	-	0.048234	0	105.2043	0	☆	⯨	☆
2718	C	T	-	-	614	0.047833	-	-	0.048234	0	99.16915	0	☆	★	☆
2721	C	T	-	-	49314	0.04225	-	-	0.048234	0	87.59427	0	☆	⯨	☆
2721	C	A	14.28	0.675	49314	0.704596	-	-	0.806369	83.70854	87.37882	0	⯨	⯨	☆
2724	T	C	12.5	0.6811	49314	0.719836	-	-	0.806369	84.46502	89.26877	0	⯨	⯨	☆
2727	G	A	-	-	789	0.048511	-	-	0.048234	0	100.5748	0	☆	★	☆
2730	A	C	10.51	0.7204	49314	0.745196	500	0.80499	0.806369	89.33872	92.41373	99.82892	⯨	⯨	★
2731	C	A	9.21	0.73	49314	0.743722	-	-	0.806369	90.52924	92.23094	0	⯨	⯨	☆
2733	T	A	8.2	0.7796	49314	0.773677	-	-	0.806369	96.68026	95.94574	0	★	★	☆
2736	T	A	-	-	49314	0.047605	-	-	0.048234	0	98.69646	0	☆	★	☆
2736	T	C	15.21	0.7646	49314	0.74551	-	-	0.806369	94.82007	92.45267	0	⯨	⯨	☆
2739	A	C	10.84	0.8405	49314	0.810467	-	-	0.854603	98.34975	94.83549	0	★	⯨	☆
2743	C	T	-	-	2766	0.048009	-	-	0.048234	0	99.53404	0	☆	★	☆
2745	G	A	-	-	1548	0.045131	-	-	0.048234	0	93.56727	0	☆	⯨	☆
2748	C	A	-	-	1337	0.041391	-	-	0.048234	0	85.81336	0	☆	⯨	☆
2751	T	G	-	-	73	0.037951	-	-	0.048234	0	78.68142	0	☆	⯨	☆
2754	A	T	8.68	0.8234	49314	0.81057	500	0.838892	0.854603	96.34882	94.84754	98.16165	★	⯨	★
2758	C	T	14.43	0.7665	49314	0.789103	-	-	0.806369	95.0557	97.85876	0	★	★	☆
2760	C	T	-	-	1954	0.050272	-	-	0.048234	0	104.2258	0	☆	★	☆
2763	T	G	4.05	0.7771	49314	0.766615	500	0.804411	0.806369	96.37023	95.06996	99.75713	★	★	★
2763	T	C	-	-	49314	0.046812	-	-	0.048234	0	97.05238	0	☆	★	☆
2766	T	C	-	-	49314	0.768719	-	-	0.854603	0	89.95041	0	☆	⯨	☆
2769	T	C	-	-	429	0.049379	-	-	0.048234	0	102.3744	0	☆	★	☆
2770	A	T	2.1	0.766	49314	0.71327	-	-	0.854603	89.63225	83.46214	0	⯨	⯨	☆
2771	G	C	-	-	49314	0.753073	-	-	0.854603	0	88.11962	0	☆	⯨	☆
2772	C	A	-	-	362	0.037126	-	-	0.048234	0	76.971	0	☆	⯨	☆
2775	C	T	-	-	2965	0.059942	-	-	0.048234	0	124.274	0	☆	⯨	☆
2781	T	C	11.44	0.7885	49314	0.782443	500	0.814472	0.806369	97.78398	97.03283	101.0049	★	★	★
2784	T	C	18.51	0.8596	49314	0.85934	500	0.880663	0.854603	100.5847	100.5543	103.0493	★	★	★
Noise/ false calls															
111					27092	0.239904	500	0.307313							
166					967	0.088043									
183					36472	0.26569									
312					1932	0.044979									
313					2491	0.050146									
425					263	0.045608									
1212					188	0.038205									
2558					6382	0.093378									
2701					122	0.033552									
2722					9852	0.073837									
2771					49314	0.057199									
3417					970	0.066135									
3567					328	0.026214									
3597					405	0.037774									
4066					832	0.107766									
4344					98	0.039769									
5213					36526	0.249859									
5531					6212	0.10561									
6295					262	0.056244									
6342					465	0.043722									
6416					106	0.06104									
6994					73	0.033241									

Barcodes 06															
			Clair		LoFreq		Longshot		Expected AF	% Similarity to expected AF			Rating		
Pos	Ref	Alt	Q	Freq	Q	Freq	Q	Freq		Clair	Lof	Long	Clair	Lof	Long
2556	C	T	11.9	0.7773	49314	0.701842	500	0.821177	0.815865	95.27315	86.02431	100.6511	★	⯪	★
2559	A	C	9.61	0.6829	49314	0.616174	-	-	0.80622	84.70395	76.42755	0	⯪	⯪	☆
2562	C	T	7.63	0.7136	49314	0.613225	-	-	0.80622	88.51185	76.06177	0	⯪	⯪	☆
2562	C	A	-	-	-	-	-	-	0.009645	0	0	0	☆	☆	☆
2565	T	C	6.82	0.7887	49314	0.785192	-	-	0.80622	97.82692	97.39181	0	★	★	☆
2566	A	T	-	-	-	-	-	-	0.009645	0	0	0	☆	☆	☆
2567	G	C	-	-	-	-	-	-	0.009645	0	0	0	☆	☆	☆
2568	T	C	9.64	0.8165	49314	0.776992	-	-	0.80622	101.2751	96.37472	0	★	★	☆
2571	C	T	5.32	0.7986	49314	0.792035	500	0.823694	0.815865	97.88387	97.07921	100.9596	★	★	★
2574	C	T	-	-	-	-	-	-	0.009645	0	0	0	☆	☆	☆
2577	A	C	12.76	0.8321	49314	0.802934	-	-	0.80622	103.2101	99.59245	0	★	★	☆
2577	A	T	-	-	-	-	-	-	0.009645	0	0	0	☆	☆	☆
2580	G	C	10.06	0.8318	49314	0.803978	500	0.832499	0.815865	101.9532	98.54305	102.0389	★	★	★
2583	C	T	-	-	-	-	-	-	0.009645	0	0	0	☆	☆	☆
2586	G	C	17.48	0.8241	49314	0.800836	500	0.834792	0.80622	102.2178	99.33222	103.5439	★	★	★
2586	G	T	-	-	-	-	-	-	0.009645	0	0	0	☆	☆	☆
2589	T	C	5.5	0.6936	49314	0.727983	500	0.820888	0.80622	86.03113	90.29585	101.8194	⯪	⯪	★
2589	T	A	-	-	-	-	-	-	0.009645	0	0	0	☆	☆	☆
2592	G	A	7.84	0.7574	49314	0.753018	-	-	0.815865	92.83402	92.29692	0	⯪	⯪	☆
2595	C	T	9.38	0.7282	49314	0.676985	-	-	0.80622	90.32277	83.97028	0	⯪	⯪	☆
2595	C	A	-	-	-	-	-	-	0.009645	0	0	0	☆	☆	☆
2598	A	C	7.13	0.7942	49314	0.775887	500	0.813753	0.80622	98.50912	96.23766	100.9344	★	★	★
2601	C	A	8.58	0.7855	49314	0.794769	-	-	0.815865	96.27822	97.41431	0	★	★	☆
2604	T	C	14.17	0.8183	49314	0.806424	-	-	0.80622	101.4984	100.0253	0	★	★	☆
2607	C	T	-	-	-	-	-	-	0.009645	0	0	0	☆	☆	☆
2608	A	T	-	-	-	-	-	-	0.990355	0	0	0	☆	☆	☆
2609	G	C	-	-	-	-	-	-	0.990355	0	0	0	☆	☆	☆
2610	C	T	-	-	-	-	-	-	0.009645	0	0	0	☆	☆	☆
2613	T	C	-	-	-	-	-	-	0.009645	0	0	0	☆	☆	☆
2619	T	G	15.95	0.8111	49314	0.785577	500	0.821431	0.80622	100.6053	97.43956	101.8868	★	★	★
2622	C	T	-	-	-	-	-	-	0.009645	0	0	0	☆	☆	☆
2625	C	G	7.58	0.822	49314	0.77724	-	-	0.80622	101.9573	96.40548	0	★	★	☆
2625	C	T	-	-	-	-	-	-	0.009645	0	0	0	☆	☆	☆
2628	G	T	-	-	-	-	-	-	0.009645	0	0	0	☆	☆	☆
2631	T	C	22.01	0.7265	49314	0.741027	500	0.810438	0.80622	90.11191	91.91377	100.5232	⯪	⯪	★
2636	G	A	-	-	-	-	-	-	0.009645	0	0	0	☆	☆	☆
2637	C	T	-	-	-	-	-	-	0.009645	0	0	0	☆	☆	☆
2640	G	T	-	-	-	-	-	-	0.009645	0	0	0	☆	☆	☆
2643	T	C	8.55	0.8155	49314	0.807228	-	-	0.815865	99.9553	98.9414	0	★	★	☆
2646	C	T	12.43	0.7919	49314	0.793983	-	-	0.80622	98.22384	98.48221	0	★	★	☆
2649	C	A	-	-	-	-	-	-	0.009645	0	0	0	☆	☆	☆
2652	G	A	-	-	-	-	-	-	0.009645	0	0	0	☆	☆	☆
2655	A	G	26.41	0.796	49314	0.773207	-	-	0.80622	98.73238	95.90524	0	★	★	☆
2655	A	C	-	-	-	-	-	-	0.009645	0	0	0	☆	☆	☆
2661	C	A	14.61	0.7787	49314	0.775213	500	0.805803	0.80622	96.58657	96.15406	99.94835	★	★	★
2661	C	T	-	-	-	-	-	-	0.009645	0	0	0	☆	☆	☆
2664	C	T	-	-	-	-	-	-	0.009645	0	0	0	☆	☆	☆
2667	C	T	-	-	-	-	-	-	0.009645	0	0	0	☆	☆	☆
2670	C	T	-	-	-	-	-	-	0.009645	0	0	0	☆	☆	☆
2673	T	C	17.53	0.8002	49314	0.795331	-	-	0.80622	99.25333	98.64941	0	★	★	☆
2673	T	A	-	-	-	-	-	-	0.009645	0	0	0	☆	☆	☆
2676	C	T	-	-	-	-	-	-	0.009645	0	0	0	☆	☆	☆
2679	A	G	15.21	0.789	49314	0.790024	-	-	0.80622	97.86414	97.99115	0	★	★	☆
2682	T	G	8.52	0.7442	49314	0.761872	-	-	0.80622	92.30734	94.4993	0	⯪	⯪	☆
2685	A	C	12.21	0.6024	49314	0.641005	500	0.798943	0.80622	74.71908	79.50748	99.09744	☆	⯪	★
2685	A	T	-	-	-	-	-	-	0.009645	0	0	0	☆	☆	☆
2688	C	T	-	-	-	-	-	-	0.009645	0	0	0	☆	☆	☆
2691	A	T	-	-	-	-	-	-	0.009645	0	0	0	☆	☆	☆
2695	C	A	11.71	0.7813	49314	0.767427	500	0.809129	0.80622	96.90906	95.18831	100.3609	★	★	★
2697	A	G	8.43	0.702	49314	0.718048	-	-	0.80622	87.07303	89.06356	0	⯪	⯪	☆
2697	A	T	-	-	-	-	-	-	0.009645	0	0	0	☆	☆	☆
2700	A	G	-	-	49314	0.755379	-	-	0.80622	0	93.69393	0	☆	⯪	☆
2700	A	T	-	-	-	-	-	-	0.009645	0	0	0	☆	☆	☆
2703	T	C	9.77	0.7249	49314	0.679018	-	-	0.80622	89.91345	84.22245	0	⯪	⯪	☆
2704	T	A	6.53	0.7067	49314	0.683115	500	0.733003	0.80622	87.656	84.73062	90.91853	⯪	⯪	⯪
2705	C	G	-	-	49314	0.71925	500	0.77766	0.80622	0	89.21265	96.45763	☆	⯪	★

2706	C	T	-	-	-	-	-	-	0.009645	0	0	0	☆	☆	☆
2709	C	A	-	-	49314	0.730967	-	-	0.815865	0	89.59414	0	☆	⯪	☆
2712	C	T	-	-	-	-	-	-	0.009645	0	0	0	☆	☆	☆
2713	T	A	-	-	49314	0.775427	-	-	0.80622	0	96.1806	0	☆	★	☆
2714	C	G	8.4	0.7665	49314	0.77245	500	0.803941	0.80622	95.07333	95.81135	99.71733	★	★	★
2715	C	T	-	-	-	-	-	-	0.009645	0	0	0	☆	☆	☆
2718	C	T	-	-	-	-	-	-	0.009645	0	0	0	☆	☆	☆
2721	C	T	-	-	-	-	-	-	0.009645	0	0	0	☆	☆	☆
2721	C	A	12.72	0.6689	49314	0.723249	-	-	0.80622	82.96745	89.70867	0	⯪	⯪	☆
2724	T	C	14.66	0.6689	49314	0.73827	-	-	0.80622	82.96745	91.57181	0	⯪	⯪	☆
2727	G	A	-	-	-	-	-	-	0.009645	0	0	0	☆	☆	☆
2730	A	C	10.09	0.7244	49314	0.756666	500	0.820485	0.80622	89.85143	93.85357	101.7694	⯪	⯪	★
2731	C	A	6.78	0.7301	49314	0.755262	-	-	0.80622	90.55844	93.67942	0	⯪	⯪	☆
2733	T	A	2.87	0.7873	49314	0.787016	-	-	0.80622	97.65327	97.61805	0	★	★	☆
2736	T	A	-	-	-	-	-	-	0.009645	0	0	0	☆	☆	☆
2736	T	C	14.55	0.7777	49314	0.760245	-	-	0.80622	96.46253	94.29749	0	★	⯪	☆
2739	A	C	10.67	0.8149	49314	0.789905	-	-	0.815865	99.88175	96.81813	0	★	★	☆
2743	C	T	-	-	-	-	-	-	0.009645	0	0	0	☆	☆	☆
2745	G	A	-	-	-	-	-	-	0.009645	0	0	0	☆	☆	☆
2748	C	A	-	-	-	-	-	-	0.009645	0	0	0	☆	☆	☆
2751	T	G	-	-	-	-	-	-	0.009645	0	0	0	☆	☆	☆
2754	A	T	11.54	0.8	49314	0.788174	500	0.818868	0.815865	98.05547	96.60597	100.3681	★	★	★
2758	C	T	11.83	0.7936	49314	0.800641	-	-	0.80622	98.4347	99.30803	0	★	★	☆
2760	C	T	-	-	-	-	-	-	0.009645	0	0	0	☆	☆	☆
2763	T	G	8.25	0.7827	49314	0.77697	500	0.819562	0.80622	97.08271	96.37199	101.6549	★	★	★
2763	T	C	-	-	-	-	-	-	0.009645	0	0	0	☆	☆	☆
2766	T	C	9.79	0.7977	49314	0.733463	-	-	0.815865	97.77356	89.90008	0	★	⯪	☆
2769	T	C	-	-	-	-	-	-	0.009645	0	0	0	☆	☆	☆
2770	A	T	7.59	0.7416	49314	0.686703	-	-	0.815865	90.89742	84.16873	0	⯪	⯪	☆
2771	G	C	6.05	0.7573	49314	0.730653	-	-	0.815865	92.82176	89.55566	0	⯪	⯪	☆
2772	C	A	-	-	-	-	-	-	0.009645	0	0	0	☆	☆	☆
2775	C	T	-	-	-	-	-	-	0.009645	0	0	0	☆	☆	☆
2781	T	C	7.5	0.8024	49314	0.797833	500	0.830192	0.80622	99.52621	98.95974	102.9734	★	★	★
2784	T	C	11.31	0.8306	49314	0.837041	500	0.859983	0.815865	101.8061	102.5956	105.4076	★	★	⯪
Noise/ false calls															
111					37047	0.240148									
166					1153	0.085058									
183					48356	0.262393									
312					2417	0.044111									
313					3196	0.049477									
425					499	0.047372									
932					97	0.045573									
1212					272	0.038609									
2558					8034	0.093105									
2568	T	C	500	0.834941											
2701					208	0.035743									
2722					11134	0.070573									
2771					49314	0.0594									
3417					1374	0.06748									
3567					477	0.026434									
3597					503	0.037361									
4066					927	0.104445									
4344					156	0.039912									
5213					49314	0.247342									
5531					8235	0.104089									
6295					265	0.054745									
6342					460	0.041464									
6413					63	0.046358									
6416					121	0.060038									
6675					4302	0.059141									
6994					141	0.034971									

Barcode 07															
			Clair		LoFreq		Longshot		Expected AF	% Similarity to expected AF			Rating		
Pos	Ref	Alt	Q	Freq	Q	Freq	Q	Freq		Clair	Lof	Long	Clair	Lof	Long
2556	C	T	5.41	0.2489	49314	0.238249	500	0.255762	0.202797	122.7334	117.4814	126.1169			
2559	A	C	-	-	6076	0.108249	-	-	0.103624	0	104.4636	0			
2562	C	T	-	-	7067	0.107576	-	-	0.103624	0	103.8141	0			
2562	C	A	-	-	9382	0.118775	-	-	0.099174	0	119.7648	0			
2565	T	C	-	-	28594	0.149709	-	-	0.103624	0	144.4738	0			
2566	A	T	-	-	16376	0.114635	-	-	0.099174	0	115.5903	0			
2567	G	C	-	-	15653	0.116205	-	-	0.099174	0	117.1734	0			
2568	T	C	-	-	21258	0.139283	-	-	0.103624	0	134.4124	0			
2571	C	T	-	-	49314	0.23591	500	0.257609	0.202797	0	116.328	127.0277			
2574	C	T	-	-	14713	0.120152	-	-	0.099174	0	121.1533	0			
2577	A	C	-	-	13840	0.137305	-	-	0.103624	0	132.5035	0			
2577	A	T	-	-	49314	0.117493	-	-	0.099174	0	118.4721	0			
2580	G	C	5.79	0.2649	47389	0.248854	500	0.250059	0.202797	130.6231	122.7108	123.3051			
2583	C	T	-	-	9289	0.125233	-	-	0.099174	0	126.2766	0			
2586	G	C	-	-	28721	0.139059	-	-	0.103624	0	134.1962	0			
2586	G	T	-	-	49314	0.117385	-	-	0.099174	0	118.3632	0			
2589	T	C	-	-	28678	0.12891	-	-	0.103624	0	124.4021	0			
2589	T	A	-	-	49314	0.117682	-	-	0.099174	0	118.6627	0			
2592	G	A	-	-	49314	0.239336	500	0.246668	0.202797	0	118.0174	121.6329			
2595	C	T	-	-	8044	0.125105	-	-	0.103624	0	120.7302	0			
2595	C	A	-	-	5473	0.110647	-	-	0.099174	0	111.5691	0			
2598	A	C	-	-	26241	0.130724	-	-	0.103624	0	126.1527	0			
2601	C	A	-	-	49314	0.251099	500	0.245365	0.202797	0	123.8178	120.9905			
2604	T	C	-	-	30924	0.143882	-	-	0.103624	0	138.8505	0			
2607	C	T	-	-	15736	0.103292	-	-	0.099174	0	104.1528	0			
2608	A	T	-	-	14135	0.109238	-	-	0.900826	0	12.12642	0			
2609	G	C	-	-	10080	0.10194	-	-	0.900826	0	11.31628	0			
2610	C	T	-	-	16587	0.116067	-	-	0.099174	0	117.0342	0			
2613	T	C	-	-	23667	0.119429	-	-	0.099174	0	120.4242	0			
2619	T	G	-	-	17500	0.133348	500	0.139329	0.103624	0	128.6849	134.4566			
2622	C	T	-	-	28445	0.141757	-	-	0.099174	0	142.9383	0			
2625	C	G	-	-	27436	0.132499	-	-	0.103624	0	127.8656	0			
2625	C	T	-	-	49314	0.122172	-	-	0.099174	0	123.1901	0			
2628	G	T	-	-	28617	0.122982	-	-	0.099174	0	124.0069	0			
2631	T	C	-	-	30516	0.126938	-	-	0.103624	0	122.4991	0			
2636	G	A	-	-	13594	0.124811	-	-	0.099174	0	125.8511	0			
2637	C	T	-	-	13747	0.125118	-	-	0.099174	0	126.1607	0			
2640	G	T	-	-	25940	0.123219	-	-	0.099174	0	124.2458	0			
2643	T	C	-	-	49314	0.259402	500	0.248782	0.202797	0	127.912	122.6751			
2646	C	T	-	-	20995	0.146411	500	0.157579	0.103624	0	141.2911	152.069			
2649	C	A	-	-	5420	0.10843	-	-	0.099174	0	109.3336	0			
2652	G	A	-	-	8763	0.123233	-	-	0.099174	0	124.2599	0			
2655	A	G	-	-	8355	0.141959	-	-	0.103624	0	136.9948	0			
2655	A	C	-	-	49314	0.112397	-	-	0.099174	0	113.3336	0			
2661	C	A	-	-	37469	0.132885	500	0.138917	0.103624	0	128.2381	134.0587			
2661	C	T	-	-	49314	0.12274	-	-	0.099174	0	123.7628	0			
2664	C	T	-	-	34727	0.126841	-	-	0.099174	0	127.898	0			
2667	C	T	-	-	37647	0.121741	-	-	0.099174	0	122.7555	0			
2670	C	T	-	-	13780	0.120716	-	-	0.099174	0	121.722	0			
2673	T	C	-	-	25743	0.149044	-	-	0.103624	0	143.832	0			
2673	T	A	-	-	49314	0.116288	-	-	0.099174	0	117.2571	0			
2676	C	T	-	-	22434	0.123921	-	-	0.099174	0	124.9537	0			
2679	A	G	-	-	24717	0.167862	-	-	0.103624	0	161.992	0			
2682	T	G	-	-	17149	0.130313	500	0.136595	0.103624	0	125.756	131.8187			
2685	A	C	-	-	10738	0.111768	-	-	0.103624	0	107.8595	0			
2685	A	T	-	-	10307	0.110014	-	-	0.099174	0	110.9308	0			
2688	C	T	-	-	13118	0.118995	-	-	0.099174	0	119.9866	0			
2691	A	T	-	-	15152	0.116631	-	-	0.099174	0	117.6029	0			
2695	C	A	-	-	19558	0.130922	-	-	0.103624	0	126.3437	0			
2697	A	G	-	-	13121	0.124341	-	-	0.103624	0	119.9929	0			
2697	A	T	-	-	13307	0.125044	-	-	0.099174	0	126.086	0			
2700	A	G	-	-	15305	0.130258	-	-	0.103624	0	125.703	0			
2700	A	T	-	-	13658	0.124284	-	-	0.099174	0	125.3197	0			
2703	T	C	-	-	19574	0.119286	-	-	0.103624	0	115.1146	0			
2704	T	A	-	-	18696	0.116537	-	-	0.103624	0	112.4618	0			
2705	C	G	-	-	18858	0.12198	-	-	0.103624	0	117.7144	0			

2706	C	T	-	-	14126	0.121428	-	-	0.099174	0	122.4399	0
2709	C	A	-	-	46221	0.238151	500	0.249466	0.202797	0	117.4331	123.0127
2712	C	T	-	-	16022	0.129554	-	-	0.099174	0	130.6336	0
2713	T	A	-	-	18815	0.132178	500	0.143475	0.103624	0	127.5558	138.458
2714	C	G	-	-	23779	0.129388	500	0.138744	0.103624	0	124.8634	133.8918
2715	C	T	-	-	17065	0.122005	-	-	0.099174	0	123.0217	0
2718	C	T	-	-	15360	0.124247	-	-	0.099174	0	125.2824	0
2721	C	T	-	-	20231	0.123753	-	-	0.099174	0	124.7843	0
2721	C	A	-	-	49314	0.113348	500	0.137302	0.103624	0	109.3843	132.5006
2724	T	C	-	-	31553	0.131012	-	-	0.103624	0	126.4306	0
2727	G	A	-	-	23108	0.127469	-	-	0.099174	0	128.5312	0
2730	A	C	-	-	16147	0.128218	-	-	0.103624	0	123.7343	0
2731	C	A	-	-	14670	0.127369	-	-	0.103624	0	122.915	0
2733	T	A	-	-	18212	0.134053	-	-	0.103624	0	129.3653	0
2736	T	A	-	-	49314	0.119271	-	-	0.099174	0	120.2649	0
2736	T	C	-	-	21581	0.139141	-	-	0.103624	0	134.2753	0
2739	A	C	-	-	49314	0.248666	-	-	0.202797	0	122.6181	0
2743	C	T	-	-	20943	0.122642	-	-	0.099174	0	123.664	0
2745	G	A	-	-	15177	0.119196	-	-	0.099174	0	120.1893	0
2748	C	A	-	-	12963	0.111316	-	-	0.099174	0	112.2436	0
2751	T	G	-	-	9046	0.099813	-	-	0.099174	0	100.6448	0
2754	A	T	-	-	49314	0.25364	-	-	0.202797	0	125.0708	0
2758	C	T	-	-	34682	0.141487	500	0.147615	0.103624	0	136.5393	142.4532
2760	C	T	-	-	25067	0.123143	-	-	0.099174	0	124.1692	0
2763	T	G	-	-	30208	0.133514	500	0.143566	0.103624	0	128.8451	138.5453
2763	T	C	-	-	49314	0.119816	-	-	0.099174	0	120.8145	0
2766	T	C	-	-	49314	0.2508	500	0.255268	0.202797	0	123.6703	125.8736
2769	T	C	-	-	14616	0.107053	-	-	0.099174	0	107.9451	0
2770	A	T	-	-	49314	0.212245	-	-	0.202797	0	104.6587	0
2771	G	C	-	-	49314	0.220859	500	0.139257	0.202797	0	108.9063	68.668
2772	C	A	-	-	12164	0.09807	-	-	0.099174	0	98.88725	0
2775	C	T	-	-	17542	0.122177	-	-	0.099174	0	123.1951	0
2781	T	C	-	-	24228	0.145066	-	-	0.103624	0	139.9931	0
2784	T	C	-	-	49314	0.270927	500	0.276855	0.202797	0	133.595	136.5179

Noise/ false calls

111	A	G			1449	0.088143		
183	A	G			2511	0.097931		
312	G	A			1339	0.045164		
313	A	G			1685	0.049719		
425	A	G			132	0.044919		
1212	T	C			253	0.042635		
3567	C	T			220	0.026622		
3597	A	G			259	0.037501		
5213	T	C			1771	0.084526		
5531	C	T			4072	0.10561		
6342	C	G			201	0.041493		
6416	A	G			79	0.048018		

Barcode 08															
			Clair		LoFreq		Longshot		Expected AF	% Similarity to expected AF			Rating		
Pos	Ref	Alt	Q	Freq	Q	Freq	Q	Freq		Clair	Lof	Long	Clair	Lof	Long
2556	C	T	10.68	0.2645	49314	0.243586	500	0.271808	0.204567	129.2974	119.0739	132.8698			
2559	A	C	-	-	30419	0.163201	-	-	0.15509	0	105.2296	0			
2562	C	T	-	-	32044	0.162551	-	-	0.15509	0	104.8105	0			
2562	C	A	-	-	49314	0.061266	-	-	0.049477	0	123.828	0			
2565	T	C	-	-	49314	0.220236	-	-	0.15509	0	142.0049	0			
2566	A	T	-	-	4344	0.059054	-	-	0.049477	0	119.3572	0			
2567	G	C	-	-	4041	0.060623	-	-	0.049477	0	122.5284	0			
2568	T	C	-	-	49314	0.207302	-	-	0.15509	0	133.6653	0			
2571	C	T	-	-	49314	0.257553	-	-	0.204567	0	125.9015	0			
2574	C	T	-	-	4072	0.064052	-	-	0.049477	0	129.4589	0			
2577	A	C	-	-	49314	0.206779	-	-	0.15509	0	133.3281	0			
2577	A	T	-	-	49314	0.061378	-	-	0.049477	0	124.0544	0			
2580	G	C	-	-	49314	0.262899	-	-	0.204567	0	128.5148	0			
2583	C	T	-	-	1920	0.073306	-	-	0.049477	0	148.1627	0			
2586	G	C	-	-	49314	0.208272	500	0.216956	0.15509	0	134.2907	139.8901			
2586	G	T	-	-	49314	0.061544	-	-	0.049477	0	124.3899	0			
2589	T	C	-	-	49314	0.191626	500	0.213873	0.15509	0	123.5576	137.9021			
2589	T	A	-	-	49314	0.060773	-	-	0.049477	0	122.8316	0			
2592	G	A	-	-	49314	0.250211	-	-	0.204567	0	122.3124	0			
2595	C	T	-	-	34611	0.183392	500	0.211377	0.15509	0	118.2485	136.293			
2595	C	A	-	-	-	-	-	-	0.049477	0	0	0			
2598	A	C	-	-	49314	0.199956	500	0.206835	0.15509	0	128.9287	133.3644			
2601	C	A	-	-	49314	0.262109	500	0.263044	0.204567	0	128.1286	128.5859			
2604	T	C	-	-	49314	0.214325	-	-	0.15509	0	138.1936	0			
2607	C	T	-	-	4810	0.053476	-	-	0.049477	0	108.0832	0			
2608	A	T	-	-	3884	0.056301	-	-	0.950523	0	5.923158	0			
2609	G	C	-	-	2014	0.052048	-	-	0.950523	0	5.475721	0			
2610	C	T	-	-	5179	0.062357	-	-	0.049477	0	126.0331	0			
2613	T	C	-	-	8875	0.062545	-	-	0.049477	0	126.4131	0			
2619	T	G	-	-	49314	0.20406	500	0.211745	0.15509	0	131.5749	136.5303			
2622	C	T	-	-	13484	0.082473	-	-	0.049477	0	166.6906	0			
2625	C	G	-	-	49314	0.202644	-	-	0.15509	0	130.6619	0			
2625	C	T	-	-	49314	0.062625	-	-	0.049477	0	126.5748	0			
2628	G	T	-	-	10085	0.063302	-	-	0.049477	0	127.9431	0			
2631	T	C	-	-	49314	0.192837	500	0.213681	0.15509	0	124.3385	137.7781			
2636	G	A	-	-	2768	0.06554	-	-	0.049477	0	132.4664	0			
2637	C	T	-	-	2535	0.064673	-	-	0.049477	0	130.7141	0			
2640	G	T	-	-	8967	0.063039	-	-	0.049477	0	127.4115	0			
2643	T	C	-	-	49314	0.271261	500	0.285218	0.204567	0	132.6025	139.4253			
2646	C	T	-	-	49314	0.21448	-	-	0.15509	0	138.2935	0			
2649	C	A	-	-	201	0.05674	-	-	0.049477	0	114.6803	0			
2652	G	A	-	-	1331	0.069476	-	-	0.049477	0	140.4217	0			
2655	A	G	-	-	36475	0.208082	-	-	0.15509	0	134.1682	0			
2655	A	C	-	-	-	-	-	-	0.049477	0	0	0			
2661	C	A	-	-	49314	0.202298	-	-	0.15509	0	130.4388	0			
2661	C	T	-	-	49314	0.063884	-	-	0.049477	0	129.1194	0			
2664	C	T	-	-	15817	0.070264	-	-	0.049477	0	142.0144	0			
2667	C	T	-	-	16244	0.062638	-	-	0.049477	0	126.601	0			
2670	C	T	-	-	3109	0.063834	-	-	0.049477	0	129.0183	0			
2673	T	C	-	-	49314	0.219594	-	-	0.15509	0	141.591	0			
2673	T	A	-	-	49314	0.061317	-	-	0.049477	0	123.9311	0			
2676	C	T	-	-	8089	0.064504	-	-	0.049477	0	130.3725	0			
2679	A	G	-	-	49314	0.234711	-	-	0.15509	0	151.3382	0			
2682	T	G	-	-	49314	0.198378	-	-	0.15509	0	127.9112	0			
2685	A	C	-	-	46853	0.169086	-	-	0.15509	0	109.0242	0			
2685	A	T	-	-	49314	0.057466	-	-	0.049477	0	116.1476	0			
2688	C	T	-	-	3733	0.06504	-	-	0.049477	0	131.4558	0			
2691	A	T	-	-	3742	0.061017	-	-	0.049477	0	123.3247	0			
2695	C	A	-	-	49314	0.198457	500	0.209583	0.15509	0	127.9622	135.136			
2697	A	G	-	-	49314	0.187518	500	0.202395	0.15509	0	120.9088	130.5016			
2697	A	T	-	-	49314	0.068363	-	-	0.049477	0	138.1721	0			
2700	A	G	-	-	49314	0.196483	-	-	0.15509	0	126.6893	0			
2700	A	T	-	-	49314	0.064539	-	-	0.049477	0	130.4432	0			
2703	T	C	-	-	49314	0.177339	-	-	0.15509	0	114.3456	0			
2704	T	A	-	-	49314	0.176216	-	-	0.15509	0	113.6215	0			
2705	C	G	-	-	49314	0.185401	-	-	0.15509	0	119.5438	0			

2706	C	T	-	-	3674	0.065566	-	-	0.049477	0	132.519	0
2709	C	A	-	-	49314	0.247156	500	0.264187	0.204567	0	120.819	129.1446
2712	C	T	-	-	5355	0.073905	-	-	0.049477	0	149.3734	0
2713	T	A	-	-	49314	0.20021	500	0.216769	0.15509	0	129.0925	139.7697
2714	C	G	-	-	49314	0.198504	500	0.208831	0.15509	0	127.9925	134.6511
2715	C	T	-	-	5356	0.066077	-	-	0.049477	0	133.5518	0
2718	C	T	9.99	0.2294	3747	0.064953	-	-	0.049477	463.6527	131.28	0
2721	C	T	-	-	49314	0.058735	-	-	0.049477	0	118.7125	0
2721	C	A	-	-	49314	0.18703	-	-	0.15509	0	120.5942	0
2724	T	C	-	-	49314	0.195546	-	-	0.15509	0	126.0852	0
2727	G	A	-	-	7311	0.068195	-	-	0.049477	0	137.8326	0
2730	A	C	-	-	49314	0.19707	-	-	0.15509	0	127.0678	0
2731	C	A	-	-	49314	0.195357	500	0.205169	0.15509	0	125.9633	132.2898
2733	T	A	-	-	49314	0.204258	-	-	0.15509	0	131.7026	0
2736	T	A	-	-	49314	0.061675	-	-	0.049477	0	124.6547	0
2736	T	C	-	-	49314	0.206549	-	-	0.15509	0	133.1798	0
2739	A	C	-	-	49314	0.261659	-	-	0.204567	0	127.9087	0
2743	C	T	-	-	6692	0.064581	-	-	0.049477	0	130.5281	0
2745	G	A	-	-	3739	0.061443	-	-	0.049477	0	124.1858	0
2748	C	A	-	-	3107	0.057443	-	-	0.049477	0	116.1011	0
2751	T	G	-	-	1323	0.051183	-	-	0.049477	0	103.4487	0
2754	A	T	-	-	49314	0.265204	-	-	0.204567	0	129.6416	0
2758	C	T	-	-	49314	0.212962	500	0.22145	0.15509	0	137.3148	142.7877
2760	C	T	-	-	9051	0.065835	-	-	0.049477	0	133.0627	0
2763	T	G	-	-	49314	0.20303	500	0.213251	0.15509	0	130.9108	137.5013
2763	T	C	-	-	49314	0.063116	-	-	0.049477	0	127.5671	0
2766	T	C	-	-	49314	0.261981	-	-	0.204567	0	128.0661	0
2769	T	C	-	-	4544	0.059744	-	-	0.049477	0	120.7518	0
2770	A	T	-	-	49314	0.228554	-	-	0.204567	0	111.7257	0
2771	G	C	-	-	49314	0.239552	-	-	0.204567	0	117.1019	0
2772	C	A	-	-	2728	0.050331	-	-	0.049477	0	101.7267	0
2775	C	T	-	-	6336	0.069514	-	-	0.049477	0	140.4985	0
2781	T	C	-	-	49314	0.214857	-	-	0.15509	0	138.5366	0
2784	T	C	-	-	49314	0.28531	500	0.294032	0.204567	0	139.4701	143.7339
Noise/ false calls												
111	A	G			2758	0.092265						
183	A	G			4549	0.101814						
312	G	A			2122	0.045456						
313	A	G			2540	0.050074						
425	A	G			258	0.045558						
932	G	A			81	0.045779						
1212	T	C			270	0.041161						
3417	T	C			715	0.063811						
3567	C	T			321	0.026232						
3597	A	G			321	0.036877						
4344	A	G			93	0.039297						
5213	T	C			2744	0.085755						
5531	C	T			6359	0.105348						
6342	C	G			292	0.041444						
6675	A	G			3615	0.062035						
6994	A	G			76	0.033792						

Barcode 09															
			Clair		LoFreq		Longshot		Expected AF	% Similarity to expected AF			Rating		
Pos	Ref	Alt	Q	Freq	Q	Freq	Q	Freq		Clair	Lof	Long	Clair	Lof	Long
2556	C	T	12.23	0.258	49314	0.240571	500	0.277125	0.205977	125.2565	116.7949	134.5414	☆	⯪	☆
2559	A	C	-	-	45979	0.205049	-	-	0.1961	0	104.5637	0	☆	★	☆
2562	C	T	-	-	47048	0.205119	-	-	0.1961	0	104.5994	0	☆	★	☆
2562	C	A	-	-	-	-	-	-	0.009878	0	0	0	☆	☆	☆
2565	T	C	-	-	49314	0.274243	-	-	0.1961	0	139.8489	0	☆	☆	☆
2566	A	T	-	-	-	-	-	-	0.009878	0	0	0	☆	☆	☆
2567	G	C	-	-	-	-	-	-	0.009878	0	0	0	☆	☆	☆
2568	T	C	-	-	49314	0.262013	-	-	0.1961	0	133.6122	0	☆	☆	☆
2571	C	T	-	-	49314	0.271053	-	-	0.205977	0	131.5936	0	☆	☆	☆
2574	C	T	-	-	-	-	-	-	0.009878	0	0	0	☆	☆	☆
2577	A	C	-	-	49314	0.264577	-	-	0.1961	0	134.9197	0	☆	☆	☆
2577	A	T	-	-	-	-	-	-	0.009878	0	0	0	☆	☆	☆
2580	G	C	-	-	49314	0.272005	500	0.279744	0.205977	0	132.0558	135.813	☆	☆	☆
2583	C	T	-	-	-	-	-	-	0.009878	0	0	0	☆	☆	☆
2586	G	C	8.2	0.2724	49314	0.265293	-	-	0.1961	138.909	135.2849	0	☆	☆	☆
2586	G	T	-	-	-	-	-	-	0.009878	0	0	0	☆	☆	☆
2589	T	C	-	-	49314	0.244004	500	0.27143	0.1961	0	124.4286	138.4144	☆	⯪	☆
2589	T	A	-	-	-	-	-	-	0.009878	0	0	0	☆	☆	☆
2592	G	A	11.41	0.2526	49314	0.254656	-	-	0.205977	122.6349	123.633	0	⯪	⯪	☆
2595	C	T	-	-	49314	0.23271	500	0.2647	0.1961	0	118.6693	134.9823	☆	⯪	☆
2595	C	A	-	-	-	-	-	-	0.009878	0	0	0	☆	☆	☆
2598	A	C	-	-	49314	0.254628	500	0.264745	0.1961	0	129.8463	135.0053	☆	☆	☆
2601	C	A	-	-	49314	0.268019	500	0.272422	0.205977	0	130.1206	132.2582	☆	☆	☆
2604	T	C	-	-	49314	0.271753	-	-	0.1961	0	138.5791	0	☆	☆	☆
2607	C	T	-	-	-	-	-	-	0.009878	0	0	0	☆	☆	☆
2608	A	T	-	-	-	-	-	-	0.990122	0	0	0	☆	☆	☆
2609	G	C	-	-	-	-	-	-	0.990122	0	0	0	☆	☆	☆
2610	C	T	-	-	-	-	-	-	0.009878	0	0	0	☆	☆	☆
2613	T	C	-	-	-	-	-	-	0.009878	0	0	0	☆	☆	☆
2619	T	G	-	-	49314	0.259403	500	0.269576	0.1961	0	132.2813	137.4692	☆	☆	☆
2622	C	T	-	-	226	0.032932	-	-	0.009878	0	333.3943	0	☆	☆	☆
2625	C	G	16.52	0.276	49314	0.258229	-	-	0.1961	140.7448	131.6826	0	☆	☆	☆
2625	C	T	-	-	-	-	-	-	0.009878	0	0	0	☆	☆	☆
2628	G	T	-	-	-	-	-	-	0.009878	0	0	0	☆	☆	☆
2631	T	C	14.31	0.251	49314	0.246277	500	0.269912	0.1961	127.9962	125.5878	137.6401	☆	☆	☆
2636	G	A	-	-	-	-	-	-	0.009878	0	0	0	☆	☆	☆
2637	C	T	-	-	-	-	-	-	0.009878	0	0	0	☆	☆	☆
2640	G	T	-	-	-	-	-	-	0.009878	0	0	0	☆	☆	☆
2643	T	C	5.03	0.2854	49314	0.27654	500	0.2936	0.205977	138.5589	134.2575	142.5399	☆	☆	☆
2646	C	T	-	-	49314	0.270191	-	-	0.1961	0	137.7826	0	☆	☆	☆
2649	C	A	-	-	-	-	-	-	0.009878	0	0	0	☆	☆	☆
2652	G	A	-	-	-	-	-	-	0.009878	0	0	0	☆	☆	☆
2655	A	G	-	-	49314	0.264299	-	-	0.1961	0	134.778	0	☆	☆	☆
2655	A	C	-	-	-	-	-	-	0.009878	0	0	0	☆	☆	☆
2661	C	A	-	-	49314	0.256018	500	0.265586	0.1961	0	130.5551	135.4343	☆	☆	☆
2661	C	T	-	-	-	-	-	-	0.009878	0	0	0	☆	☆	☆
2664	C	T	-	-	-	-	-	-	0.009878	0	0	0	☆	☆	☆
2667	C	T	-	-	-	-	-	-	0.009878	0	0	0	☆	☆	☆
2670	C	T	-	-	-	-	-	-	0.009878	0	0	0	☆	☆	☆
2673	T	C	5.8	0.2839	49314	0.27507	-	-	0.1961	144.7734	140.2706	0	☆	☆	☆
2673	T	A	-	-	-	-	-	-	0.009878	0	0	0	☆	☆	☆
2676	C	T	-	-	-	-	-	-	0.009878	0	0	0	☆	☆	☆
2679	A	G	19.77	0.3335	49314	0.289695	-	-	0.1961	170.0667	147.7285	0	☆	☆	☆
2682	T	G	-	-	49314	0.251165	500	0.268756	0.1961	0	128.0804	137.0507	☆	☆	☆
2685	A	C	-	-	49314	0.211643	-	-	0.1961	0	107.9263	0	☆	⯪	☆
2685	A	T	-	-	-	-	-	-	0.009878	0	0	0	☆	☆	☆
2688	C	T	-	-	-	-	-	-	0.009878	0	0	0	☆	☆	☆
2691	A	T	-	-	-	-	-	-	0.009878	0	0	0	☆	☆	☆
2695	C	A	-	-	49314	0.251705	500	0.26578	0.1961	0	128.3557	135.5334	☆	☆	☆
2697	A	G	-	-	49314	0.237114	500	0.253245	0.1961	0	120.9151	129.1412	☆	⯪	☆
2697	A	T	-	-	-	-	-	-	0.009878	0	0	0	☆	☆	☆
2700	A	G	-	-	49314	0.249379	500	0.269367	0.1961	0	127.1696	137.3623	☆	☆	☆
2700	A	T	-	-	-	-	-	-	0.009878	0	0	0	☆	☆	☆
2703	T	C	-	-	49314	0.225156	-	-	0.1961	0	114.8172	0	☆	⯪	☆
2704	T	A	-	-	49314	0.224163	500	0.241157	0.1961	0	114.3108	122.977	☆	⯪	⯪
2705	C	G	-	-	49314	0.235285	500	0.253823	0.1961	0	119.9824	129.4358	☆	⯪	☆

2706	C	T	-	-	-	-	-	-	0.009878	0	0	0	☆	☆	☆
2709	C	A	-	-	49314	0.245354	-	-	0.205977	0	119.117	0	☆	★	☆
2712	C	T	-	-	-	-	-	-	0.009878	0	0	0	☆	☆	☆
2713	T	A	-	-	49314	0.255816	500	0.27417	0.1961	0	130.4521	139.8118	☆	☆	☆
2714	C	G	-	-	49314	0.253973	500	0.264897	0.1961	0	129.5123	135.083	☆	☆	☆
2715	C	T	-	-	-	-	-	-	0.009878	0	0	0	☆	☆	☆
2718	C	T	-	-	-	-	-	-	0.009878	0	0	0	☆	☆	☆
2721	C	T	-	-	-	-	-	-	0.009878	0	0	0	☆	☆	☆
2721	C	A	-	-	49314	0.237274	500	0.26332	0.1961	0	120.9967	134.2788	☆	★	☆
2724	T	C	-	-	49314	0.246839	500	0.276985	0.1961	0	125.8743	141.2474	☆	☆	☆
2727	G	A	-	-	-	-	500	0.278622	0.009878	0	0	2820.695	☆	☆	☆
2730	A	C	-	-	49314	0.250157	500	0.279415	0.1961	0	127.5663	142.4861	☆	☆	☆
2731	C	A	-	-	49314	0.248964	500	0.271671	0.1961	0	126.958	138.5372	☆	☆	☆
2733	T	A	-	-	49314	0.259994	-	-	0.1961	0	132.5827	0	☆	☆	☆
2736	T	A	-	-	-	-	-	-	0.009878	0	0	0	☆	☆	☆
2736	T	C	-	-	49314	0.259426	-	-	0.1961	0	132.293	0	☆	☆	☆
2739	A	C	10.17	0.2737	49314	0.266882	500	0.276985	0.205977	132.8787	129.5686	134.4738	☆	☆	☆
2743	C	T	-	-	-	-	-	-	0.009878	0	0	0	☆	☆	☆
2745	G	A	-	-	-	-	-	-	0.009878	0	0	0	☆	☆	☆
2748	C	A	-	-	-	-	-	-	0.009878	0	0	0	☆	☆	☆
2751	T	G	-	-	-	-	-	-	0.009878	0	0	0	☆	☆	☆
2754	A	T	-	-	49314	0.270023	500	0.278622	0.205977	0	131.0936	135.2685	☆	☆	☆
2758	C	T	-	-	49314	0.269596	500	0.279415	0.1961	0	137.4792	142.4861	☆	☆	☆
2760	C	T	-	-	-	-	-	-	0.009878	0	0	0	☆	☆	☆
2763	T	G	-	-	49314	0.258341	500	0.271671	0.1961	0	131.7397	138.5372	☆	☆	☆
2763	T	C	-	-	-	-	-	-	0.009878	0	0	0	☆	☆	☆
2766	T	C	-	-	49314	0.261389	-	-	0.205977	0	126.9018	0	☆	☆	☆
2769	T	C	-	-	-	-	-	-	0.009878	0	0	0	☆	☆	☆
2770	A	T	-	-	49314	0.235561	-	-	0.205977	0	114.3626	0	☆	★	☆
2771	G	C	-	-	49314	0.249388	-	-	0.205977	0	121.0755	0	☆	★	☆
2772	C	A	-	-	-	-	-	-	0.009878	0	0	0	☆	☆	☆
2775	C	T	-	-	-	-	-	-	0.009878	0	0	0	☆	☆	☆
2781	T	C	-	-	49314	0.270362	-	-	0.1961	0	137.8698	0	☆	☆	☆
2784	T	C	6.39	0.2835	49314	0.289981	-	-	0.205977	137.6365	140.783	0	☆	☆	☆
Noise/ false calls															
111	A	G			2504	0.091977									
183	A	G			4462	0.104361									
312	G	A			1560	0.043258									
313	A	G			2084	0.048282									
425	A	G			179	0.044131									
1212	T	C			209	0.040179									
2722	T	A			87	0.024815									
3417	T	C			1044	0.067969									
3567	C	T			449	0.027728									
3597	A	G			333	0.037014									
5213	T	C			3151	0.089024									
5531	C	T			5432	0.103608									
6342	C	G			311	0.042239									
6675	A	G			2916	0.05924									

Barcode 10															
			Clair		LoFreq		Longshot		Expected AF	% Similarity to expected AF			Rating		
Pos	Ref	Alt	Q	Freq	Q	Freq	Q	Freq		Clair	Lof	Long	Clair	Lof	Long
2556	C	T	14.03	0.8373	49314	0.837024	500	0.888492	0.900016	93.03168	93.00102	98.71962	⯪	⯪	★
2559	A	C	-	-	5869	0.070627	-	-	0.103971	0	67.92974	0	☆	☆	☆
2562	C	T	-	-	49314	0.070766	-	-	0.103971	0	68.06343	0	☆	☆	☆
2562	C	A	10.6	0.786	49314	0.762578	-	-	0.796045	98.7381	95.79581	0	★	★	☆
2565	T	C	-	-	18665	0.093946	-	-	0.103971	0	90.35818	0	☆	⯪	☆
2566	A	T	8.64	0.7727	49314	0.747291	-	-	0.796045	97.06734	93.87544	0	★	⯪	☆
2567	G	C	13.65	0.7762	49314	0.748167	-	-	0.796045	97.50702	93.98548	0	★	⯪	☆
2568	T	C	-	-	11522	0.092188	-	-	0.103971	0	88.66732	0	☆	⯪	☆
2571	C	T	13.97	0.8239	49314	0.745489	500	0.867148	0.900016	91.54282	82.83064	96.34802	⯪	⯪	★
2574	C	T	8.87	0.8026	49314	0.757576	-	-	0.796045	100.8234	95.16745	0	★	★	☆
2577	A	C	-	-	49314	0.092783	-	-	0.103971	0	89.2396	0	☆	⯪	☆
2577	A	T	10.69	0.7983	49314	0.758252	-	-	0.796045	100.2832	95.25237	0	★	★	☆
2580	G	C	16.22	0.8737	49314	0.830136	500	0.867237	0.900016	97.07606	92.2357	96.358	★	⯪	★
2583	C	T	-	-	49314	0.733363	-	-	0.796045	0	92.12579	0	☆	⯪	☆
2586	G	C	-	-	49314	0.093178	-	-	0.103971	0	89.61952	0	☆	⯪	☆
2586	G	T	17.24	0.7765	49314	0.757566	500	0.780388	0.796045	97.5447	95.16619	98.03317	★	★	★
2589	T	C	-	-	49314	0.083519	-	-	0.103971	0	80.32939	0	☆	⯪	☆
2589	T	A	3.02	0.7702	49314	0.767369	-	-	0.796045	96.75329	96.39766	0	★	★	☆
2592	G	A	5.66	0.8232	49314	0.82098	-	-	0.900016	91.46505	91.21838	0	⯪	⯪	☆
2595	C	T	-	-	49314	0.080665	-	-	0.103971	0	77.58439	0	☆	⯪	☆
2595	C	A	15.87	0.7229	49314	0.7129	500	0.745845	0.796045	90.81142	89.55521	93.69376	⯪	⯪	⯪
2598	A	C	-	-	12080	0.087091	-	-	0.103971	0	83.76498	0	☆	⯪	☆
2601	C	A	10.11	0.8639	49314	0.859623	-	-	0.900016	95.98719	95.51197	0	★	★	☆
2604	T	C	-	-	16745	0.09696	-	-	0.103971	0	93.25708	0	☆	⯪	☆
2607	C	T	9.82	0.683	49314	0.658072	500	0.730606	0.796045	85.79914	82.66766	91.77948	⯪	⯪	⯪
2608	A	T	7.77	0.7566	49314	0.709437	-	-	0.203955	370.9647	347.8405	0	☆	☆	☆
2609	G	C	-	-	49314	0.656338	-	-	0.203955	0	321.8058	0	☆	☆	☆
2610	C	T	9.67	0.6958	49314	0.71483	-	-	0.796045	87.40709	89.79765	0	⯪	⯪	☆
2613	T	C	7.56	0.74	49314	0.752524	-	-	0.796045	92.95954	94.53281	0	⯪	⯪	☆
2619	T	G	-	-	9637	0.088708	-	-	0.103971	0	85.32023	0	☆	⯪	☆
2622	C	T	16.32	0.7967	49314	0.770985	-	-	0.796045	100.0822	96.8519	0	★	★	☆
2625	C	G	-	-	49314	0.089315	-	-	0.103971	0	85.90404	0	☆	⯪	☆
2625	C	T	18.23	0.7994	49314	0.783891	-	-	0.796045	100.4214	98.47317	0	★	★	☆
2628	G	T	9.72	0.7941	49314	0.796218	500	0.791723	0.796045	99.75563	100.0217	99.45697	★	★	★
2631	T	C	-	-	25769	0.083401	-	-	0.103971	0	80.2159	0	☆	⯪	☆
2636	G	A	12.91	0.7961	49314	0.794388	500	0.803481	0.796045	100.0069	99.79181	100.9341	★	★	★
2637	C	T	12.55	0.7998	49314	0.80446	500	0.80452	0.796045	100.4717	101.0571	101.0646	★	★	★
2640	G	T	18.76	0.7886	49314	0.794339	-	-	0.796045	99.06472	99.78565	0	★	★	☆
2643	T	C	18.55	0.8656	49314	0.860625	500	0.885958	0.900016	96.17607	95.62331	98.43806	★	★	★
2646	C	T	-	-	25504	0.09998	-	-	0.103971	0	96.16175	0	☆	★	☆
2649	C	A	14.03	0.6469	49314	0.705213	-	-	0.796045	81.26422	88.58956	0	⯪	⯪	☆
2652	G	A	5.96	0.766	49314	0.754917	500	0.799788	0.796045	96.22568	94.83342	100.4701	★	⯪	★
2655	A	G	8.71	0.7032	49314	0.088101	-	-	0.103971	676.3447	84.73641	0	☆	⯪	☆
2655	A	C	-	-	49314	0.731937	-	-	0.796045	0	91.94665	0	☆	⯪	☆
2661	C	A	-	-	49314	0.088864	-	-	0.103971	0	85.47027	0	☆	⯪	☆
2661	C	T	10.99	0.7899	49314	0.784073	500	0.808113	0.796045	99.22802	98.49603	101.516	★	★	★
2664	C	T	19.4	0.7958	49314	0.786698	500	0.797427	0.796045	99.96919	98.82578	100.1736	★	★	★
2667	C	T	16.76	0.7859	49314	0.784126	-	-	0.796045	98.72554	98.50269	0	★	★	☆
2670	C	T	17.69	0.7728	49314	0.768318	500	0.787268	0.796045	97.0799	96.51687	98.8974	★	★	★
2673	T	C	-	-	49314	0.09162	-	-	0.103971	0	88.12102	0	☆	⯪	☆
2673	T	A	11.96	0.735	49314	0.75003	500	0.752824	0.796045	92.33143	94.21951	94.57051	⯪	⯪	⯪
2676	C	T	13.27	0.791	49314	0.785703	500	0.790529	0.796045	99.36621	98.70079	99.30703	★	★	★
2679	A	G	-	-	13941	0.104604	-	-	0.103971	0	100.6092	0	☆	★	☆
2682	T	G	-	-	7345	0.086533	-	-	0.103971	0	83.22829	0	☆	⯪	☆
2685	A	C	-	-	49314	0.083229	-	-	0.103971	0	80.05047	0	☆	⯪	☆
2685	A	T	24.43	0.7285	49314	0.700289	-	-	0.796045	91.51489	87.971	0	⯪	⯪	☆
2688	C	T	17.32	0.7173	49314	0.733355	500	0.74738	0.796045	90.10794	92.12478	93.88656	⯪	⯪	⯪
2691	A	T	12.02	0.7545	49314	0.742743	500	0.762076	0.796045	94.78104	93.30411	95.73278	⯪	⯪	★
2695	C	A	-	-	16926	0.087034	-	-	0.103971	0	83.71016	0	☆	⯪	☆
2697	A	G	-	-	49314	0.080888	-	-	0.103971	0	77.79887	0	☆	⯪	☆
2697	A	T	11.44	0.7924	49314	0.783532	500	0.867263	0.796045	99.54208	98.42807	108.9464	★	★	⯪
2700	A	G	-	-	49314	0.085411	-	-	0.103971	0	82.14914	0	☆	⯪	☆
2700	A	T	10.04	0.7846	49314	0.796884	500	0.798001	0.796045	98.56223	100.1054	100.2456	★	★	★
2703	T	C	-	-	15757	0.077461	-	-	0.103971	0	74.50275	0	☆	☆	☆
2704	T	A	-	-	16011	0.076983	-	-	0.103971	0	74.04301	0	☆	☆	☆
2705	C	G	-	-	19191	0.080247	-	-	0.103971	0	77.18235	0	☆	⯪	☆

2706	C	T	7.17	0.7581	49314	0.762688	500	0.786005	0.796045	95.23328	95.80962	98.73871	★	★	★
2709	C	A	10.68	0.8322	49314	0.833621	-	-	0.900016	92.46503	92.62291	0	⯪	⯪	☆
2712	C	T	10.98	0.7871	49314	0.773584	-	-	0.796045	98.87628	97.17839	0	★	★	☆
2713	T	A	-	-	10090	0.087266	-	-	0.103971	0	83.9333	0	☆	⯪	☆
2714	C	G	-	-	14379	0.086928	-	-	0.103971	0	83.6082	0	☆	⯪	☆
2715	C	T	6.13	0.7578	49314	0.758336	500	0.776792	0.796045	95.19559	95.26292	97.58136	★	★	★
2718	C	T	7.65	0.7861	49314	0.786941	-	-	0.796045	98.75066	98.85631	0	★	★	☆
2721	C	T	10.16	0.6321	49314	0.712681	500	0.793567	0.796045	79.40503	89.5277	99.68868	⯪	⯪	★
2721	C	A	-	-	49314	0.080264	-	-	0.103971	0	77.1987	0	☆	⯪	☆
2724	T	C	-	-	20251	0.085828	-	-	0.103971	0	82.55021	0	☆	⯪	☆
2727	G	A	7.26	0.7592	49314	0.788014	500	0.791386	0.796045	95.37146	98.9911	99.41475	★	★	★
2730	A	C	-	-	10271	0.084132	-	-	0.103971	0	80.91898	0	☆	⯪	☆
2731	C	A	-	-	9622	0.084199	-	-	0.103971	0	80.98342	0	☆	⯪	☆
2733	T	A	-	-	15506	0.088303	-	-	0.103971	0	84.93069	0	☆	⯪	☆
2736	T	A	9.67	0.7476	49314	0.761732	500	0.768794	0.796045	93.91425	95.68953	96.57669	⯪	★	★
2736	T	C	-	-	49314	0.086954	-	-	0.103971	0	83.63321	0	☆	⯪	☆
2739	A	C	9.86	0.8772	49314	0.837172	500	0.846165	0.900016	97.46494	93.01746	94.01671	★	⯪	⯪
2743	C	T	10.17	0.7622	49314	0.765123	500	0.772666	0.796045	95.74832	96.11551	97.06307	★	★	★
2745	G	A	5.59	0.7704	49314	0.765245	500	0.76022	0.796045	96.77841	96.13084	95.49962	★	★	★
2748	C	A	4.71	0.7422	49314	0.726997	500	0.757624	0.796045	93.2359	91.32609	95.17343	⯪	⯪	★
2751	T	G	3.88	0.693	49314	0.643214	-	-	0.796045	87.05535	80.80118	0	⯪	⯪	☆
2754	A	T	-	-	49314	0.856416	500	0.862357	0.900016	0	95.15565	95.81578	☆	★	★
2758	C	T	-	-	27569	0.101377	-	-	0.103971	0	97.50539	0	☆	★	☆
2760	C	T	7.63	0.7675	49314	0.772694	-	-	0.796045	96.41411	97.06659	0	★	★	☆
2763	T	G	-	-	49314	0.088856	-	-	0.103971	0	85.46257	0	☆	⯪	☆
2763	T	C	7.22	0.7518	49314	0.770574	500	0.790611	0.796045	94.44186	96.80027	99.3173	⯪	★	★
2766	T	C	6.59	0.8528	49314	0.844183	-	-	0.900016	94.75388	93.79645	0	⯪	⯪	☆
2769	T	C	4.37	0.6371	49314	0.645278	-	-	0.796045	80.03314	81.06046	0	⯪	⯪	☆
2770	A	T	6.7	0.7082	49314	0.700826	-	-	0.900016	78.68749	77.86818	0	⯪	⯪	☆
2771	G	C	10.38	0.7094	49314	0.715212	-	-	0.900016	78.82083	79.46659	0	⯪	⯪	☆
2772	C	A	8.25	0.6228	49314	0.636613	-	-	0.796045	78.23675	79.97196	0	⯪	⯪	☆
2775	C	T	-	-	49314	0.723875	500	0.772642	0.796045	0	90.9339	97.06005	☆	⯪	★
2781	T	C	-	-	17693	0.096304	-	-	0.103971	0	92.62613	0	☆	⯪	☆
2784	T	C	20.91	0.8873	49314	0.882928	-	-	0.900016	98.58714	98.10137	0	★	★	☆
Noise/ false calls															
111	A	G			66463	0.255676									
166	A	G			66609	0.091594									
183	A	G			66649	0.277679									
312	G	A			66716	0.043438									
313	A	G			66727	0.048766									
425	A	G			66761	0.045251									
932	G	A			66833	0.045083									
1212	T	C			67244	0.03752									
2532	C	T			67187	0.045321									
2558	C	T			66636	0.036602									
2566	A	C			49314	0.030729									
2609	G	T			49314	0.097166									
2686	C	T			429	0.041779									
2770	A	C			49314	0.029458									
3417	T	C			1325	0.067246									
3567	C	T			445	0.026322									
3597	A	G			537	0.038033									
3665	A	G			66	0.057311									
4066	A	G			1405	0.111764									
4763	T	A			64	0.032555									
5213	T	C			49314	0.262929									
5531	C	T			7951	0.105676									
6295	A	G			65	0.051383									
6342	C	G			482	0.042803									
6413	A	G			86	0.048307									
6416	A	G			160	0.062677									
6675	A	G			4044	0.059942									
6994	A	G			149	0.035085									

Barcode 11															
			Clair		LoFreq		Longshot		Expected AF	% Similarity to expected AF			Rating		
Pos	Ref	Alt	Q	Freq	Q	Freq	Q	Freq		Clair	Lof	Long	Clair	Lof	Long
2556	C	T	11.88	0.8027	49314	0.807943	500	0.401564	0.849724	94.46593	95.08295	47.25819	⯪	★	☆
2559	A	C	-	-	527	0.041391	-	-	0.052089	0	79.46183	0	☆	⯪	☆
2562	C	T	-	-	49314	0.041073	-	-	0.052089	0	78.85134	0	☆	⯪	☆
2562	C	A	5.98	0.7937	49314	0.766643	-	-	0.797635	99.50664	96.11449	0	★	★	☆
2565	T	C	-	-	6875	0.057933	-	-	0.052089	0	111.2189	0	☆	⯪	☆
2566	A	T	-	-	49314	0.751281	-	-	0.797635	0	94.18854	0	☆	⯪	☆
2567	G	C	8.89	0.7801	49314	0.75249	-	-	0.797635	97.8016	94.34012	0	★	⯪	☆
2568	T	C	-	-	2790	0.056258	-	-	0.052089	0	108.0033	0	☆	⯪	☆
2571	C	T	-	-	49314	0.710983	-	-	0.849724	0	83.67219	0	☆	⯪	☆
2574	C	T	7.24	0.8165	49314	0.76021	-	-	0.797635	102.3651	95.30798	0	★	★	☆
2577	A	C	-	-	49314	0.053959	-	-	0.052089	0	103.5897	0	☆	★	☆
2577	A	T	10.19	0.8073	49314	0.761013	-	-	0.797635	101.2117	95.40865	0	★	★	☆
2580	G	C	11.55	0.8405	49314	0.793313	-	-	0.849724	98.91443	93.36121	0	★	⯪	☆
2583	C	T	-	-	49314	0.735322	-	-	0.797635	0	92.18776	0	☆	⯪	☆
2586	G	C	-	-	49314	0.055859	-	-	0.052089	0	107.2373	0	☆	⯪	☆
2586	G	T	13.86	0.7642	49314	0.759082	500	0.101644	0.797635	95.80821	95.16656	12.74315	★	★	☆
2589	T	C	-	-	49314	0.049441	-	-	0.052089	0	94.9161	0	☆	⯪	☆
2589	T	A	6.14	0.7694	49314	0.769452	-	-	0.797635	96.46013	96.46665	0	★	★	☆
2592	G	A	-	-	49314	0.788107	-	-	0.849724	0	92.74855	0	☆	⯪	☆
2595	C	T	-	-	49314	0.051157	-	-	0.052089	0	98.21046	0	☆	★	☆
2595	C	A	17.78	0.7135	49314	0.712319	500	0.748925	0.797635	89.45192	89.30386	93.89311	⯪	⯪	⯪
2598	A	C	-	-	2759	0.051196	-	-	0.052089	0	98.28533	0	☆	★	☆
2601	C	A	7.14	0.8332	49314	0.825522	500	0.319419	0.849724	98.05533	97.15174	37.59093	★	★	☆
2604	T	C	-	-	5479	0.059342	-	-	0.052089	0	113.9239	0	☆	⯪	☆
2607	C	T	17.2	0.7014	49314	0.664533	-	-	0.797635	87.93493	83.3129	0	⯪	⯪	☆
2608	A	T	8.18	0.768	49314	0.715883	-	-	0.202365	379.5127	353.7587	0	☆	☆	☆
2609	G	C	-	-	49314	0.66123	-	-	0.202365	0	326.7515	0	☆	☆	☆
2610	C	T	9.87	0.7075	49314	0.71989	-	-	0.797635	88.69969	90.25304	0	⯪	⯪	☆
2613	T	C	15.38	0.7449	49314	0.758808	500	0.734868	0.797635	93.38855	95.13221	92.13088	⯪	★	⯪
2619	T	G	-	-	1228	0.050792	-	-	0.052089	0	97.50973	0	☆	★	☆
2622	C	T	20.17	0.7998	49314	0.776864	-	-	0.797635	100.2714	97.3959	0	★	★	☆
2625	C	G	-	-	49314	0.052094	-	-	0.052089	0	100.0093	0	☆	★	☆
2625	C	T	15.62	0.7985	49314	0.787974	-	-	0.797635	100.1084	98.78877	0	★	★	☆
2628	G	T	9.77	0.8019	49314	0.800181	500	0.199277	0.797635	100.5347	100.3192	24.98353	★	★	☆
2631	T	C	-	-	10826	0.048328	-	-	0.052089	0	92.77938	0	☆	⯪	☆
2636	G	A	11.18	0.7885	49314	0.798447	500	0.232496	0.797635	98.85471	100.1018	29.14812	★	★	☆
2637	C	T	16.89	0.7976	49314	0.809471	500	0.237822	0.797635	99.99558	101.4839	29.8159	★	★	☆
2640	G	T	12.29	0.7853	49314	0.798433	500	0.245553	0.797635	98.45353	100.1	30.78507	★	★	☆
2643	T	C	14.42	0.8262	49314	0.827883	-	-	0.849724	97.23153	97.42959	0	★	★	☆
2646	C	T	-	-	10890	0.062657	-	-	0.052089	0	120.288	0	☆	⯪	☆
2649	C	A	10.67	0.6596	49314	0.710179	-	-	0.797635	82.69444	89.03556	0	⯪	⯪	☆
2652	G	A	11.55	0.7618	49314	0.758811	500	0.223784	0.797635	95.50732	95.13259	28.05587	★	★	☆
2655	A	G	-	-	49314	0.051195	-	-	0.052089	0	98.28341	0	☆	★	☆
2655	A	C	10.28	0.7087	49314	0.735196	-	-	0.797635	88.85014	92.17196	0	⯪	⯪	☆
2661	C	A	-	-	49314	0.051983	-	-	0.052089	0	99.7962	0	☆	★	☆
2661	C	T	15.07	0.7955	49314	0.790641	500	0.261004	0.797635	99.73231	99.12313	32.72223	★	★	☆
2664	C	T	18.6	0.7917	49314	0.79305	-	-	0.797635	99.2559	99.42515	0	★	★	☆
2667	C	T	7.35	0.7776	49314	0.78997	-	-	0.797635	97.48817	99.03901	0	★	★	☆
2670	C	T	9.14	0.7642	49314	0.773828	-	-	0.797635	95.80821	97.01528	0	★	★	☆
2673	T	C	-	-	49314	0.053793	-	-	0.052089	0	103.271	0	☆	★	☆
2673	T	A	9.49	0.737	49314	0.754619	500	0.74655	0.797635	92.39813	94.60703	93.59543	⯪	⯪	⯪
2676	C	T	16.68	0.7901	49314	0.791395	-	-	0.797635	99.05531	99.21766	0	★	★	☆
2679	A	G	-	-	4893	0.068415	-	-	0.052089	0	131.3421	0	☆	☆	☆
2682	T	G	-	-	574	0.049796	-	-	0.052089	0	95.59763	0	☆	★	☆
2685	A	C	-	-	49314	0.052173	-	-	0.052089	0	100.161	0	☆	★	☆
2685	A	T	23.68	0.7347	49314	0.703306	-	-	0.797635	92.10977	88.17389	0	⯪	⯪	☆
2688	C	T	10.9	0.7166	49314	0.738129	-	-	0.797635	89.84057	92.53967	0	⯪	⯪	☆
2691	A	T	16.57	0.7628	49314	0.746894	-	-	0.797635	95.63269	93.63854	0	★	⯪	☆
2695	C	A	-	-	5043	0.05088	-	-	0.052089	0	97.67867	0	☆	★	☆
2697	A	G	-	-	49314	0.046605	-	-	0.052089	0	89.47159	0	☆	⯪	☆
2697	A	T	14.37	0.7892	49314	0.788067	-	-	0.797635	98.94247	98.80043	0	★	★	☆
2700	A	G	-	-	49314	0.049432	-	-	0.052089	0	94.89883	0	☆	⯪	☆
2700	A	T	17.09	0.7903	49314	0.802639	500	0.235677	0.797635	99.08038	100.6273	29.54696	★	★	☆
2703	T	C	-	-	4671	0.044605	-	-	0.052089	0	85.63202	0	☆	⯪	☆
2704	T	A	-	-	4632	0.043872	-	-	0.052089	0	84.22482	0	☆	⯪	☆
2705	C	G	-	-	6464	0.045891	-	-	0.052089	0	88.10087	0	☆	⯪	☆

2706	C	T	4.01	0.743	49314	0.765187	-	-	0.797635	93.15035	95.93195	0	⯪	★	☆
2709	C	A	9.29	0.7835	49314	0.802963	-	-	0.849724	92.20637	94.49688	0	⯪	⯪	☆
2712	C	T	7.38	0.8004	49314	0.779866	-	-	0.797635	100.3466	97.77226	0	★	★	☆
2713	T	A	-	-	1493	0.049909	-	-	0.052089	0	95.81456	0	☆	★	☆
2714	C	G	-	-	3506	0.050225	-	-	0.052089	0	96.42122	0	☆	★	☆
2715	C	T	10.75	0.7641	49314	0.761655	-	-	0.797635	95.79567	95.48914	0	★	★	☆
2718	C	T	7.47	0.7957	49314	0.790536	-	-	0.797635	99.75738	99.10997	0	★	★	☆
2721	C	T	7.82	0.648	49314	0.716918	500	0.115657	0.797635	81.24014	89.88043	14.5	⯪	⯪	☆
2721	C	A	-	-	49314	0.046904	-	-	0.052089	0	90.04561	0	☆	⯪	☆
2724	T	C	-	-	8131	0.052001	-	-	0.052089	0	99.83075	0	☆	★	☆
2727	G	A	4.87	0.7658	49314	0.794476	500	0.101493	0.797635	96.0088	99.60393	12.7243	★	★	☆
2730	A	C	-	-	1491	0.048503	-	-	0.052089	0	93.11535	0	☆	⯪	☆
2731	C	A	-	-	1293	0.048431	-	-	0.052089	0	92.97712	0	☆	⯪	☆
2733	T	A	-	-	3944	0.050645	-	-	0.052089	0	97.22753	0	☆	★	☆
2736	T	A	5.63	0.7535	49314	0.767684	500	0.054814	0.797635	94.46674	96.245	6.872077	⯪	★	☆
2736	T	C	-	-	49314	0.050211	-	-	0.052089	0	96.39434	0	☆	★	☆
2739	A	C	12.07	0.8352	49314	0.804928	-	-	0.849724	98.2907	94.72813	0	★	⯪	☆
2743	C	T	12.08	0.775	49314	0.771439	-	-	0.797635	97.16221	96.71576	0	★	★	☆
2745	G	A	12.06	0.7759	49314	0.771864	-	-	0.797635	97.27504	96.76905	0	★	★	☆
2748	C	A	10.7	0.7366	49314	0.733571	500	0.103448	0.797635	92.34798	91.96823	12.96937	⯪	⯪	☆
2751	T	G	10.65	0.706	49314	0.649008	-	-	0.797635	88.51164	81.36652	0	⯪	⯪	☆
2754	A	T	-	-	49314	0.826136	500	0.369692	0.849724	0	97.224	43.50728	☆	★	☆
2758	C	T	-	-	13097	0.064228	-	-	0.052089	0	123.304	0	☆	⯪	☆
2760	C	T	-	-	49314	0.778303	-	-	0.797635	0	97.57631	0	☆	★	☆
2763	T	G	-	-	49314	0.051887	-	-	0.052089	0	99.6119	0	☆	★	☆
2763	T	C	5.42	0.7472	49314	0.774089	500	0.260324	0.797635	93.67691	97.048	32.63693	⯪	★	☆
2766	T	C	-	-	49314	0.813444	500	0.352469	0.849724	0	95.73034	41.48042	☆	★	☆
2769	T	C	-	-	49314	0.647177	500	0.092792	0.797635	0	81.13696	11.63333	☆	⯪	☆
2770	A	T	-	-	49314	0.669749	-	-	0.849724	0	78.81956	0	☆	⯪	☆
2771	G	C	-	-	49314	0.682175	-	-	0.849724	0	80.28191	0	☆	⯪	☆
2772	C	A	5.78	0.6124	49314	0.637751	-	-	0.797635	76.77695	79.95522	0	⯪	⯪	☆
2775	C	T	10.5	0.691	49314	0.727226	500	0.096784	0.797635	86.63108	91.17275	12.13386	⯪	⯪	☆
2781	T	C	-	-	5551	0.057796	-	-	0.052089	0	110.9559	0	☆	⯪	☆
2784	T	C	12.08	0.8416	49314	0.850885	-	-	0.849724	99.04388	100.1366	0	★	★	☆

Noise/ false calls								
111	A	G			49314	0.247605		
166	A	G			1724	0.08694		
183	A	G			49314	0.270905		
312	G	A			2840	0.043113		
313	A	G			3915	0.04874		
425	A	G			421	0.045629		
932	G	A			195	0.047354		
1212	T	C			269	0.03731		
2532	C	T			405	0.044294		
2558	C	T			216	0.03228		
2566	A	C			49314	0.031844		
2567	G	T			49314	0.034905		
2609	G	T			49314	0.097867		
2686	C	T			669	0.042755		
2770	A	C			49314	0.028524		
3417	T	C			1997	0.067891		
3567	C	T			570	0.026484		
3597	A	G			741	0.038417		
3665	A	G			136	0.057468		
4066	A	G			1503	0.105793		
5213	T	C			49314	0.252812		
5531	C	T			12087	0.106121		
6295	A	G			320	0.054959		
6413	A	G			153	0.048046		
6416	A	G			199	0.061609		
6675	A	G			6685	0.062605		
6994	A	G			225	0.035429		

Barcode 12

Pos	Ref	Alt	Clair Q	Clair Freq	LoFreq Q	LoFreq Freq	Longshot Q	Longshot Freq	Expected AF	% Similarity to expected AF: Clair	Lof	Long	Rating: Clair	Lof	Long
2556	C	T	12.99	0.7724	49314	0.786557	500	0.825661	0.809346	95.43505	97.18424	102.0158	★	★	★
2559	A	C	-	-	-	-	-	-	0.010435	0	0	0	☆	☆	☆
2562	C	T	-	-	-	-	-	-	0.010435	0	0	0	☆	☆	☆
2562	C	A	12.54	0.7927	49314	0.783774	500	0.809499	0.798912	99.22247	98.10521	101.3252	★	★	★
2565	T	C	-	-	-	-	-	-	0.010435	0	0	0	☆	☆	☆
2566	A	T	-	-	49314	0.767401	500	0.804106	0.798912	0	96.05579	100.6501	☆	★	★
2567	G	C	-	-	49314	0.768419	-	-	0.798912	0	96.18322	0	☆	★	☆
2568	T	C	-	-	-	-	-	-	0.010435	0	0	0	☆	☆	☆
2571	C	T	13.37	0.7675	49314	0.684694	500	0.808172	0.809346	94.82962	84.5984	99.85488	⯨	⯨	★
2574	C	T	10.99	0.8097	49314	0.777406	-	-	0.798912	101.3504	97.30812	0	★	★	☆
2577	A	C	-	-	-	-	-	-	0.010435	0	0	0	☆	☆	☆
2577	A	T	5.03	0.7995	49314	0.776711	-	-	0.798912	100.0736	97.22113	0	★	★	☆
2580	G	C	17.14	0.801	49314	0.766297	500	0.800425	0.809346	98.96877	94.68099	98.89774	★	⯨	★
2583	C	T	11.27	0.7805	49314	0.753563	-	-	0.798912	97.6954	94.32369	0	★	⯨	☆
2586	G	C	-	-	-	-	-	-	0.010435	0	0	0	☆	☆	☆
2586	G	T	12.62	0.7773	49314	0.774875	500	0.797058	0.798912	97.29485	96.99131	99.76803	★	★	★
2589	T	C	-	-	-	-	-	-	0.010435	0	0	0	☆	☆	☆
2589	T	A	15.63	0.7706	49314	0.786764	-	-	0.798912	96.45621	98.47946	0	★	★	☆
2592	G	A	11.49	0.7319	49314	0.765705	-	-	0.809346	90.43101	94.60784	0	⯨	⯨	☆
2595	C	T	-	-	-	-	-	-	0.010435	0	0	0	☆	☆	☆
2595	C	A	16.71	0.7241	49314	0.732719	500	0.764176	0.798912	90.63579	91.71464	95.65208	⯨	⯨	★
2598	A	C	-	-	-	-	-	-	0.010435	0	0	0	☆	☆	☆
2601	C	A	9	0.7925	49314	0.799748	500	0.807509	0.809346	97.91854	98.81407	99.77294	★	★	★
2604	T	C	-	-	-	-	-	-	0.010435	0	0	0	☆	☆	☆
2607	C	T	11.75	0.6896	49314	0.682872	500	0.749187	0.798912	86.31742	85.47527	93.77594	⯨	⯨	⯨
2608	A	T	7.96	0.7605	49314	0.733579	-	-	0.201088	378.1921	364.8045	0	☆	☆	☆
2609	G	C	-	-	49314	0.680805	-	-	0.201088	0	338.5603	0	☆	☆	☆
2610	C	T	15.76	0.706	49314	0.740427	-	-	0.798912	88.37021	92.67945	0	⯨	⯨	☆
2613	T	C	9.8	0.7528	49314	0.778743	500	0.752814	0.798912	94.22818	97.47547	94.22993	⯨	★	⯨
2619	T	G	-	-	-	-	-	-	0.010435	0	0	0	☆	☆	☆
2622	C	T	-	-	49314	0.792532	-	-	0.798912	0	99.20145	0	☆	★	☆
2625	C	G	-	-	-	-	-	-	0.010435	0	0	0	☆	☆	☆
2625	C	T	12.68	0.8042	49314	0.805236	-	-	0.798912	100.6619	100.7916	0	★	★	☆
2628	G	T	6.38	0.8073	49314	0.818115	500	0.812231	0.798912	101.05	102.4037	101.6672	★	★	★
2631	T	C	-	-	-	-	-	-	0.010435	0	0	0	☆	☆	☆
2636	G	A	13.23	0.7903	49314	0.814648	500	0.822336	0.798912	98.92207	101.9697	102.9321	★	★	★
2637	C	T	14.99	0.7985	49314	0.827228	500	0.824137	0.798912	99.94846	103.5444	103.1575	★	★	★
2640	G	T	12.89	0.794	49314	0.816409	500	0.825682	0.798912	99.3852	102.1901	103.3509	★	★	★
2643	T	C	15.25	0.7923	49314	0.800294	-	-	0.809346	97.89383	98.88154	0	★	★	☆
2646	C	T	-	-	-	-	-	-	0.010435	0	0	0	☆	☆	☆
2649	C	A	11.6	0.6663	49314	0.726886	-	-	0.798912	83.40095	90.98452	0	⯨	⯨	☆
2652	G	A	14.05	0.7578	49314	0.774487	500	0.818739	0.798912	94.85403	96.94275	102.4818	⯨	★	★
2655	A	G	-	-	-	-	-	-	0.010435	0	0	0	☆	☆	☆
2655	A	C	16.85	0.7128	49314	0.752658	500	0.777096	0.798912	89.22137	94.21041	97.26938	⯨	⯨	★
2661	C	A	-	-	-	-	-	-	0.010435	0	0	0	☆	☆	☆
2661	C	T	12.52	0.7966	49314	0.808678	500	0.829605	0.798912	99.71064	101.2224	103.8419	★	★	★
2664	C	T	9.11	0.8007	49314	0.811147	-	-	0.798912	100.2238	101.5315	0	★	★	☆
2667	C	T	4.1	0.7888	49314	0.807458	-	-	0.798912	98.73431	101.0697	0	★	★	☆
2670	C	T	7.23	0.7703	49314	0.790307	-	-	0.798912	96.41866	98.92294	0	★	★	☆
2673	T	C	-	-	-	-	-	-	0.010435	0	0	0	☆	☆	☆
2673	T	A	11.59	0.7382	49314	0.771233	-	-	0.798912	92.4007	96.53544	0	⯨	★	☆
2676	C	T	20.21	0.7947	49314	0.808023	500	0.797436	0.798912	99.47282	101.1405	99.81527	★	★	★
2679	A	G	-	-	-	-	-	-	0.010435	0	0	0	☆	☆	☆
2682	T	G	-	-	-	-	-	-	0.010435	0	0	0	☆	☆	☆
2685	A	C	-	-	-	-	-	-	0.010435	0	0	0	☆	☆	☆
2685	A	T	5.26	0.7373	49314	0.717949	-	-	0.798912	92.28804	89.86587	0	⯨	⯨	☆
2688	C	T	6.14	0.7187	49314	0.753004	-	-	0.798912	89.95987	94.25372	0	⯨	⯨	☆
2691	A	T	5.41	0.7579	49314	0.764079	500	0.785877	0.798912	94.86655	95.63998	98.36843	⯨	★	★
2695	C	A	-	-	-	-	-	-	0.010435	0	0	0	☆	☆	☆
2697	A	G	-	-	-	-	-	-	0.010435	0	0	0	☆	☆	☆
2697	A	T	13.84	0.7931	49314	0.803008	-	-	0.798912	99.27254	100.5127	0	★	★	☆
2700	A	G	-	-	-	-	-	-	0.010435	0	0	0	☆	☆	☆
2700	A	T	13.27	0.7919	49314	0.819219	500	0.817371	0.798912	99.12234	102.5419	102.3105	★	★	★
2703	T	C	-	-	-	-	-	-	0.010435	0	0	0	☆	☆	☆
2704	T	A	-	-	-	-	-	-	0.010435	0	0	0	☆	☆	☆
2705	C	G	-	-	-	-	-	-	0.010435	0	0	0	☆	☆	☆

2706	C	T	9.23	0.7556	49314	0.780538	500	0.803234	0.798912	94.57866	97.70015	100.541	⯪	★	★
2709	C	A	11.57	0.7475	49314	0.781633	-	-	0.809346	92.35849	96.57585	0	⯪	★	☆
2712	C	T	15.63	0.7951	49314	0.795944	-	-	0.798912	99.52288	99.62853	0	★	★	☆
2713	T	A	-	-	-	-	-	-	0.010435	0	0	0	☆	☆	☆
2714	C	G	-	-	-	-	-	-	0.010435	0	0	0	☆	☆	☆
2715	C	T	11.06	0.7664	49314	0.778032	-	-	0.798912	95.9305	97.38648	0	★	★	☆
2718	C	T	11.82	0.797	49314	0.807537	-	-	0.798912	99.76071	101.0796	0	★	★	☆
2721	C	T	16.11	0.6325	49314	0.731437	500	0.784507	0.798912	79.1702	91.55417	98.19692	⯪	⯪	★
2721	C	A	11.92	0.7633	-	-	-	-	0.010435	7315.153	0	0	☆	☆	☆
2724	T	C	-	-	-	-	-	-	0.010435	0	0	0	☆	☆	☆
2727	G	A	-	-	49314	0.810956	500	0.781412	0.798912	0	101.5076	97.80958	☆	★	★
2730	A	C	-	-	-	-	-	-	0.010435	0	0	0	☆	☆	☆
2731	C	A	-	-	-	-	-	-	0.010435	0	0	0	☆	☆	☆
2733	T	A	-	-	-	-	-	-	0.010435	0	0	0	☆	☆	☆
2736	T	A	12.4	0.7533	49314	0.78247	-	-	0.798912	94.29077	97.94198	0	⯪	★	☆
2736	T	C	-	-	-	-	-	-	0.010435	0	0	0	☆	☆	☆
2739	A	C	12.72	0.7829	49314	0.776978	-	-	0.809346	96.73239	96.00069	0	★	★	☆
2743	C	T	4.43	0.767	49314	0.788136	500	0.805136	0.798912	96.0056	98.6512	100.7791	★	★	★
2745	G	A	17.07	0.7717	49314	0.787558	-	-	0.798912	96.5939	98.57885	0	★	★	☆
2748	C	A	14.5	0.7426	49314	0.747632	500	0.777083	0.798912	92.95144	93.5813	97.2677	⯪	⯪	★
2751	T	G	13.17	0.6974	49314	0.660204	-	-	0.798912	87.29375	82.63791	0	⯪	⯪	☆
2754	A	T	-	-	49314	0.799954	500	0.805498	0.809346	0	98.83953	99.5245	☆	★	★
2758	C	T	-	-	-	-	-	-	0.010435	0	0	0	☆	☆	☆
2760	C	T	8.99	0.7724	49314	0.79554	-	-	0.798912	96.68152	99.57796	0	★	★	☆
2763	T	G	-	-	-	-	-	-	0.010435	0	0	0	☆	☆	☆
2763	T	C	6.6	0.7512	49314	0.792573	500	0.822519	0.798912	94.02791	99.20658	102.9549	⯪	★	★
2766	T	C	-	-	49314	0.792548	500	0.80501	0.809346	0	97.92447	99.46429	☆	★	★
2769	T	C	-	-	49314	0.66571	500	0.779081	0.798912	0	83.3271	97.51781	☆	⯪	★
2770	A	T	-	-	49314	0.650987	-	-	0.809346	0	80.43368	0	☆	⯪	☆
2771	G	C	-	-	49314	0.661135	-	-	0.809346	0	81.68754	0	☆	⯪	☆
2772	C	A	4.83	0.6375	49314	0.657856	-	-	0.798912	79.79605	82.34401	0	⯪	⯪	☆
2775	C	T	-	-	49314	0.746045	500	0.779146	0.798912	0	93.38266	97.52596	☆	⯪	★
2781	T	C	-	-	-	-	-	-	0.010435	0	0	0	☆	☆	☆
2784	T	C	19.66	0.7934	49314	0.822883	-	-	0.809346	98.02974	101.6726	0	★	★	☆

Noise/ false calls								
111	A	G			33243	0.237861	500	0.295338
166	A	G			1237	0.087181		
183	A	G			42297	0.259663		
312	G	A			1827	0.042304		
313	A	G			2453	0.047595		
425	A	G			390	0.046575		
1212	T	C			158	0.037108		
2532	C	T			288	0.043742		
2566	A	C			49314	0.031545		
2567	G	T			49314	0.03474		
2609	G	T			49314	0.09784		
2686	C	T			659	0.044438		
3417	T	C			1080	0.065505		
3567	C	T			381	0.025529		
3597	A	G			529	0.037854		
3665	A	G			117	0.057248		
4066	A	G			745	0.102304		
4131	C	T			161	0.039294		
4344	A	G			182	0.040302		
5213	T	C			45928	0.246301		
5531	C	T			7681	0.105117		
6295	A	G			140	0.052965		
6342	C	G			540	0.042947		
6416	A	G			112	0.059928		
6994	A	G			101	0.033762		
