# Supplementary material for: Sub-consensus haploid variant calling in long-read sequencing technology
Source: PLoS One. 2026 Oct 5;21(10):e0356259. doi: 10.1371/journal.pone.0356259 (PMC13638048; doi:10.1371/journal.pone.0356259)

Supplementary Sheet 2: Sequence-Context Error Analysis

Methods

Homopolymer: Homopolymer-associated indels were identified and quantified using Qualimap (v2.2.2-dev) on clonal *E. coli* K-12 genomes sequenced with R10.4.1 flow cells. Indels were classified as occurring within homopolymeric regions (polyA, polyC, polyG, polyT; ≥3 consecutive identical nucleotides) or non-homopolymeric regions.

http://qualimap.conesalab.org/

```
qualimap bamqc -nr 250 -bam [barcode].bam
```

K-mer Analysis: K-mer signatures (3-mer through 7-mer) were computed using sourmash (v4.6.1) with the --singleton flag. Distance matrices were generated using sourmash compare -p 8, and correlation heatmaps were visualized using sourmash plot.

https://sourmash.readthedocs.io/en/latest/command-line.html

```
#!/bin/bash

echo input sig file, only single file operation

read infile

echo what is the target K?

read kval

sourmash compute -k $kval --singleton $infile
sourmash compare -p 8 *sig --output "$kval"_graph.matrix
sourmash plot *.matrix

echo complete
```

Results

<u>Homopolymer</u>

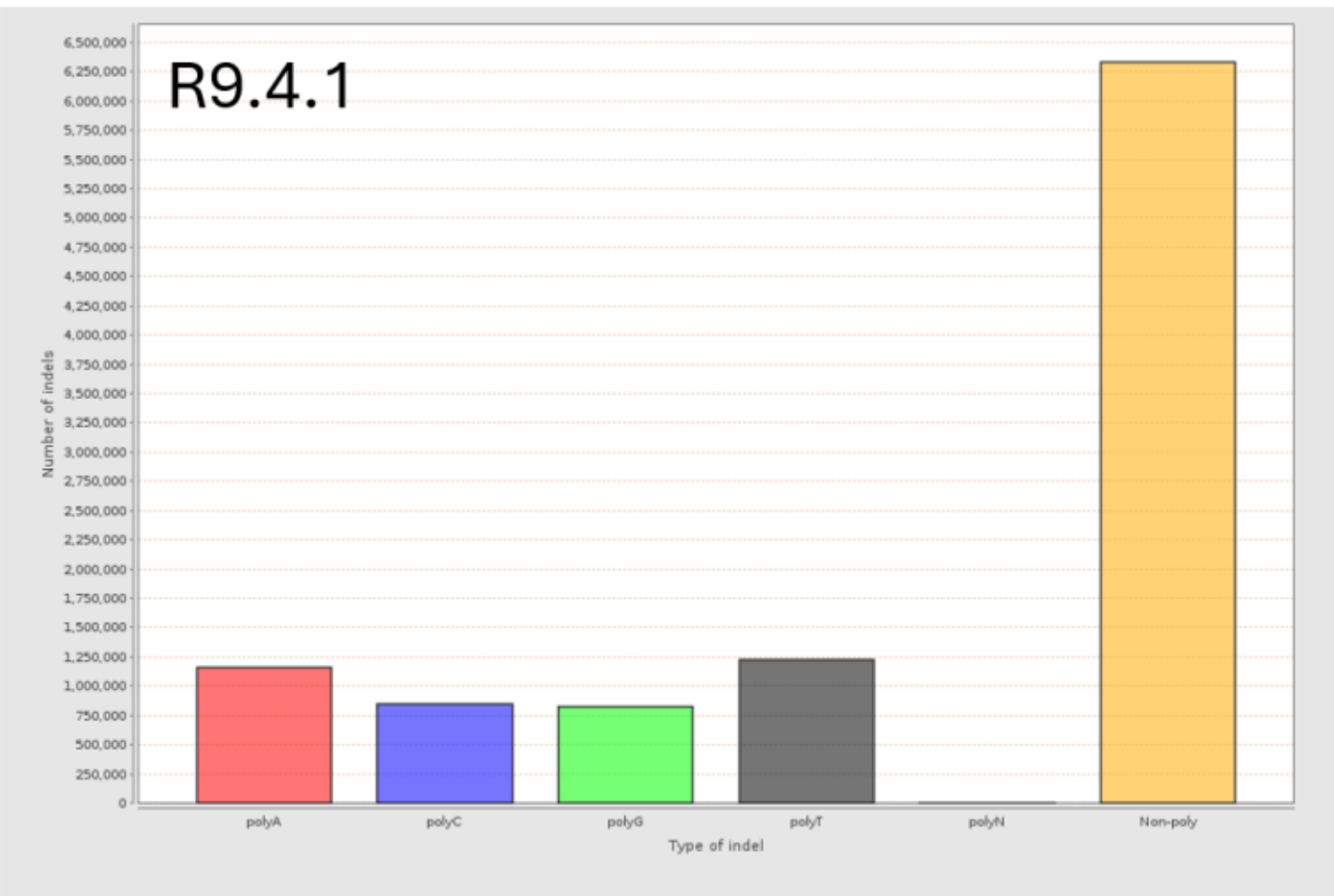

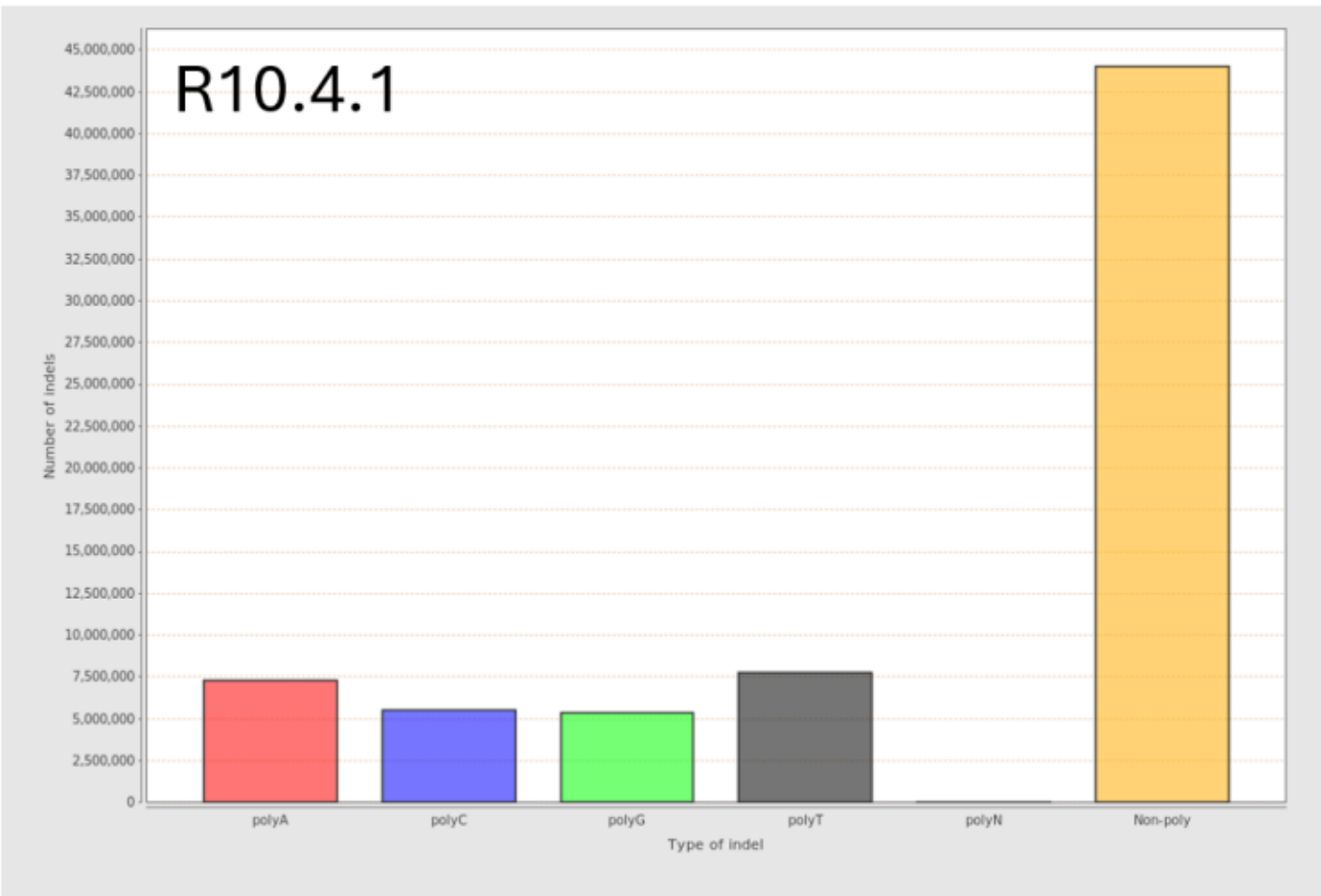

K-mer

Frequency Distribution

Correlation Matrix

k = 3

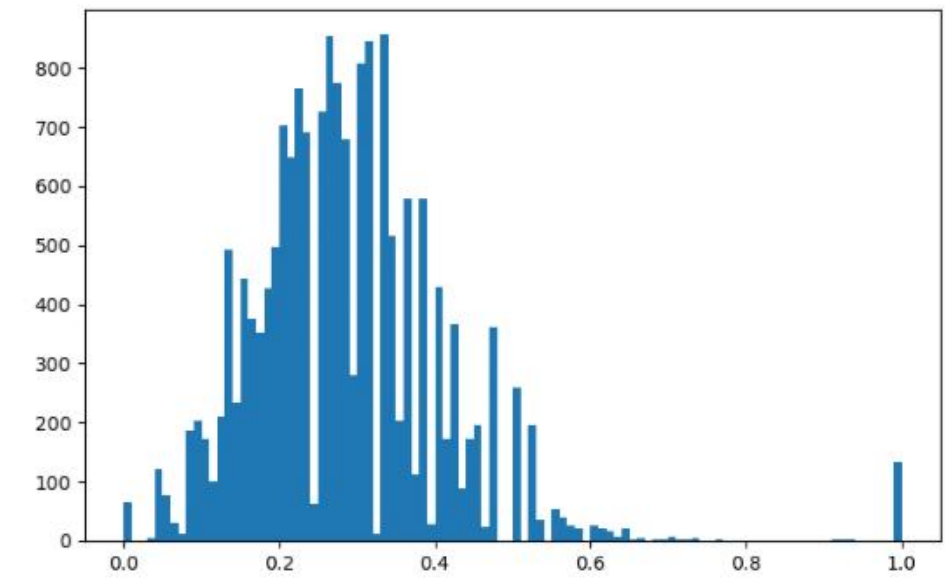

k = 3

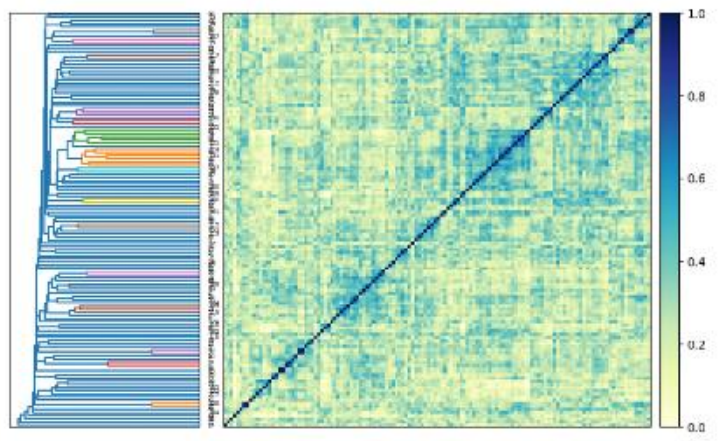

k = 4

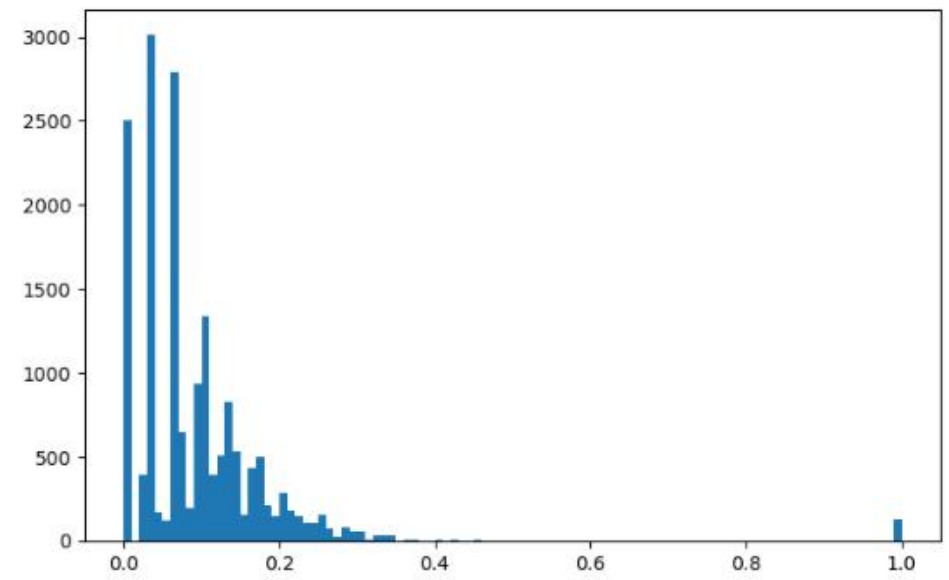

k = 4

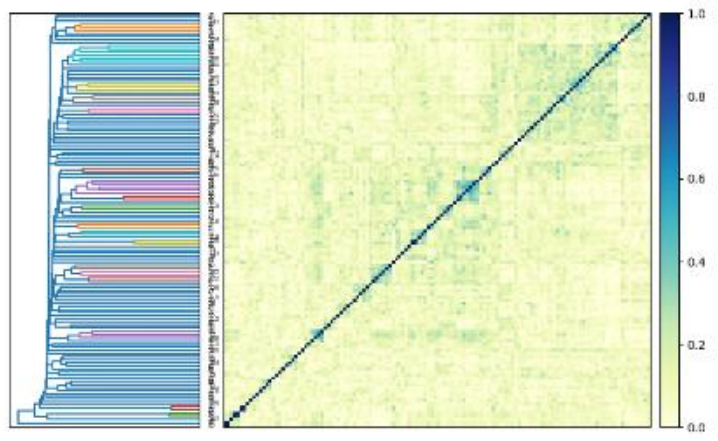

k = 5

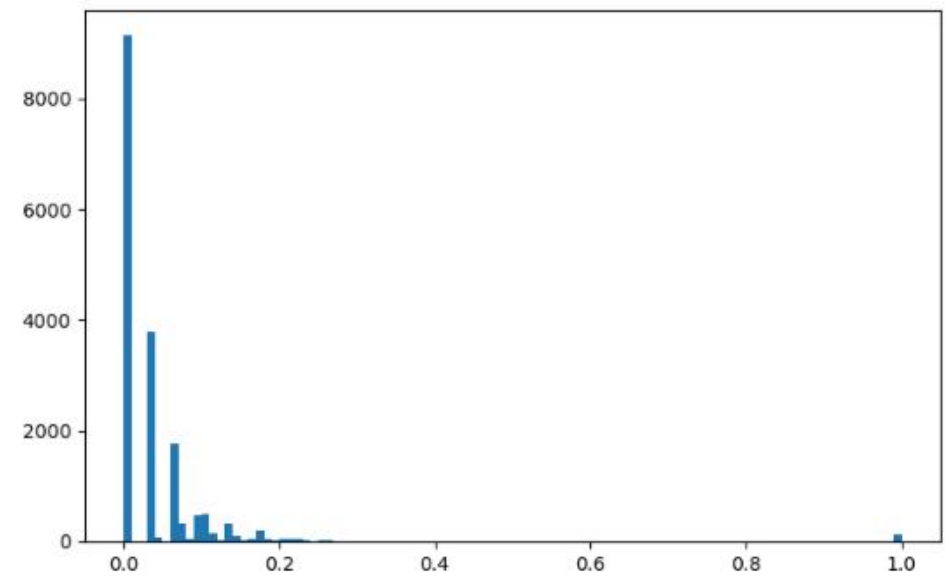

k = 5

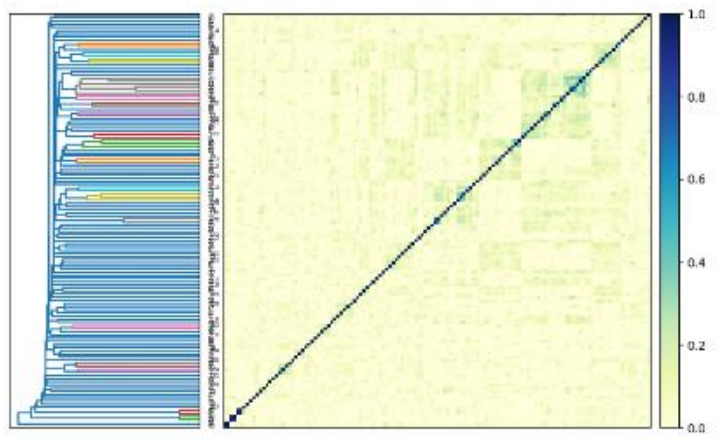

k = 6

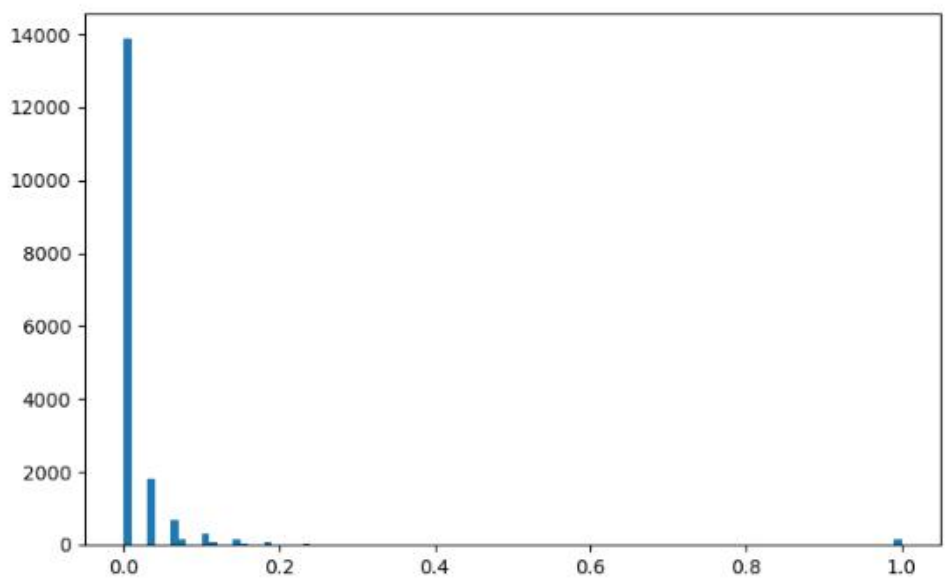

k = 6

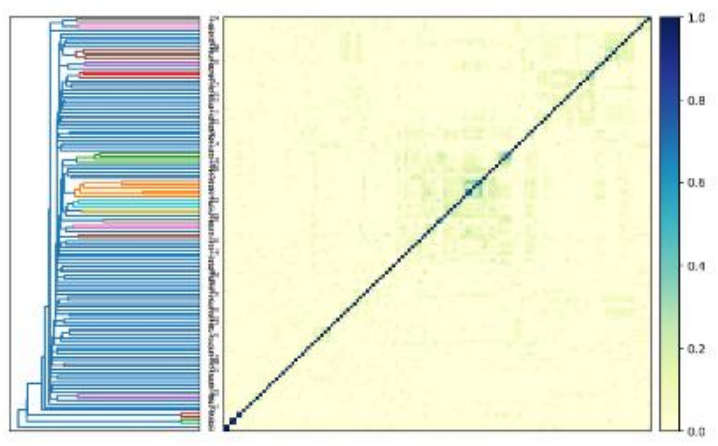

k = 7

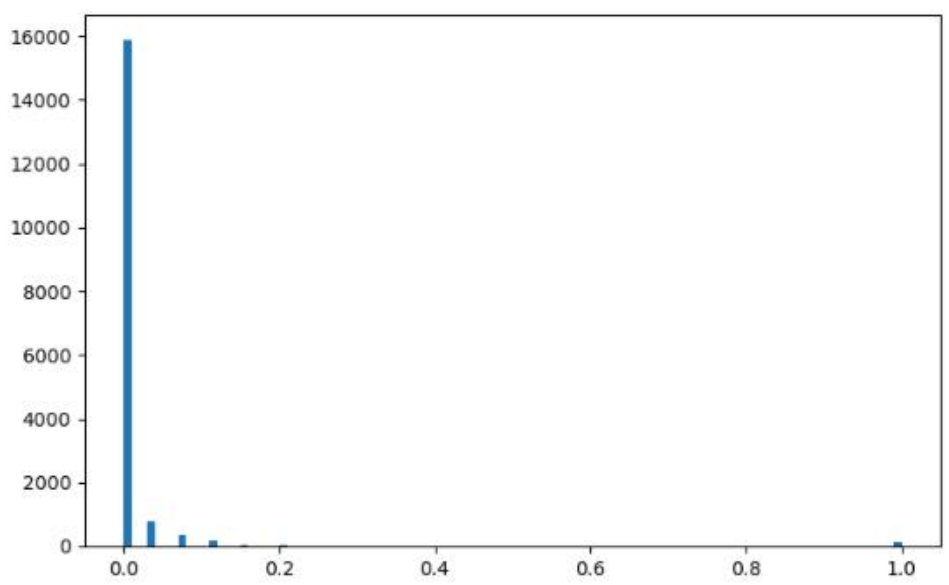

k = 7

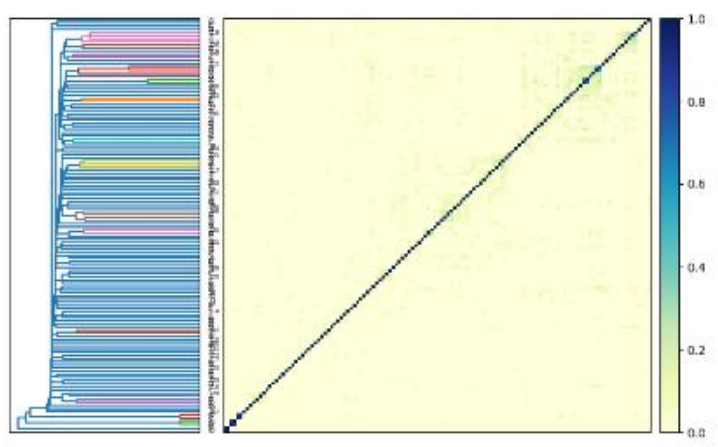

Supplement: Supplementary file 2 [file pone.0356259.s002.pdf]
