# Supplementary material for: Sub-consensus haploid variant calling in long-read sequencing technology
Source: PLoS One. 2026 Oct 5;21(10):e0356259. doi: 10.1371/journal.pone.0356259 (PMC13638048; doi:10.1371/journal.pone.0356259)
Supplement: Supplementary file 3 [file pone.0356259.s003.docx]

The paper titled “*Sub-consensus haploid variant calling in long-read sequencing technology*” by Xavier Zair evaluates the performance of LoFreq for detecting very low-frequency (sub-consensus) variants using Oxford Nanopore long-read sequencing data. The authors also introduce a calibration method that adjusts Phred quality scores to improve the accuracy of variant calling. Overall, the study is valuable and well-executed, but it would benefit from clearer explanations, better presentation of figures, and additional statistical and methodological details to enhance clarity and reproducibility.

**MAJOR COMMENTS**

1. The study only tests LoFreq. It would be stronger if the authors compared it with other current tools such as PEPPER-Margin-DeepVariant, Clair3, or Longshot2, or explained why these were not used.

2. The paper gives results for recall and false discovery rate (FDR) but does not include any statistical tests or confidence levels. The authors should add simple statistics (for example, t-tests or ANOVA) to show if the differences between flow cells or frequencies are significant

3. The part describing how Phred scores were adjusted needs clearer explanation. The authors should show how they tested the accuracy of their fitted curves (for example, by giving R² values or error plots). It should also be clear which curve fit worked best and why.

4. The paper should explain more clearly why detecting sub-consensus variants matters. For example: it can help in tracking **viral mutations** and **quasispecies**, monitoring **drug resistance**, or improving **outbreak control and vaccine design**.

5. Some details of the workflow are missing or unclear. The authors should list all software versions and random settings used.

6. The authors should confirm that all data and code mentioned in the paper are really available in the public repositories.

7. The use of both plasmid and bacterial genome experiments is interesting, but the connection between them is not clearly described. The authors should explain why both systems were chosen and how they complement each other.

8. The paper mentions 12 rounds of subsampling but does not justify why 12 was chosen. The authors should clarify if this number provides stable results or if it was an arbitrary choice.

9. The clonal plasmid data are used as a control, but there is little mention of validation with another sequencing platform (for example, Illumina or Sanger). The authors should confirm their false positive rates using an independent method for a small subset of samples

10. Figures 3–5 are difficult to interpret without reading the main text. Each figure should include a short summary line in the caption (for example, “Phred reduction of 7 eliminates false positives”).

11. The paper uses the terms *sub-consensus variants*, *rare variants*, and *low-frequency variants* in different sections.It would be better to choose one term and use it consistently throughout the text.

**MINOR COMMENTS**

1. Some **paragraphs in Methods and Results** are repetitive, especially about Phred-score analysis and flow-cell differences. These could be shortened.

2. The **figure legends** should show; sequencing depth on the x-axis, FDR or recall (%) on the y-axis, and the number of repeats (n = 12).

3. The authors must use consistent terms in **Table 1 and 2** (e.g., use either “false discovery rate” or “false positives”).

4. The authors must add full **software version numbers** (for example, QUAD v0.2, LoFreq v2.1.3.1, Rasusa v0.6.1) in the text or supplementary data.

5. In the **Abstract**, the phrase “offers a practical solution” could be more specific: for example, “offers a simple calibration approach that reduces false discovery rates in ONT data.”

6. In the **Introduction**, the first paragraph is very long; it can be split into two parts for clarity, one about NGS use and one about variant calling challenges.

7. In the **Methods**, average read lengths for R9.4.1 and R10.4.1 flow cells should be added.

8. In the **Results**, the authors note that FDR “plateaued at 25%.” It would be good to compare this value with results from earlier studies

9. In the **Discussion**, the authors must clearly list the study’s main **limitations**, such as reduced indel accuracy and limited use for diploid genomes.

10. A single figure or schematic summarizing the **workflow and the Phred calibration process** would be useful!
