# Supplementary material for: Sub-consensus haploid variant calling in long-read sequencing technology
Source: PLoS One. 2026 Oct 5;21(10):e0356259. doi: 10.1371/journal.pone.0356259 (PMC13638048; doi:10.1371/journal.pone.0356259)
Supplement: Supplementary file 4 [file pone.0356259.s004.docx]

Dear Reviewers,

We would like to sincerely thank you for the comments provided. We found all feedback to be highly constructive, and we are confident that the revisions have strengthened the manuscript considerably. We have addressed all suggestions in some form; where direct implementation was not applicable, we have provided alternative solutions that address the underlying concerns.

We hope you will find these revisions suitable. Notably, the study repository has undergone extensive updates, and we invite you to refer to <https://github.com/x-zair/ONT_truthset> for the revised README and supplementary materials.

Below, we provide point-by-point responses to each comment, along with the corresponding actions taken and line numbers where changes have been implemented (line numbers correspond to the non-markup version of the manuscript).

Thank you once again for taking the time to provide such invaluable feedback on our work. We look forward to hearing from you and hope that these revisions adequately address the points raised.

Sincerely,

Xavier Zair

Graduate Student
Saw Swee Hock School of Public Health
National University of Singapore

Reviewer #1
> The background states that this study "validates". This is more of an assessment or maybe verification, but not expansive enough to be a validation. Please correct.

Response: We agree the study is not exhaustive enough to warrant classification as a validatory study.

Action taken: Corrected to "benchmark/assess performance" instead of "validating" in relevant instances.

Location in manuscript: Lines 15 (Abstract) and 82-83 (Intro)
> Clarify novelty and context
> The rationale for applying LoFreq to ONT data is clear, but the novelty relative to prior work (e.g., Liu et al., Nat Commun 2022, Variebl tool) should be explicitly discussed.

Response: We have expanded the discussion of prior work and our contribution. The specific paper (Liu et al.) was already referenced in the introduction (reference 29) in the initial draft. We have now added more extensive discussion expanding on the differences between the methods described in that paper and our proposed methods.

Action taken: Additional context and discussion added at multiple locations throughout the manuscript:

- Lines 75-77: Introduction with reference to Liu et al
- Lines 373-384: Expanded discussion of differences between methods in benchmarking
- Lines 387-389: Differences between our proposed Phred reduction and the Variabel tool

> What new insight or methodological advance does this study provide beyond existing ONT rare-variant analyses?

> The finding that the basequality discrepancy it the nanopore datasets can be of value in efforts to apply other Illumina analysis toolkits to nanopore data. However this is speculative and we cannot with certain say that there are many avenues for this approach.

Response: We acknowledge that the primary insight this study offers is demonstrating LoFreq's applicability to ONT data with appropriate calibration. Instead, we emphasize that the dataset is publicly available for the purposes of future variant calling benchmarks. The finding that base quality discrepancy in nanopore datasets can be valuable in efforts to apply other Illumina analysis toolkits to nanopore data is noteworthy, though we recognize the need to avoid speculative statements.

Action taken: Speculative statements have been corrected to maintain appropriate tone.

Location in manuscript: Lines 439

> Benchmark against other variant callers
> Lines 153–154
Longshot and Clair3 are mentioned but not analysed. A comparative table or figure summarizing LoFreq versus these tools would strengthen claims of performance and highlight the relative advantages of LoFreq calibration.
Include additional validation metrics.

Response: An initial investigatory benchmark was performed against Longshot and Clair3. However, after corresponding with Clair3's author (Dr. Ruibang Luo), it was stressed that these alternate variant callers were not intended for haploid genomes and drastically lose sensitivity when diploid phasing is disabled. As a result, we felt including the variant identification might not represent Clair3 and Longshot accurately.

Action taken: Nevertheless, we agree the omission of this data appears incomplete. We propose the addition of this data in supplementary information. We have included the full benchmark in Supplementary Sheet 1, while also explicitly mentioning the nuance discussed above.

Location in manuscript:

- Lines 224-229: Reporting results of alternative variant callers
- Addition of Supplementary_sheet_1.pdf with complete benchmarking

> Expand methodology for Phred-score calibration
L 165–175
The QUAD v0.2 tool and curve-fitting approach require clearer description. Please specify how curves were selected or validated, whether fits were cross-validated, and whether adjustments were uniform or context-specific across bases.

Response: This is a valid suggestion. Specifics not included in the original methods have since been added.

Action taken:

- Lines 190-208 (Methods): Edited for better clarity with detailed description of calibration methodology
- Study repository updated with comprehensive details on curve fitting and validation

> L 179–192
> Only recall and FDR are reported. Please add precision, F1-score, and ROC/AUC values, or justify their omission. This will improve the quantitative robustness of the benchmarking.

Response: Thank you for the suggestion We do agree the initial manuscript provided insufficient statistical analysis. However, we have several concerns about adding these metrics:
**1. Our stratified design makes aggregate metrics uninformative.** We intentionally stratified variants by allele frequency (5%, 10%, 15%, 19%) to assess frequency-dependent recall. Aggregate F1-scores would collapse this stratification, obscuring our key finding that calibration differentially affects low-frequency variants (Table 1: -8 Phred maintains 100% recall at ≥10% AF but drops to 10.5% at 5% AF). A single F1-score cannot convey performance trade-offs across the allele frequency spectrum.

**2. Precision is artificially inflated by overwhelming true negatives.** With 100 variants in 7,179 bp plasmids (or 4.6 Mb genomes), precision calculations include ~7,000-4,600,000 true negative positions. Even with high false positive rates (e.g., 50 FP = 33% FDR), precision remains 98.6-100%, masking the actual error burden. FDR directly quantifies what matters to users: of reported variants, what proportion are errors (e.g., 12.88%).

**3. Stratified recall + FDR provides actionable information.** Our Table 1 presentation shows sensitivity across frequency bins and false positive rates simultaneously—trade-offs that aggregate metrics obscure. This approach aligns with variant calling benchmarking best practices (Krusche et al., Nat Biotechnol 2019; GIAB Consortium).

**4. ROC/AUC is inappropriate for our design.** LoFreq produces binary variant calls without continuous probability scores. Our Phred reductions represent distinct calibration strategies, not threshold variations on the same model.

Action taken: We have maintained recall and FDR for Table 1 as these are most informative for our stratified design. However, we have changed SD values to 95% confidence intervals as suggested by Reviewer 2.

Additionally, we have added precision and F1-scores to Table 2, where we compare different calibration methods (polynomial fits vs. scalar reductions) on the same variant set. In this context, F1-scores appropriately quantify the overall performance of each calibration approach and facilitate direct method comparison. This demonstrates that scalar Phred reduction (F1 = 78.1% at -3) outperforms polynomial calibration methods. We have also added more statistical tests, explained below.

> Statistical analysis of replicates
> L 182–188
> Mean ± SD are provided, but no statistical testing (e.g., t-test or ANOVA) is shown to support differences between chemistries or sequencing depths. Incorporating these analyses would add rigor.

Response: We have added comprehensive statistical testing throughout the manuscript.

Actions taken:

1. **Chemistry comparisons** (R9.4.1 vs R10.4.1):
Unpaired two-sample t-tests added to Results for both mixed and clonal plasmids.

2. **Phred reduction effects:**
Linear regression quantifying Phred reduction- FDR relationship in plasmids

3. **Effect size analysis:**
Cohen's d added to demonstrate practical significance in genome datasets

> Interpretation of FDR patterns
> L 273–286
> The discussion attributes FDR differences mainly to Phred-score biases and BAQ limitations. Please consider other contributing factors such as strand bias, homopolymer effects, or systematic basecaller errors. Another consider is the fact that SARS-CoV-2 genome segments used in this study are produced from diploid plasmid DNA and not RNA template involving cDNA as starting material. Granted these are potential for lab errors and not sequencing, but the reader should be made aware of the other issues in translating this approach to RNA virus analysis. See also Hannah Yu et al., Advanced Science 2025.

Response:
Regarding other factors- Before scalar reduction was decided one, we looked at context-specific removal of errors based on homopolymer or k-mer patterns very briefly. However, we decided not to pursue this method of filtering as we did not managed to find any sequence specific patterns in the miscalled variants and their flanking motifs. Upon noticing scalar reductions work most effectively, the study is unable to comment deeply on the other factors.

However, we have Added Supplementary_sheet_2 – with our exploratory homopolymer and k-mer signature analysis on clonal bacterial genome datasets.

Strand bias: Strand bias was not a factor we investigated in detail and we don't have insights into this. This has been declared in the discussion.

Lines 379-384: Acknowledgment of strand bias limitations

RNA vs DNA libraries: Generation of libraries from double-stranded plasmid DNA is indeed not representative of RNA virus libraries, thank you for bringing this point up.

Lines 378-379: Discussion of RT-PCR artifacts and limitations when translating to RNA virus analysis

Minor Comments
> Formatting and consistency
> L 35: “At of the time” → “At the time.”
> L 121: “ONTs ligation” → “ONT’s ligation.”
> Ensure consistent capitalization of LoFreq throughout.
> Grammar and readability
> Several long sentences in the Introduction (Lines 41–44) could be split for clarity.
> Use past tense for results, present tense for interpretations.

Action taken: All minor formatting, grammatical, and consistency issues have been corrected throughout the manuscript. Long sentences have been split for improved clarity. Tense usage has been standardized (past tense for results, present tense for interpretations).

> Figures and tables
Tables 1 and 2 should report replicate numbers and confidence intervals.

Action taken: Table 1 have been changed to reflect suggested changes. Table 2 does not have replicate numbers as the intention was to identify the best calibration method, however more descriptive stats such as precision, F1 and stratification by frequency has been added.

Reviewer #2:
MAJOR COMMENTS

1. The study only tests LoFreq. It would be stronger if the authors compared it with other current tools such as PEPPER-Margin-DeepVariant, Clair3, or Longshot2, or explained why these were not used.

Response: Initial validation was performed on Longshot and Clair3. However, it was mentioned by Clair3's author (Dr. Ruibang Luo) that these alternate variant callers were not intended for haploid genomes and drastically lose sensitivity when diploid phasing is disabled. Although PEPPER was not tested, as a haplotype-aware variant caller, we also expect performance to be similar.

Action taken: As mentioned by both reviewers, we have included the benchmarks in the supplementary data (Supplementary Sheet 1), while explicitly mentioning use case, so as not to misrepresent the tested variant callers.

Location in manuscript:

-	Lines 224-229: Reporting results of alternative variant callers

-	Addition of Supplementary_sheet_1.pdf with complete benchmarking

2. The paper gives results for recall and false discovery rate (FDR) but does not include any statistical tests or confidence levels. The authors should add simple statistics (for example, t-tests or ANOVA) to show if the differences between flow cells or frequencies are significant

Action taken: 95% confidence intervals have replaced SD calculations. Paired t-test results are reported for the differences between flow cell chemistries.

Regression and Cohens D performed for Phred reduction metrics.

Location in manuscript: Throughout Tables 1 and 2, and Results section

3. The part describing how Phred scores were adjusted needs clearer explanation. The authors should show how they tested the accuracy of their fitted curves (for example, by giving R² values or error plots). It should also be clear which curve fit worked best and why.

Response: Thank you for the suggestion. On review, we agree the calibration details were not comprehensive enough.

Action taken:

- Lines 190-208 (Methods): The entire section has been overhauled to include detailed description of the curve fittings, including validation metrics
- Lines 414-431 (Discussion): The best approach has been more clearly emphasized
- Study repository has been overhauled to include detailed code, workflow information and intermediate files.

4. The paper should explain more clearly why detecting sub-consensus variants matters. For example: it can help in tracking viral mutations and quasispecies, monitoring drug resistance, or improving outbreak control and vaccine design.

Action taken: Section has been added with more detail in the discussion.

Location in manuscript: Lines 353-364

5. Some details of the workflow are missing or unclear. The authors should list all software versions and random settings used.

Response and action taken: Thank you for bringing this point up. More details of the workflow have been added to the manuscript. The study repository on GitHub has also undergone substantial overhaul with very detailed information on the workflow and software used, including version numbers.

6. The authors should confirm that all data and code mentioned in the paper are really available in the public repositories.

Action taken: This has been verified. More details added to study repository with comprehensive documentation.

7. The use of both plasmid and bacterial genome experiments is interesting, but the connection between them is not clearly described. The authors should explain why both systems were chosen and how they complement each other.

Action taken: This has been emphasized further in additions.

Location in manuscript: Lines 115-118

8. The paper mentions 12 rounds of subsampling but does not justify why 12 was chosen. The authors should clarify if this number provides stable results or if it was an arbitrary choice.

Action taken: Clarified with justifications, some minor stats as well as industry practices have been cited in the methods.

Location in manuscript: Lines 159-162

9. The clonal plasmid data are used as a control, but there is little mention of validation with another sequencing platform (for example, Illumina or Sanger). The authors should confirm their false positive rates using an independent method for a small subset of samples

Response and action taken: The identity of all plasmid templates was verified with Sanger sequencing to ensure sequence integrity. As it was not initially mentioned in the manuscript, the Sanger reads have since been uploaded to the study repository.

Location in manuscript: Lines 116

10. Figures 3–5 are difficult to interpret without reading the main text. Each figure should include a short summary line in the caption (for example, “Phred reduction of 7 eliminates false positives”).

Action taken: All figure captions have been enhanced with summary statements.

11. The paper uses the terms sub-consensus variants, rare variants, and low-frequency variants in different sections. It would be better to choose one term and use it consistently throughout the text.

Action taken: This has been standardized to "sub-consensus variants" where previously ambiguous.

MINOR COMMENTS

1. Some paragraphs in Methods and Results are repetitive, especially about Phred-score analysis and flow-cell differences. These could be shortened.

Action taken: Repetitive elements removed where appropriate.

2. The figure legends should show; sequencing depth on the x-axis, FDR or recall (%) on the y-axis, and the number of repeats (n = 12).

Action taken: Missing aspects addressed in new figure descriptions.

3. The authors must use consistent terms in Table 1 and 2 (e.g., use either “false discovery rate” or “false positives”).

Action taken: Corrected in both tables and standardized throughout the manuscript. We refer to false positive frequencies as raw counts of false positives, while FDR is expressed as a percentage.

4. The authors must add full software version numbers (for example, QUAD v0.2, LoFreq v2.1.3.1, Rasusa v0.6.1) in the text or supplementary data.

Action taken: Verified and further additions made to the manuscript and study repository.

5. In the Abstract, the phrase “offers a practical solution” could be more specific: for example, “offers a simple calibration approach that reduces false discovery rates in ONT data.”

Action taken: Corrected, also in response to Reviewer 1.

Location in manuscript: Lines 31 (abstract) Lines 489 (discussion)

6. In the Introduction, the first paragraph is very long; it can be split into two parts for clarity, one about NGS use and one about variant calling challenges.

Action taken: Adjusted, with a split in the paragraph and minor rephrasing.

Location in manuscript: Lines 34-48 (Introduction)

7. In the Methods, average read lengths for R9.4.1 and R10.4.1 flow cells should be added.

Action taken: Average read lengths have been added to the Methods section.

Location in manuscript: Lines 143 (Methods)

8. In the Results, the authors note that FDR “plateaued at 25%.” It would be good to compare this value with results from earlier studies

Action taken: Comparison with earlier studies has been added where relevant literature is available.

Location in manuscript: Lines 387 (Methods)

9. In the Discussion, the authors must clearly list the study’s main limitations, such as reduced indel accuracy and limited use for diploid genomes.

Action taken: A comprehensive limitations section has been added to the Discussion, clearly outlining reduced indel accuracy and limited applicability to diploid genomes, among other constraints.

Location in manuscript: Lines 414-431 (Methods)

10. A single figure or schematic summarizing the workflow and the Phred calibration process would be useful!

Action taken: We welcome this suggestions, however for fears of having too many figures on the main body of the manuscript, we have included a detailed workflow in the study repository.
