# Supplementary material for: Sub-consensus haploid variant calling in long-read sequencing technology
Source: PLoS One. 2026 Oct 5;21(10):e0356259. doi: 10.1371/journal.pone.0356259 (PMC13638048; doi:10.1371/journal.pone.0356259)
Supplement: Supplementary file 5 [file pone.0356259.s005.docx]

The authors have made substantial improvements in response to the first-round review. The revised manuscript is clearer, contains expanded methodological detail (including software versions), provides a statistical analysis section, and better motivates the relevance of sub-consensus/low-frequency variant detection. The additions regarding Phred score adjustment and model-fit evaluation are helpful and improve interpretability.

However, a small number of issues remain that affect internal consistency and reproducibility. These are largely editorial/clarificatory and can be addressed quickly. I therefore recommend **Minor Revision**.

**COMMENTS**

1. The Statistical Analysis section states confidence intervals are calculated with *n=3 replicates per condition (df=2)*, yet Methods and Results repeatedly refer to *12 rounds/replicates* and figures/tables state *(n=12)*. The authors should define precisely what constitutes a “replicate” versus a “subsample/iteration” (e.g., independent libraries/flow cells vs computational subsampling replicates), then make all *n* values consistent across Methods, Results, Tables/Figures, and CI calculations. If CIs in Tables are based on n=12, the Statistical Analysis text must reflect that (and vice versa)..
2. The authors should clarify whether subsampling was performed with a fixed random seed. If yes, they have to report the seed (or where it is set in the pipeline). If not, they must state that subsampling is stochastic and either (a) justify why this does not affect conclusions or (b) report variability across runs.
3. The revision appropriately discusses limitations for indel calling and low allele fractions. However, the manuscript should include a clear, explicit statement regarding the intended scope (haploid/sub-consensus contexts) and that the approach is not validated for diploid germline variant calling, e.g., “*This approach is designed and validated for haploid sub-consensus variant detection and has not been evaluated for diploid germline variant calling*.” etc..
4. The Results include a brief comparison and note that Longshot/Clair3 are not designed for sub-consensus haploid calling. The authors should add 2–3 sentences in the main Discussion clarifying (i) why broader ONT variant-caller benchmarking is limited for this use case and (ii) what conclusions can and cannot be drawn from the supplementary comparison.
5. The authors must standardize terminology across the manuscript (choose one primary term such as ‘sub-consensus variants’ or ‘low-frequency variants’) and use alternative terms only once as synonyms to avoid confusion.
6. While effect sizes (Cohen’s d) are now mentioned in Methods, Results still report SD in places, while other sections refer to CI.

The authors need to

- use a single reporting standard (either SD or 95% CI) consistently across Results, Tables, and Figures;
- report effect sizes alongside p-values wherever inferential tests are used;
- add one brief sentence confirming that test assumptions were checked (or state which nonparametric alternatives were used if assumptions were not met); and
- ensure the paired vs unpaired tests match the study design and that the reported degrees of freedom are consistent with the final sample size definition.
