# Supplementary material for: Sub-consensus haploid variant calling in long-read sequencing technology
Source: PLoS One. 2026 Oct 5;21(10):e0356259. doi: 10.1371/journal.pone.0356259 (PMC13638048; doi:10.1371/journal.pone.0356259)
Supplement: Supplementary file 6 [file pone.0356259.s006.docx]

Dear reviewers,

We would like to start by once again expressing our sincere thanks for taking the time to read and address areas of improvement in our manuscript. We find the comments provided during the second review to be as constructive as the last review and we are happy to address the points mention.

We are pleased that the manuscript was received positively overall, and we believe the suggested addition has overall strengthen the interpretability and general quality of the study. We hope that you find the revisions to be sufficient and we look forward to hearing your conclusions.

Please find a point-by-point response to the remaining issues raised, the line locations mentioned are made with reference to the ‘tracked changes accepted’ version of the manuscript.

It has been a pleasure corresponding with you, and we look forward to hearing your decision on the manuscript.

Sincerely,
Xavier Zair

Graduate Student
Saw Swee Hock School of Public Health
National University of Singapore

1. The Statistical Analysis section states confidence intervals are calculated with *n=3 replicates per condition (df=2)*, yet Methods and Results repeatedly refer to *12 rounds/replicates* and figures/tables state *(n=12)*. The authors should define precisely what constitutes a “replicate” versus a “subsample/iteration” (e.g., independent libraries/flow cells vs computational subsampling replicates), then make all *n* values consistent across Methods, Results, Tables/Figures, and CI calculations. If CIs in Tables are based on n=12, the Statistical Analysis text must reflect that (and vice versa)..

*We would like to thank the reviewers for identifying this inconsistency. The confusion arose because two distinct sample populations are used across our analyses, each with a different n, and this distinction was not clearly stated in the original Statistical Analysis section.*

*The n=3 refers exclusively to the three independent clonal plasmid libraries (Delta, wild-type, and Omicron), which were not subsampled and serve solely to characterise baseline sequencing error in variant-free backgrounds. The n=12 refers to computational subsampling replicates applied to all mixed plasmid and genomic datasets.*

*Sentence added after “Descriptive statistics are reported as means with 95% confidence intervals (CI), calculated using the t-distribution”:*

`:n=12 subsampling replicates (df=11, t-critical=2.201) for mixed and genomic datasets, and n=3 independent clonal libraries (df=2, t-critical=4.303) for error characterisation in variant-free datasets (Delta, wild-type, and Omicron). Subsampling replicates represent computational draws rather than independent library preparations; reported CIs therefore reflect depth-normalised sampling variability.`

*Lines 223-226*

2. The authors should clarify whether subsampling was performed with a fixed random seed. If yes, they have to report the seed (or where it is set in the pipeline). If not, they must state that subsampling is stochastic and either (a) justify why this does not affect conclusions or (b) report variability across runs.

*No fixed random seed was applied; subsampling was stochastic across iterations. This is now explicitly stated in the Methods section, along with an insertion that run-to-run variability is captured in the 95% CIs.*

*Sentence added:*

*`* No fixed random seed was applied; subsampling was therefore stochastic across iterations, with run-to-run variability captured by the 95% confidence intervals.*`*

*Lines 162-163*

3. The revision appropriately discusses limitations for indel calling and low allele fractions. However, the manuscript should include a clear, explicit statement regarding the intended scope (haploid/sub-consensus contexts) and that the approach is not validated for diploid germline variant calling, e.g., “*This approach is designed and validated for haploid sub-consensus variant detection and has not been evaluated for diploid germline variant calling*.” etc..

*We agree that it should be emphasised that the methods and results discussed in this study is not for diploid applications. However, rather than adding a caveat to the Discussion, we addressed this more robustly in the Introduction by explicitly acknowledging that diploid germline variant calling is already well-served by dedicated tools, thereby drawing the scope boundary naturally without requiring a defensive disclaimer elsewhere.*

*Original Introduction “Although dozens of variant identification tools exist, many are optimized for diploid germline or tailored for somatic mutation calling, suboptimal for populational level variant analysis” modified to:*

*`* Diploid germline variant calling is well-served by dedicated tools such as Clair3 [49] and DeepVariant [54], while Longshot [48] targets long-read diploid SNP calling specifically — none of which are suited for population-level sub-consensus variant analysis in haploid genomes. Even viral-specific callers such as iVar [55], designed for amplicon-based intrahost diversity measurement, rely on cross-replicate concordance to filter false positives, an assumption that inherently misclassifies unique variants as noise. *`*

*Lines 67-70*

4. The Results include a brief comparison and note that Longshot/Clair3 are not designed for sub-consensus haploid calling. The authors should add 2–3 sentences in the main Discussion clarifying (i) why broader ONT variant-caller benchmarking is limited for this use case and (ii) what conclusions can and cannot be drawn from the supplementary comparison.

*Two to three sentences have been added to the Discussion paragraph addressing Longshot and Clair3.*

*Brief section added:*

*`* Longshot and Clair3 were included as contextual reference points; their recall rates in Supplementary File 1 reflect a fundamental design mismatch rather than overall caller quality. Longshot relies on haplotype-aware phasing to validate variants. When deactivated in haploid contexts, this renders the caller overly conservative, explaining the dramatic reduction in sensitivity we observed. On the other hand, Clair3's diploid allele frequency assumptions similarly filter variants below ~25% AF as unlikely to be genuine, a threshold incompatible with sub-consensus haploid detection. Dedicated benchmarking of ONT sub-consensus callers remains limited by the scarcity of validated haploid truth sets at defined allele frequencies - a gap this study's publicly released datasets are intended to help fill.*`*

*Lines 389-397*

5. The authors must standardize terminology across the manuscript (choose one primary term such as ‘sub-consensus variants’ or ‘low-frequency variants’) and use alternative terms only once as synonyms to avoid confusion.

*2 additional instances of alternate terminology in the abstract and introduction has been corrected to the standardised reference of ‘sub-consensus’ variants.*

*“but accurate low-frequency variant” 🡪 `* but accurate sub-consensus variant *`*

*Line 14*

*“Investigating minor” 🡪 `* investigating sub-consensus variants *`*

*Line 44*

6. While effect sizes (Cohen’s d) are now mentioned in Methods, Results still report SD in places, while other sections refer to CI.

The authors need to

- use a single reporting standard (either SD or 95% CI) consistently across Results, Tables, and Figures;

Instances of SD have been recalculated to 95% CI intervals instead.

“ (SD 1.48)” 🡪 `(95% CI: 7.31–9.19)`

“(SD 1.04)” 🡪 `(95% CI: 1.34–2.66)`

“(SD 1.48)” 🡪 `(95% CI: 7.73–9.61)`

“(SD 1.08)” 🡪 `(95% CI: 4.19–5.57)`

Lines 246-252

- report effect sizes alongside p-values wherever inferential tests are used;

Effect sizes have been added where previously absent — Cohen's d effect sizes are now reported alongside all inferential test results.

“(unpaired t-test, t = 6.9, df = 22, p < 0.001).” 🡪 `(unpaired t-test, t = 12.4, df = 22, p < 0.001, Cohen's d = 4.89)`

“(unpaired t-test, t = 12.4, df = 22, p < 0.001)” 🡪 `(unpaired t-test, t = 6.9, df = 22, p < 0.001, Cohen's d = 2.93)`

Lines 246-252

- add one brief sentence confirming that test assumptions were checked (or state which nonparametric alternatives were used if assumptions were not met); and

Regarding test assumptions: parametric methods were selected based on the nature of our data and initial visual inspection of distributions. Following reviewer's comment, we formally verified normality using the Shapiro-Wilk test across all primary outcome variables (W > 0.90, p > 0.05). All groups passed, confirming the appropriateness of parametric inference throughout. This has been stated explicitly in the revised Statistical Analysis section.

Sentence added:

`Parametric statistical methods were used throughout following confirmation of data normality via Shapiro-Wilk testing (*W* > 0.90, *p* > 0.05).`

Lines 213-215

- ensure the paired vs unpaired tests match the study design and that the reported degrees of freedom are consistent with the final sample size definition.

The Statistical Analysis section has been updated to explicitly distinguish between unpaired t-tests for between-chemistry comparisons and paired t-tests for within-chemistry Phred reduction comparisons in bacterial genomic datasets.
Degrees of freedom were also verified.

Sentence added after “Differences in false discovery rates and recall between flow cell chemistries (R9.4.1 vs R10.4.1) and across sequencing depths were assessed using unpaired two-sample t-tests”:

` Paired t-tests were applied for within-chemistry Phred reduction comparisons in bacterial genomic datasets. `

Lines 215-217

Line 253. Uses allele frequency 0.1, 0.15, 0.19. However Table 1 uses 5%, 10%, 15%, 19%. Pick one for consistency and ease of understanding.

*All measures of allele frequency has been standardised to percentages. Other occurrences including Lines 253 have been rectified:*

*Lines 130-131,
Line 252
Lines 268-271
Lines 337-338
Lines 341-343*

Figure 3b, emphyrical was used on the Y-axis instead of empirical

*Typo has been corrected, updates to Figure 3.*

*Other changes made:*

*Introduction: Two new references added — DeepVariant [54] and iVar [55] — to support the expanded caller landscape discussion.*

*Lines 68-69*

*Results: Absolute variant counts added alongside percentages to improve interpretability to Longshot and Clair3 preliminary analysis.*

*`*LoFreq detected 759 total variant calls across all barcodes, achieving a recall of 84.33% (141 variants missed out of 900 expected). Longshot identified 211 calls with a recall of 23.44% (689 variants missed), while Clair3 made 305 calls with a recall of 33.89% (595 variants missed). LoFreq demonstrated superior sensitivity across the tested allele frequency range, particularly at sub-consensus frequencies (<20%), recovering variants that both Longshot and Clair3 failed to detect*`*

*Lines 233-235*

*Table 1: An error was identified in the initial tabulation. Intermediate CSV extraction introduced rounding artefacts that propagated into the reported values. All values have been recomputed directly from raw VCF files against the validated plasmid truth set (n=12, df=11, t-critical=2.201 throughout). Raw counts and the tabulation script have been deposited in the study repository (*[*https://github.com/x-zair/ONT_truthset/tree/main/summary_statistics*](https://github.com/x-zair/ONT_truthset/tree/main/summary_statistics) *as* *table1_raw_plasmid_var_counts.xlsx and vcf_parser_script.py).*

*Discussion: The BAQ paragraph has been expanded with additional detail on the computational limitations encountered during this study.*

*`* In practice, a single 4 Mb reference alignment at 50× depth failed to complete BAQ computation even after one week of continuous runtime on 70 threads, precluding its use in our genomic datasets. *`*

*Lines 421-423.*
