# Supplementary material for: Sub-consensus haploid variant calling in long-read sequencing technology
Source: PLoS One. 2026 Oct 5;21(10):e0356259. doi: 10.1371/journal.pone.0356259 (PMC13638048; doi:10.1371/journal.pone.0356259)
Supplement: Peer review history

# Peer review: Decision Letter 0

**DOI:** 10.1371/journal.pone.0356259.r001

**Authors:** Nihad A.M Al-Rashedi

28 Oct 2025

Dear Dr. Zair,

Please submit your revised manuscript by Dec 12 2025 11:59PM. If you will need more time than this to complete your revisions, please reply to this message or contact the journal office at plosone@plos.org. When you're ready to submit your revision, log on to https://www.editorialmanager.com/pone/ and select the 'Submissions Needing Revision' folder to locate your manuscript file.

- A rebuttal letter that responds to each point raised by the academic editor and reviewer(s). You should upload this letter as a separate file labeled 'Response to Reviewers'.
- A marked-up copy of your manuscript that highlights changes made to the original version. You should upload this as a separate file labeled 'Revised Manuscript with Track Changes'.
- An unmarked version of your revised paper without tracked changes. You should upload this as a separate file labeled 'Manuscript'.

If you would like to make changes to your financial disclosure, please include your updated statement in your cover letter. Guidelines for resubmitting your figure files are available below the reviewer comments at the end of this letter.

If applicable, we recommend that you deposit your laboratory protocols in protocols.io to enhance the reproducibility of your results. Protocols.io assigns your protocol its own identifier (DOI) so that it can be cited independently in the future. For instructions see: https://journals.plos.org/plosone/s/submission-guidelines#loc-laboratory-protocols. Additionally, PLOS ONE offers an option for publishing peer-reviewed Lab Protocol articles, which describe protocols hosted on protocols.io. Read more information on sharing protocols at https://plos.org/protocols?utm_medium=editorial-email&utm_source=authorletters&utm_campaign=protocols.

We look forward to receiving your revised manuscript.

Kind regards,

Nihad A.M Al-Rashedi

Academic Editor

PLOS ONE

Journal requirements:

When submitting your revision, we need you to address these additional requirements.

1. Please ensure that your manuscript meets PLOS ONE's style requirements, including those for file naming. The PLOS ONE style templates can be found at

https://journals.plos.org/plosone/s/file?id=wjVg/PLOSOne_formatting_sample_main_body.pdf and

https://journals.plos.org/plosone/s/file?id=ba62/PLOSOne_formatting_sample_title_authors_affiliations.pdf

2. Please include your tables as part of your main manuscript and remove the individual files. Please note that supplementary tables (should remain/ be uploaded) as separate "supporting information" files.

3. We notice that your supplementary information is included in the manuscript file. Please remove them and upload them with the file type 'Supporting Information'. Please ensure that each Supporting Information file has a legend listed in the manuscript after the references list.

If the reviewer comments include a recommendation to cite specific previously published works, please review and evaluate these publications to determine whether they are relevant and should be cited. There is no requirement to cite these works unless the editor has indicated otherwise.

[Note: HTML markup is below. Please do not edit.]

Reviewers' comments:

Reviewer's Responses to Questions

**Comments to the Author**

1. Is the manuscript technically sound, and do the data support the conclusions?

Reviewer #1: Partly

Reviewer #2: Partly

**********

2. Has the statistical analysis been performed appropriately and rigorously?

Reviewer #1: No

Reviewer #2: Yes

**********

3. Have the authors made all data underlying the findings in their manuscript fully available?

The PLOS Data policy

Reviewer #1: Yes

Reviewer #2: No

**********

4. Is the manuscript presented in an intelligible fashion and written in standard English?

Reviewer #1: Yes

Reviewer #2: Yes

**********

Reviewer #1: This manuscript evaluates the performance of the short-read variant caller LoFreq for detecting low-frequency (sub-consensus) variants in Oxford Nanopore Technologies (ONT) long-read datasets. Using curated plasmid and E. coli truth sets, the authors assess LoFreq’s sensitivity, false discovery rate (FDR), and introduce a Phred quality-score calibration method to reduce false positives. The work addresses a meaningful gap in the field of long-read variant analysis and presents a practical strategy for improving rare-variant detection in ONT data.

The study is technically well executed, but requires additional validation metrics, clearer comparison with alternative callers, and minor language and formatting revisions. With these improvements, the manuscript would make a valuable contribution to the field.

Major Comments

Abstract

The background states that this study "validates". This is more of an assessment or maybe verification, but not expansive enough to be a validation. Please correct.

Clarify novelty and context

Introduction

The rationale for applying LoFreq to ONT data is clear, but the novelty relative to prior work (e.g., Liu et al., Nat Commun 2022, Variebl tool) should be explicitly discussed. What new insight or methodological advance does this study provide beyond existing ONT rare-variant analyses?

Benchmark against other variant callers

Lines 153–154

Longshot and Clair3 are mentioned but not analyzed. A comparative table or figure summarizing LoFreq versus these tools would strengthen claims of performance and highlight the relative advantages of LoFreq calibration.

Include additional validation metrics.

Expand methodology for Phred-score calibration

L 165–175

The QUAD v0.2 tool and curve-fitting approach require clearer description. Please specify how curves were selected or validated, whether fits were cross-validated, and whether adjustments were uniform or context-specific across bases.

Results

L 179–192

Only recall and FDR are reported. Please add precision, F1-score, and ROC/AUC values, or justify their omission. This will improve the quantitative robustness of the benchmarking.

Statistical analysis of replicates

L 182–188

Mean ± SD are provided, but no statistical testing (e.g., t-test or ANOVA) is shown to support differences between chemistries or sequencing depths. Incorporating these analyses would add rigor.

Interpretation of FDR patterns

L 273–286

The discussion attributes FDR differences mainly to Phred-score biases and BAQ limitations. Please consider other contributing factors such as strand bias, homopolymer effects, or systematic basecaller errors. Another consider is the fact that SARS-CoV-2 genome segments used in this study are produced from diploid plasmid DNA and not RNA template involving cDNA as starting material. Granted these are potential for lab errors and not sequencing, but the reader should be made aware of the other issues in translating this approach to RNA virus analysis. See also Hannah Yu et al., Advanced Science 2025. https://advanced.onlinelibrary.wiley.com/doi/pdf/10.1002/advs.202505978

Minor Comments

Formatting and consistency

L 35: “At of the time” → “At the time.”

L 121: “ONTs ligation” → “ONT’s ligation.”

Ensure consistent capitalization of LoFreq throughout.

Grammar and readability

Several long sentences in the Introduction (Lines 41–44) could be split for clarity.

Use past tense for results, present tense for interpretations.

Figures and tables

Tables 1 and 2 should report replicate numbers and confidence intervals.

Data availability

The inclusion of the SRA and GitHub repositories is commendable. Please verify that all scripts and calibration CSVs are accessible and documented for reproducibility.

See also these helpful articles for the introduction and comparison

Edge, P., Bafna, V., & Bansal, V. (2017). HapCUT2: robust and accurate haplotype assembly for diverse sequencing technologies. Genome Research, 27(5), 801–812. https://doi.org/10.1101/gr.213462.116

Wick, R. R., Judd, L. M., & Holt, K. E. (2019). Performance of neural network basecalling tools for Oxford Nanopore sequencing. Genome Biology, 20(1), 129. https://doi.org/10.1186/s13059-019-1727-y

Reviewer #2: The paper titled “Sub-consensus haploid variant calling in long-read sequencing technology” by Xavier Zair evaluates the performance of LoFreq for detecting very low-frequency (sub-consensus) variants using Oxford Nanopore long-read sequencing data. The authors also introduce a calibration method that adjusts Phred quality scores to improve the accuracy of variant calling. Overall, the study is valuable and well-executed, but it would benefit from clearer explanations, better presentation of figures, and additional statistical and methodological details to enhance clarity and reproducibility.

MAJOR COMMENTS

1. The study only tests LoFreq. It would be stronger if the authors compared it with other current tools such as PEPPER-Margin-DeepVariant, Clair3, or Longshot2, or explained why these were not used.

2. The paper gives results for recall and false discovery rate (FDR) but does not include any statistical tests or confidence levels. The authors should add simple statistics (for example, t-tests or ANOVA) to show if the differences between flow cells or frequencies are significant

3. The part describing how Phred scores were adjusted needs clearer explanation. The authors should show how they tested the accuracy of their fitted curves (for example, by giving R² values or error plots). It should also be clear which curve fit worked best and why.

4. The paper should explain more clearly why detecting sub-consensus variants matters. For example: it can help in tracking viral mutations and quasispecies, monitoring drug resistance, or improving outbreak control and vaccine design.

5. Some details of the workflow are missing or unclear. The authors should list all software versions and random settings used.

6. The authors should confirm that all data and code mentioned in the paper are really available in the public repositories.

7. The use of both plasmid and bacterial genome experiments is interesting, but the connection between them is not clearly described. The authors should explain why both systems were chosen and how they complement each other.

8. The paper mentions 12 rounds of subsampling but does not justify why 12 was chosen. The authors should clarify if this number provides stable results or if it was an arbitrary choice.

9. The clonal plasmid data are used as a control, but there is little mention of validation with another sequencing platform (for example, Illumina or Sanger). The authors should confirm their false positive rates using an independent method for a small subset of samples

10. Figures 3–5 are difficult to interpret without reading the main text. Each figure should include a short summary line in the caption (for example, “Phred reduction of 7 eliminates false positives”).

11. The paper uses the terms sub-consensus variants, rare variants, and low-frequency variants in different sections.It would be better to choose one term and use it consistently throughout the text.

MINOR COMMENTS

1. Some paragraphs in Methods and Results are repetitive, especially about Phred-score analysis and flow-cell differences. These could be shortened.

2. The figure legends should show; sequencing depth on the x-axis, FDR or recall (%) on the y-axis, and the number of repeats (n = 12).

3. The authors must use consistent terms in Table 1 and 2 (e.g., use either “false discovery rate” or “false positives”).

4. The authors must add full software version numbers (for example, QUAD v0.2, LoFreq v2.1.3.1, Rasusa v0.6.1) in the text or supplementary data.

5. In the Abstract, the phrase “offers a practical solution” could be more specific: for example, “offers a simple calibration approach that reduces false discovery rates in ONT data.”

6. In the Introduction, the first paragraph is very long; it can be split into two parts for clarity, one about NGS use and one about variant calling challenges.

7. In the Methods, average read lengths for R9.4.1 and R10.4.1 flow cells should be added.

8. In the Results, the authors note that FDR “plateaued at 25%.” It would be good to compare this value with results from earlier studies

9. In the Discussion, the authors must clearly list the study’s main limitations, such as reduced indel accuracy and limited use for diploid genomes.

10. A single figure or schematic summarizing the workflow and the Phred calibration process would be useful!

**********

what does this mean?). If published, this will include your full peer review and any attached files.

If you choose “no”, your identity will remain anonymous but your review may still be made public.

**Do you want your identity to be public for this peer review?**  For information about this choice, including consent withdrawal, please see our Privacy Policy

Reviewer #1: No

Reviewer #2: **Yes:** Fatmahan Atalar

**********

[NOTE: If reviewer comments were submitted as an attachment file, they will be attached to this email and accessible via the submission site. Please log into your account, locate the manuscript record, and check for the action link "View Attachments". If this link does not appear, there are no attachment files.]

To ensure your figures meet our technical requirements, please review our figure guidelines: https://journals.plos.org/plosone/s/figures

You may also use PLOS’s free figure tool, NAAS, to help you prepare publication quality figures: https://journals.plos.org/plosone/s/figures#loc-tools-for-figure-preparation.

NAAS will assess whether your figures meet our technical requirements by comparing each figure against our figure specifications.

Attachment Submitted filename: Atalar comments PLOS one manuscript.docx

# Author comment: Author response to Decision Letter 1

**DOI:** 10.1371/journal.pone.0356259.r002

29 Dec 2025

Dear Reviewers,

We would like to sincerely thank you for the comments provided. We found all feedback to be highly constructive, and we are confident that the revisions have strengthened the manuscript considerably. We have addressed all suggestions in some form; where direct implementation was not applicable, we have provided alternative solutions that address the underlying concerns.

We hope you will find these revisions suitable. Notably, the study repository has undergone extensive updates, and we invite you to refer to https://github.com/x-zair/ONT_truthset for the revised README and supplementary materials.

Below, we provide point-by-point responses to each comment, along with the corresponding actions taken and line numbers where changes have been implemented (line numbers correspond to the non-markup version of the manuscript).

Thank you once again for taking the time to provide such invaluable feedback on our work. We look forward to hearing from you and hope that these revisions adequately address the points raised.

Sincerely,

Xavier Zair

Attachment Submitted filename: Response_to_reviewers.docx

# Peer review: Decision Letter 1

**DOI:** 10.1371/journal.pone.0356259.r003

**Authors:** Nihad A.M Al-Rashedi

9 Apr 2026

Dear Dr. Zair,

Thank you for submitting your manuscript to PLOS ONE. After careful consideration, we feel that it has merit but does not fully meet PLOS ONE’s publication criteria as it currently stands. Therefore, we invite you to submit a revised version of the manuscript that addresses the points raised during the review process.

Please submit your revised manuscript by May 24 2026 11:59PM. If you will need more time than this to complete your revisions, please reply to this message or contact the journal office at plosone@plos.org. When you're ready to submit your revision, log on to https://www.editorialmanager.com/pone/ and select the 'Submissions Needing Revision' folder to locate your manuscript file.

- A letter that responds to each point raised by the academic editor and reviewer(s). You should upload this letter as a separate file labeled 'Response to Reviewers'.
- A marked-up copy of your manuscript that highlights changes made to the original version. You should upload this as a separate file labeled 'Revised Manuscript with Track Changes'.
- An unmarked version of your revised paper without tracked changes. You should upload this as a separate file labeled 'Manuscript'.

If you would like to make changes to your financial disclosure, please include your updated statement in your cover letter. Guidelines for resubmitting your figure files are available below the reviewer comments at the end of this letter.

If applicable, we recommend that you deposit your laboratory protocols in protocols.io to enhance the reproducibility of your results. Protocols.io assigns your protocol its own identifier (DOI) so that it can be cited independently in the future. For instructions see: https://journals.plos.org/plosone/s/submission-guidelines#loc-laboratory-protocols. Additionally, PLOS ONE offers an option for publishing peer-reviewed Lab Protocol articles, which describe protocols hosted on protocols.io. Read more information on sharing protocols at https://plos.org/protocols?utm_medium=editorial-email&utm_source=authorletters&utm_campaign=protocols.

As the corresponding author, your ORCID iD is verified in the submission system and will appear in the published article. PLOS supports the use of ORCID, and we encourage all coauthors to register for an ORCID iD and use it as well. Please encourage your coauthors to verify their ORCID iD within the submission system before final acceptance, as unverified ORCID iDs will not appear in the published article. *Only*  the individual author can complete the verification step; PLOS staff *cannot*  verify ORCID iDs on behalf of authors.

We look forward to receiving your revised manuscript.

Kind regards,

Nihad A.M Al-Rashedi

Academic Editor

PLOS One

Journal Requirements:

If the reviewer comments include a recommendation to cite specific previously published works, please review and evaluate these publications to determine whether they are relevant and should be cited. There is no requirement to cite these works unless the editor has indicated otherwise.

Please review your reference list to ensure that it is complete and correct. If you have cited papers that have been retracted, please include the rationale for doing so in the manuscript text, or remove these references and replace them with relevant current references. Any changes to the reference list should be mentioned in the rebuttal letter that accompanies your revised manuscript. If you need to cite a retracted article, indicate the article’s retracted status in the References list and also include a citation and full reference for the retraction notice.

[Note: HTML markup is below. Please do not edit.]

Reviewers' comments:

Reviewer's Responses to Questions

**Comments to the Author**

Reviewer #2: (No Response)

Reviewer #3: (No Response)

**********

2. Is the manuscript technically sound, and do the data support the conclusions?

Reviewer #2: Yes

Reviewer #3: Yes

**********

3. Has the statistical analysis been performed appropriately and rigorously?

Reviewer #2: No

Reviewer #3: Yes

**********

4. Have the authors made all data underlying the findings in their manuscript fully available?

The PLOS Data policy

Reviewer #2: Yes

Reviewer #3: Yes

**********

5. Is the manuscript presented in an intelligible fashion and written in standard English?

Reviewer #2: Yes

Reviewer #3: Yes

**********

Reviewer #2: COMMENTS

1. The Statistical Analysis section states confidence intervals are calculated with n=3 replicates per condition (df=2), yet Methods and Results repeatedly refer to 12 rounds/replicates and figures/tables state (n=12). The authors should define precisely what constitutes a “replicate” versus a “subsample/iteration” (e.g., independent libraries/flow cells vs computational subsampling replicates), then make all n values consistent across Methods, Results, Tables/Figures, and CI calculations. If CIs in Tables are based on n=12, the Statistical Analysis text must reflect that (and vice versa)..

2. The authors should clarify whether subsampling was performed with a fixed random seed. If yes, they have to report the seed (or where it is set in the pipeline). If not, they must state that subsampling is stochastic and either (a) justify why this does not affect conclusions or (b) report variability across runs.

3. The revision appropriately discusses limitations for indel calling and low allele fractions. However, the manuscript should include a clear, explicit statement regarding the intended scope (haploid/sub-consensus contexts) and that the approach is not validated for diploid germline variant calling, e.g., “This approach is designed and validated for haploid sub-consensus variant detection and has not been evaluated for diploid germline variant calling.” etc..

4. The Results include a brief comparison and note that Longshot/Clair3 are not designed for sub-consensus haploid calling. The authors should add 2–3 sentences in the main Discussion clarifying (i) why broader ONT variant-caller benchmarking is limited for this use case and (ii) what conclusions can and cannot be drawn from the supplementary comparison.

5. The authors must standardize terminology across the manuscript (choose one primary term such as ‘sub-consensus variants’ or ‘low-frequency variants’) and use alternative terms only once as synonyms to avoid confusion.

6. While effect sizes (Cohen’s d) are now mentioned in Methods, Results still report SD in places, while other sections refer to CI.

The authors need to

• use a single reporting standard (either SD or 95% CI) consistently across Results, Tables, and Figures;

• report effect sizes alongside p-values wherever inferential tests are used;

• add one brief sentence confirming that test assumptions were checked (or state which nonparametric alternatives were used if assumptions were not met); and

• ensure the paired vs unpaired tests match the study design and that the reported degrees of freedom are consistent with the final sample size definition.

Reviewer #3: This is a useful pragmatic study validating and configuring LoFreq for use with ONT nanopore sequencing, an important area for pathogen genomics where other tools have not yet been developed. I am content the authors have presented their findings and supported their conclusions fairly. This has come after some major revisions from comments by other reviewers.

Two minor comments that need to be addressed before publication:

Line 253. Uses allele frequency 0.1, 0.15, 0.19. However Table 1 uses 5%, 10%, 15%, 19%. Pick one for consistency and ease of understanding.

Figure 3b, emphyrical was used on the Y-axis instead of empirical

**********

what does this mean?). If published, this will include your full peer review and any attached files.

If you choose “no”, your identity will remain anonymous but your review may still be made public.

**Do you want your identity to be public for this peer review?**  For information about this choice, including consent withdrawal, please see our Privacy Policy

Reviewer #2: **Yes:** Fatmahan Atalar

Reviewer #3: No

**********

[NOTE: If reviewer comments were submitted as an attachment file, they will be attached to this email and accessible via the submission site. Please log into your account, locate the manuscript record, and check for the action link "View Attachments". If this link does not appear, there are no attachment files.]

To ensure your figures meet our technical requirements, please review our figure guidelines: https://journals.plos.org/plosone/s/figures

You may also use PLOS’s free figure tool, NAAS, to help you prepare publication quality figures: https://journals.plos.org/plosone/s/figures#loc-tools-for-figure-preparation.

NAAS will assess whether your figures meet our technical requirements by comparing each figure against our figure specifications.

Attachment Submitted filename: PLOS 2nd revision ONT.docx

# Author comment: Author response to Decision Letter 2

**DOI:** 10.1371/journal.pone.0356259.r004

18 May 2026

Dear reviewers,

We would like to start by once again expressing our sincere thanks for taking the time to read and address areas of improvement in our manuscript. We find the comments provided during the second review to be as constructive as the last review and we are happy to address the points mention.

We are pleased that the manuscript was received positively overall, and we believe the suggested addition has overall strengthen the interpretability and general quality of the study. We hope that you find the revisions to be sufficient and we look forward to hearing your conclusions.

Please find a point-by-point response to the remaining issues raised, the line locations mentioned are made with reference to the ‘tracked changes accepted’ version of the manuscript.

It has been a pleasure corresponding with you, and we look forward to hearing your decision on the manuscript.

Sincerely,

Xavier Zair

Graduate Student

Saw Swee Hock School of Public Health

National University of Singapore

Attachment Submitted filename: Response_to_reviewers_2.docx

# Editor report: Decision Letter 2

**DOI:** 10.1371/journal.pone.0356259.r005

**Authors:** Nihad A.M Al-Rashedi

2 Aug 2026

Sub-consensus haploid variant calling in Long-read sequencing technology

PONE-D-25-47549R2

Dear Dr. Zair,

We’re pleased to inform you that your manuscript has been judged scientifically suitable for publication and will be formally accepted for publication once it meets all outstanding technical requirements.

Within one week, you’ll receive an e-mail detailing the required amendments. When these have been addressed, you’ll receive a formal acceptance letter and your manuscript will be scheduled for publication.

An invoice will be generated when your article is formally accepted. Please note, if your institution has a publishing partnership with PLOS and your article meets the relevant criteria, all or part of your publication costs will be covered. Please make sure your user information is up-to-date by logging into Editorial Manager at Editorial Manager® and clicking the ‘Update My Information' link at the top of the page. For questions related to billing, please contact billing support.

If your institution or institutions have a press office, please notify them about your upcoming paper to help maximize its impact. If they’ll be preparing press materials, please inform our press team as soon as possible -- no later than 48 hours after receiving the formal acceptance. Your manuscript will remain under strict press embargo until 2 pm Eastern Time on the date of publication. For more information, please contact onepress@plos.org.

Kind regards,

Nihad A.M Al-Rashedi

Academic Editor

PLOS One

Additional Editor Comments (optional):

Reviewers' comments:

# Editor report: Acceptance letter

**DOI:** 10.1371/journal.pone.0356259.r006

**Authors:** Nihad A.M Al-Rashedi

PONE-D-25-47549R2

PLOS One

Dear Dr. Zair,

I'm pleased to inform you that your manuscript has been deemed suitable for publication in PLOS One. Congratulations! Your manuscript is now being handed over to our production team.

At this stage, our production department will prepare your paper for publication. This includes ensuring the following:

* All references, tables, and figures are properly cited

* All relevant supporting information is included in the manuscript submission,

* There are no issues that prevent the paper from being properly typeset

You will receive further instructions from the production team, including instructions on how to review your proof when it is ready. Please keep in mind that we are working through a large volume of accepted articles, so please give us a few days to review your paper and let you know the next and final steps.

Lastly, if your institution or institutions have a press office, please let them know about your upcoming paper now to help maximize its impact. If they'll be preparing press materials, please inform our press team within the next 48 hours. Your manuscript will remain under strict press embargo until 2 pm Eastern Time on the date of publication. For more information, please contact onepress@plos.org.

You will receive an invoice from PLOS for your publication fee after your manuscript has reached the completed accept phase. If you receive an email requesting payment before acceptance or for any other service, this may be a phishing scheme. Learn how to identify phishing emails and protect your accounts at https://explore.plos.org/phishing.

If we can help with anything else, please email us at customercare@plos.org.

Thank you for submitting your work to PLOS One and supporting open access.

Kind regards,

PLOS ONE Editorial Office Staff

on behalf of

Dr. Nihad A.M Al-Rashedi

Academic Editor

PLOS One
